# Supplementary material for: Tobacco use among designated air pollution victims and its association with lung function and respiratory symptoms: a retrospective cross-sectional study
Source: BMJ Open. 2014 Jul 31;4(7):e005393. doi: 10.1136/bmjopen-2014-005393 (PMC4120398; doi:10.1136/bmjopen-2014-005393)
Supplement: Author's manuscript [file bmjopen-2014-005393.draft_revisions.pdf]

Tobacco use among designated pollution victims and its impact on lung function and respiratory symptoms

Journal:	*BMJ Open*
Manuscript ID:	bmjopen-2014-005393
Article Type:	Research
Date Submitted by the Author:	03-Apr-2014

Complete List of Authors:	kotaki, kenji; Nagasaki University Graduate School of Biomedical Sciences, Course of Cardiopulmonary Rehabilitation Science<br>Senjyu, Hideaki; Nagasaki University Graduate School of Biomedical Science, Course of Cardiopulmonary Rehabilitation Science<br>Tanaka, Takako; Nagasaki University Graduate School of Biomedical Science, Course of Cardiopulmonary Rehabilitation Science<br>Yano, Yudai; Nagasaki University Graduate School of Biomedical Science, Course of Cardiopulmonary Rehabilitation Science<br>Miyamoto, Naomi; Nagasaki University Graduate School of Biomedical Science, Course of Cardiopulmonary Rehabilitation Science<br>Nishinakagawa, Tsuyoshi; Nagasaki University Graduate School of Biomedical Science, Course of Cardiopulmonary Rehabilitation Science<br>Yanagita, Yorihide; Nagasaki University Graduate School of Biomedical Science, Course of Cardiopulmonary Rehabilitation Science<br>Asai, Masaharu; Nagasaki University Graduate School of Biomedical Science, Course of Cardiopulmonary Rehabilitation Science<br>Kozu, Ryo; Nagasaki University Hospital, Department of Rehabilitation Medicine<br>Tabusadani, Mitsuru; Nagasaki University, Center for Industry,University and Government Cooperation<br>Sawai, Terumitsu; Nagasaki University Graduate School of Biomedical Science, Course of Cardiopulmonary Rehabilitation Science<br>Honda, Sumihisa; Nagasaki University Graduate School of Biomedical Science, Course of Cardiopulmonary Rehabilitation Science

**Primary Subject Heading**:	Smoking and tobacco
Secondary Subject Heading:	Epidemiology, Respiratory medicine
Keywords:	EPIDEMIOLOGY, SOCIAL MEDICINE, Health & safety < HEALTH SERVICES ADMINISTRATION & MANAGEMENT, Public health < INFECTIOUS DISEASES, Chronic airways disease < THORACIC MEDICINE, PUBLIC HEALTH

Title

Tobacco use among designated pollution victims and its impact on lung function and respiratory symptoms

Authors (* corresponding author)

Kenji Kotaki, Hideaki Senjyu*, Takako Tanaka, Yudai Yano, Naomi Miyamoto, Tsuyoshi Nishinakagawa, Yorihide Yanagita, Masaharu Asai, Ryo Kozu, Mitsuru Tabusadani, Terumitsu Sawai, Sumihisa Honda,

Author details

Department of Cardiopulmonary rehabilitation Science, Nagasaki University Graduate School of Biomedical Sciences, Nagasaki, Japan

For peer review only

The postal address

Department of Cardiopulmonary Rehabilitation Science, Nagasaki University Graduate School of Biomedical Sciences, 1-7-1 Sakamoto, Nagasaki 852-8520, Japan.

Contact

Email :

senjyu@nagasaki-u.ac.jp

Tel: +81-95-819-7963

Fax: +81-95-819-7963

Keywords: Air pollution, Tobacco, Respiratory function, Respiratory symptoms, victims

Word count: 3521 words

For peer review only

ABSTRACT

Objective: We sought to elucidate the long-term effects of tobacco use in designated pollution victims with and without obstructive pulmonary defects.

Design: A retrospective cross-sectional study.

Setting: The register of pollution victims in Kurashiki, Japan.

Participants: 730 individuals over 65 years of age previously diagnosed with pollution-related respiratory disease. Patients were classified into four groups according to their smoking status and whether or not they had obstructive pulmonary disease. We then compared the prevalence of respiratory symptoms and lung function over time between groups.

Primary outcome measures: Spirometry was performed and a respiratory health questionnaire completed in the same season each year for up to 30 years.

Results: Rates of smoking and respiratory disease were high in our sample. While respiratory function in non-smoking patients did not completely recover, the annual rate of change in lung function was within the normal range ($p < 0.01$). However, smokers had worse lung function and were more likely to report more severe pulmonary symptoms ($p < 0.01$).

Conclusions: Patients' respiratory function did not fully recover despite improved air

For peer review only

quality. Our results suggest that, in the context of exposure to air pollution, tobacco use causes additional loss of lung function and exacerbates respiratory symptoms.

Article summary

Strengths and limitations

・This first study to have analyzed changes in respiratory function and respiratory symptoms in a group of designated pollution victims in Japan over up to 30 years.

・Tobacco use caused additional loss of lung function and exacerbated respiratory symptoms in those who have been exposed to air pollution.

・However, we did not have access to the amount and duration of cigarette smoking, or individual-level exposure to environmental pollutants, much of which may have been occupational.

・We were also unable to account for possible confounding factors such as socio-economic class and other lifestyle factors.

For peer review only

INTRODUCTION

As many Asian nations, particularly China, are currently experiencing rapid economic growth, air pollution is increasingly being recognized as an important emerging environmental and public health issue. Previous work has shown that levels of ambient air pollution are associated with an elevated incidence of respiratory diseases and reported prevalence of respiratory symptoms,[1] particularly asthma, pulmonary emphysema and chronic bronchitis.[2] Atmospheric pollution caused by PM 2.5, fine particulate matter with a diameter of 2.5 micrometers or less, is reported to be particularly acute in China and in surrounding countries as a result of wind dispersal. This is a growing public health issue in the region, especially given that PM 2.5 is capable of penetrating deep into lung tissue and precipitating a number of respiratory diseases. [3]

Japan was also severely impacted during its period of accelerated economic growth in the wake of postwar reconstruction in the 1960s, with both air pollution and the prevalence of respiratory diseases rising markedly during this period. In Kurashiki, a city located in Okayama Prefecture, reports of pulmonary toxicity owing to oxidant air

For peer review only

pollutants rose by a factor of 1.73 among the city's inhabitants in the period leading up to 1970 as yearly average SO3 levels increased. The majority of excess pollution-related deaths in this period were caused by acute asthma.

Although patients do not commonly recover fully from pollution-related respiratory diseases, FEV1/ FVC ratios (calculated by dividing volume exhaled at the end of the first second of forced expiration by Forced Vital Capacity) may return to within the normal range once air quality is improved.[4] As a result, Japan passed the Pollution-Related Health Damage Compensation Law in 1973 with the aim of identifying designated air pollution victims within affected areas for compensation. Patients with a diagnosis of bronchial asthma, chronic bronchitis or pulmonary emphysema who had been resident in any designated air pollution zone for a specified period of time were legally recognized as pollution victims. These designated victims were fully reimbursed the costs of all related medical treatment by the state.However, patients' lung function, as measured using FEV1/FVC ratios, was not among the diagnostic criteria used to determine eligibility for compensation as no agreed clinical definition for Chronic Obstructive Pulmonary Disease (COPD) existed at that time.

Tobacco smoke has been shown to contain high levels of PM 2.5, and is likely to have a

For peer review only

number of effects in common with air pollution. In addition, it has been reported that ambient levels of PM 2.5 can reach 200–800μg /m^3 in some indoor spaces where smoking is permitted. [5] These levels similar to those recorded in designated air pollution zones in Japan. Tobacco use has been shown to reduce lung function, as measured using FEV1/ FVC ratios, and has been identified as a cause of COPD and other tobacco-related diseases – in addition to contributing to early mortality. The World Health Organization (WHO) Framework Convention on Tobacco Control was therefore adopted in 2003 as part of an effort to decrease rates of smoking worldwide. [6] WHO figures show that rates of smoking in Japan have fallen from 83.7% at their peak in 1965 to 32.7% in 2013. [7] However, tobacco use remains a significant public health issue. According to a WHO report, more than 1.1 billion people globally are regular smokers, including one third of the total world population over 15 years of age. [8,9] Furthermore, Asia continues to account for a third of total world cigarette consumption, with China responsible for more cigarette production and consumption than any country. [8]

Both air pollution and tobacco smoke have been shown to reduce patients' FEV1/FVC ratios, to increase the risk of obstructive ventilatory defects, and to exacerbate existing respiratory symptoms. In contrast, Tanaka et al. have reported that when air quality is

For peer review only

improved, the annual change in patients' FEV1 can return to within the normal range. [4] However, the effect of continuing tobacco use on respiratory function and pulmonary symptoms in patients with already reduced FEV1/FVC ratios as a result of air pollution has yet to be elucidated.

The purpose of the present study, carried out among a population of confirmed pollution victims living in a designated pollution-affected area, was to determine the effect of tobacco use on measures of respiratory function and prevalence of pulmonary symptoms up to 30 years after an improvement in air quality. In particular, we sought to compare these effects in patients with and without diagnosed obstructive ventilatory defects resulting from exposure to air pollution.

For peer review only

METHODS

Study design and setting

The present study was embedded in a retrospective cross-sectional analysis of lung function and respiratory symptoms in designated victims of pollution-related illness in Kurashiki between their initial evaluation (from 1976–1988) and follow-up in 2009. Study subjects were drawn from the register of pollution victims in Kurashiki. All study participants met the following conditions as determined by the Public Relief System of Kurashiki City, in accordance with the Pollution-Related Health Damage Special Measures Law (1969) and the Pollution-Related Health Damage Compensation Law (1973): (1) they had resided or had employment within a designated air pollution zone (Table 1), and (2) they were diagnosed with chronic bronchitis, asthma, or emphysema by a medical practitioner.

In accordance with the Public Nuisance Countermeasures Law (1967), registered victims were entitled to various forms of compensation including a monthly consultation with a doctor, yearly assessments of respiratory symptoms consisting of a detailed

For peer review only

questionnaire and spirometry tests, and prescriptions for expectorants and bronchodilators. Of a total of 419,203 residents (204,958 males, 214,245 females) 3,838 (0.9% of the total population) were officially classified as designated pollution victims in the period up to 1988. The records of these 3,838 residents were reviewed in 2009 with the permission of the Kurashiki City Public Office (Figure 1). Of the 1,392 remaining survivors, we screened 774 of those aged ≥ 65 years for inclusion in the study. After removing 44 records which did not have complete spirometry data, the resulting sample size was 730.

We classified patients into two groups according to whether they had an obstructive impairment using the FEV1/FVC ratios recorded at their initial evaluation. Participants in each group were then further sub-divided according to whether they had ever smoked or not – determined using self-reporting. This resulted in four comparison groups, labelled as OS (smokers and ex-smokers diagnosed with an obstructive impairment reporting), ONS (non-smokers diagnosed with an obstructive impairment), NOS (smokers and ex-smokers without a diagnosed obstructive impairment) and NONS (non-smokers without an obstructive impairment). The majority of these patients were diagnosed with chronic bronchitis (n = 528, 68.2%), asthma (n = 242, 31.3%), or

For peer review only

emphysema (n = 4, 0.5%), as described in case reports provided by authorized medical practitioners.

The study protocol was approved by the Ethical Committee of the Nagasaki University Graduate School of Biomedical Sciences.

Clinical measurements

Patients' gender, height, weight, Body Mass Index (BMI), Forced Vital Capcity (FVC), Vital Capacity (%VC), Forced Expiratory Volume after the first second of forced expiration (FEV1), FEV1/ FVC ratios, diagnosed respiratory conditions and respiratory symptoms were all recorded at baseline and at follow-up.

Spirometry measurements

Spirometry was performed by trained staff at the Mizushima-Kyodo Hospital using an electronic spirometry device (FUDAC 70, Fukuda Sangyo Inc., Chiba, Japan). Tests were performed on patients in the sitting position, and were repeated until at least

For peer review only

three reproducible forced expiratory curves had been obtained. Both FEV1 and FVC were measured, and used to calculate FEV1/FVC ratios, in addition to %VC using the method proposed by Baldwin et al. [10]

Respiratory symptoms

Respiratory symptoms (including dyspnea, asthma attacks, cough, and sputum production) were assessed by physicians in the same season each year using a respiratory health questionnaire designed by the government of Japan. Each symptom was graded using a standardized five-point scale.

Dyspnea

1. Too breathless to leave the house, or breathless when dressing or undressing.
2. Breathless after walking about 50m or after a few minutes on level ground.
3. Breathless when walking on level ground and keeping up with people of the same age, but not breathless when walking at own pace.
4. Breathless when walking up a slight hill or the stairs
5. Breathless only during strenuous exercise.

For peer review only

Wheeze

1. Severe episode ≥ 10 days each month during the last year.

2. Severe episode ≥ 5 days each month during the last year, or mild episode ≥ 10 days each month during the last year.

3. Severe episode ≥ 1 day each month during the last year, or mild episode ≥ 5 days each month during the last year.

4. Mild episode ≥ 1 day each month during the last year.

5. No episodes of wheezing.

Cough and sputum

1. Daily cough and sputum, with a large amount of sputum or difficulty clearing sputum.

2. Daily cough and sputum, with a moderate amount of sputum or difficulty clearing sputum.

3. Daily cough and sputum, but not troublesome during daily life.

4. Daily cough and sputum for ≤ 3 months each year.

5. No cough or sputum.

For peer review only

Air pollution monitoring

Data on mean daily concentrations of air pollutants were obtained at 21 points in Kurashiki. While measurement of SO2 concentrations started in 1965, monitoring of nitrogen dioxide (NO2) concentrations commenced in 1971.

Statistical analysis

Data from patients in each of the four comparison groups were compared using the Kruskal-Wallis test and Mann-Whitney U test as a post-hoc test. Differences in gender distributions and the prevalence of respiratory diagnoses in each group were analyzed using the Cochran-Armitage test, using the chi-square test to test for statistical significance. Changes in lung function between initial evaluation and follow-up were analyzed using the Wilcoxon signed-rank test and the Mann-Whitney U test as a post-hoc test. A normality test was performed on all continuous variables using the Kolmogorov-Smirnov Test prior to analysis. All analyses were performed using PASW Statistics, version 18. Finally p values of <0.05 were considered statistically significant.

For peer review only

RESULTS

Sample characteristics

Table 2 shows the characteristics of our patient sample at initial evaluation. While we found significant differences (p<0.001) in the age distributions between groups (the mean ages were 54.3±8.3, 49.6±8.2, 49.8±7.8 and 51.3±8.9 in the OS, ONS, NOS and NONS groups respectively) males outnumbered females in the OS and NOS groups (p<0.001). Furthermore patients in the ONS and NONS groups had significantly lower heights and weights (p<0.001)

We found differences in FVC values between groups, with patients in the ONS and NONS groups presenting significantly lower values than those in the OS and NOS groups (3.04±0.79 and 2.94±0.68 versus 3.37±0.79 and 3.49±0.76, p<0.001). FEV1 values, which also varied significantly between comparison groups (p<0.001) were found to be lowest in the ONS group (1.72±0.58ml), followed by those in the OS group (2.09±0.64ml). Furthermore, %VC and FEV1/FVC ratios were lowest in the OS and ONS groups. Finally, the prevalence of bronchial asthma at initial evaluation(57.9%) was much

For peer review only

higher in the ONS compared with all other groups ($p<0.001$).

Change in respiratory function and symptoms between initial evaluation and follow-up

Table 3 shows the results of our evaluation of the change in severity of respiratory symptoms and respiratory function between initial evaluation and follow-up. Respiratory function was found to have deteriorated in almost all subjects by follow-up. In particular, FEV/FVC ratios in the NOS group, which averaged 79.1±5.6% at baseline, had fallen to 67.9±10.2% at follow-up as a result of the development of obstructive ventilatory defects in a number of subjects. Conversely, average FEV/FVC ratios were found to have increased from 59.8±10.3% to 62.1±13.4% in the ONS group ($p<0.001$).With respect to respiratory symptoms, these had worsened on average from level II to level III. However, the NONS group saw a decrease in the proportion of patients reporting asthma attacks ($p=0.78$), cough, and sputum production ($p=0.24$) over the period studied.

For peer review only

Mean annual changes in respiratory function

Table 4 shows mean annual changes of respiratory function in each comparison group. Mean FEV1 was found to have decreased to a similar extent in both the OS (-36.7±19.1ml/year) and NOS groups (-37.2± 16.4ml/year). While there was a slight fall in FEV1 among the NONS group (-25.5 ±17.2 ml/year), there was a marginal improvement in FEV1 (26.5±18.5ml/year) among patients in the ONS group ($p<0.001$). At the same time, while FEV1/FVC ratios fell ($p<0.001$) in both the OS (-0.04±0.05) and NOS (-0.04±0.03) groups, an improvement was seen among the ONS group (0.09±0.06%). Finally, VC % fell among patients in all groups ($p<0.001$), although the decline was greater in the OS (-0.05±0.08) and ONS (-0.05±0.08) groups than in the NOS (-0.03±0.06) and NONS (-0.03±0.07) groups; as shown in Table 3.

Comparison of respiratory symptoms at final evaluation

Table 5 shows the severity of respiratory symptoms in our four comparison groups at follow-up.

In the OS and ONS groups, whose subjects were diagnosed with obstructive ventilatory

For peer review only

defects at baseline, the great majority reported severe dyspnea, asthma, cough and sputum production. Severe dyspnea was also highly prevalent among the OS and ONS groups. Furthermore, while severity of asthma was high in both the ONS and NONS groups at initial evaluation, the proportion of patients reporting severe asthma had increased in the OS and NOS groups at follow-up ($p<0.001$). While severity of asthma was similar between the NOS and NONS groups at follow-up, prevalence of severe cough and sputum production had increased in the OS and NOS groups with 80% of subjects in the former reporting scores of I to III due to a worsening of symptoms ($p<0.001$).

For peer review only

DISCUSSION

This retrospective cross-sectional study analyzed changes in respiratory function and respiratory symptoms between two time points in time (1976 and 2008) among a group of designated pollution victims in Japan. Our results show for the first time that improvements in respiratory function and reductions in respiratory symptoms are prevented by continuing tobacco use.

Among those patients diagnosed with an obstructive impairment due to air pollution exposure, we found that FEV1/FVC ratios had deteriorated by follow-up with no reduction in morbidity. However, our results showed an improvement in FEV1/FVC ratios in the ONS group, which could be attributed to the high proportion of subjects diagnosed with bronchial asthma at initial evaluation (57.9%) when compared with the other groups. These patients received treatment with bronchodilators such as inhaled corticosteroids, long-acting bronchial anti-cholinergic agents and long-acting ß2 agonists, which may have resulted in improvements in lung function.[11] We also observed that respiratory function had deteriorated among patients in the OS group despite the improvement in local air quality, resulting in depressed FEV1/FVC ratios

For peer review only

(54.5±12.3%). Furthermore, while FEV1/FVC ratios among the NOS group were relatively low at the time of initial diagnosis, continuing tobacco use had resulted in some cases in obstructive impairment, causing FEV1/FVC ratios at follow-up to decrease to 67.9±10.2%.

While air pollution can cause airway obstruction, resulting in and asthenia, reactive airway dysfunction syndrome and reduced lung function, tobacco use, which can result in airway hyper-responsiveness, has been shown to exert a stronger influence on respiratory health by a number of studies.[12], [13] These effects were evident in the OS and NOS groups, which showed similar annual rates of change in respiratory function over time. Conversely, Downs et al. have shown that, in non-smokers, annual rates of change in FEV1can return to within the normal range in response to an improvement in air quality. [14]. These findings are reflected in the results of the present study. Previous studies have reported a mean annual rate of change in FEV1 of between -30 and -22 ml/year in healthy, non-smoking males and females aged > 65 years[15] However, exposure to air pollution and tobacco smoke, both of which can result in tracheobronchial injury, can worsen annual rates of change in lung function markedly.

For peer review only

A high proportion of our subjects, particularly those with obstructive impairment, reported respiratory symptoms at baseline. These symptoms were also more pronounced among the smokers in our sample. In the NONS group, however, the proportion of patients reporting asthma attacks, sputum production and cough had not increased – suggesting that these symptoms may be prevented by smoking cessation. The proportion of subjects in the ONS group reporting asthma attacks had increased by follow-up. Furthermore, the proportion of subjects reporting asthma attacks in the NOS group had increased to the same levels as that found in the NONS group at initial evaluation. Repeated bronchial inflammation caused by smoking and pollutant exposure can result in thickening of the bronchial wall and airway remodelling which cannot be revered even by steroid treatment following the onset of hypersensitivity. [16] Furthermore, Siroux et al. have shown that continued tobacco use can significantly increase the incidence of asthma attacks.[17] As a result of this association, smoking is listed as a potential trigger for asthma in asthma management guidelines. [18] Tobacco use is also likely to have been the cause of the higher prevalence of severe cough and sputum production found in the OS and NOS groups.

A number of studies have shown smoking can causes respiratory tract inflammation

For peer review only

and exacerbate symptoms such as cough and sputum production.[19] This was reflected in our finding that the severity of dyspnea among subjects in the OS and ONS groups had increased markedly at follow-up. While a number of causes of dyspnea have been identified, including age, poor physical fitness, muscle atrophy and social and environmental factors, we could not identify the primary determinants of dyspnea in the present study.[20] Finally, progression of respiratory disease and a decline in lung function can further aggravate these symptoms. [21] In Asia, increased mortality and morbidity owing to lung cancer, COPD, asthma and heart disorders owing to rising atmospheric PM 2.5 concentrations is a growing public health concern. According to the Organization for Economic Cooperation and Development, air pollution is expected to become the leading cause of death worldwide by 2050, and is likely to be responsible for between three and six million deaths annually. [22] Furthermore, Pope et al. have reported that worldwide pollution-related deaths could rise by 6% for each additional 10 μg/m3 increase in atmospheric PM 2.5concentrations. [23]

The present study emphasizes the need to prevent or reduce air pollution and to improve public education on the negative health effects of smoking in order to reduce pulmonary morbidity on the population level. This is especially the case among

For peer review only

designated victims, for whom additional education programs, behavioral interventions and support for smoking cessation would be particularly beneficial. One such behavioral intervention that has been proven to be cost-effective in reducing smoking prevalence is the five-step Global Initiative for Obstructive Lung Disease (or GOLD) framework (ask, advise, assess, assist, arrange) [24] This strategy, which aims at delaying the progress of COPD through continuing support for smoking cessation, may be applicable for use in similarly-affected communities. [25]

This study has several limitations, however. First, the information on numbers of cigarettes smoked and individual-level exposure to environmental pollutants was not available. Second, it was not within the scope of the present study to explore the role of gender-specific differences in exposure to tobacco smoke and pollutants – much of which may have been occupational. Finally, we were unable to account for possible confounding factors such as socio-economic class and lifestyle factors, which again may have resulted in individual-level differences in exposure to environmental pollutants.

Further work is needed to inform future prevention and treatment programs to improve quality-of-life of the remaining designated pollution victims in Japan. The results of the

For peer review only

present study are of particular importance in the context of high rates of smoking and worsening air quality in a number of Asian countries as they undergo rapid economic change.

For peer review only

Table 1. Time required for certification of pollution-related illness

Illness	a)	b)	c)
Chronic bronchitis	24 months	48 months	36 months
Asthma	12 months	30 months	18 months
Emphysema	36 months	66 months	52 months

a) Individual who resided in a designated area prior to 1973.

b) Individual who did not reside in a designated area, but spent at least 8 hours per day in a designated area.

c) Individual who resided in a designated area, then relocated but continued to work in a designated area for at least 8 hours per day.

For peer review only

Table 2. Sample characteristics at initial evaluation

Initial evaluation

		OS Group (n=48)	ONS Group (n=169)	NOS Group (n=119)	NONS Group (n=394)	p	post-hoc test
Age		54.3 ± 8.3	49.6 ± 8.2	49.8 ± 7.8	51.3 ± 8.9	<.001	**.†.tt
Gender (male/female)		35/13	66/103	81/38	90/304	<.001	*.**. †.†† .t.tt
Height	(cm)	161.7 ± 6.3	155.2 ± 7.6	161.4 ± 6.1	152.8 ± 7.5	<.001	*.**. †† .t.tt
Weight	(kg)	56.9 ± 6.4	55.4 ± 9.3	59.1 ± 8.9	55.2 ± 9.7	<.001	**.tt
BMI		21.8 ± 1.9	22.8 ± 3.6	22.6 ± 2.8	23.4 ± 3.8	<.001	*.** .††. t.
FVC	(l)	3.37 ± 0.79	3.04 ± 0.79	3.49 ± 0.76	2.94 ± 0.68	<.001	*.**. t.tt
%VC	(%)	105.5 ± 18.7	103.5 ± 18.5	108.3 ± 15.5	107.6 ± 17.3	<.05	††.tt
FEV1	(l)	2.09 ± 0.64	1.72 ± 0.58	2.67 ± 0.58	2.24 ± 0.58	<.001	*.**.† .††.tt
FEV1/FVC	(%)	64.9 ± 6.5	59.8 ± 10.3	79.1 ± 5.6	79.6 ± 7.1	<.001	*.† .††. t.tt
Diagnosed pulmonary disease CB,BA,PE CB+BA CB+PE, BA+PE	no	32/10/1/5/ 0/ 0	52/98/3/11/3/2	98/14/0/7/ 0/ 0	249/99/0/46/0/0	<.001	*.† .††.tt
Unit Score							
Dyspnea	1	0 (0.0%)	1(0.6%)	0 (0.0%)	0 (0.0%)		
	2	0 (0.0%)	4 (2.4%)	0 (0.0%)	4 (1.1%)		
	3	9 (18.8%)	31 (18.3%)	7 (5.9%)	55 (15.8%)	<.001	**.†.††.tt
	4	30 (62.5%)	111 (65.7%)	75 (63.0%)	246 (70.5%)		
	5	9 (18.8%)	22 (13.0%)	37 (31.1%)	89 (25.5%)		
Asthma attack	1	0 (0.0%)	1 (0.6%)	0 (0.0%)	1 (0.3%)		
	2	0 (0.0%)	8 (4.7%)	1 (0.8%)	13 (3.3%)		
	3	7 (14.6%)	51 (30.2%)	11 (9.2%)	66 (16.8%)	<.001	*.**.††.tt
	4	14(29.2%)	76 (45.0%)	30 (25.2%)	139 (35.3%)		
	5	27(56.3%)	33 (19.5%)	77 (64.7%)	175 (44.4%)		
Cough and phlegm	1	0 (0.0%)	2 (1.2%)	0 (0.0%)	0 (0.0%)		
	2	0 (0.0%)	5 (3.0%)	1 (0.8%)	9 (2.3%)		
	3	24 (50.0%)	50 (29.6%)	58 (48.7%)	143 (36.3%)	<.05	*.**.††.tt
	4	23 (47.9%)	90 (53.3%)	58 (48.7%)	225 (57.1%)		
	5	1 (2.1%)	22 (13.0%)	1 (0.8%)	15 (3.8%)		

For peer review only

Figures are presented as mean ±SD

FEV1: Forced Expiratory Volume in 1 second, FVC: Forced Vital Capacity, VC%: % Vital Capacity

FEV1/FVC (%) Forced Expiratory Volume in 1 second.

CB: Chronic Bronchitis BA : Bronchial Asthma PE: Pulmonary Emphysema

CB+BA CB+PE BA+PE

Differences in sample characteristics analyzed using the Kruskal-wallis test with Cochran-Armitage tests for significance

post-hoc tests: Mann-whitney U test, chi-square test for significance

*: OS Group vs ONS Group

**: NOS Group vs NONS Group

†: OS Group vs NOS Group

††: ONS Group vs NONS Group

t: OS Group vs NONS Group

tt :Group ONS vs NOS Group

*,**: Current smokers / ex-smokers (OS and NOS Groups) vs never smokers (ONS and NONS Groups)

†, ††: Diagnosis of obstructive ventilatory defect (OS and ONS Groups) vs no obstructive ventilatory defect (NOS and NONS Groups)

For peer review only

Table 3. Respiratory function and symptoms of designated pollution victims between initial evaluation and follow-up

		Initial evaluation	Follow-up	p	Initial evaluation	Follow-up	p	Initial evaluation	Follow-up	p	Initial evaluation	Follow-up	p
		**OS Group**			**ONS Group**			**NOS Group**			**NONS Group**		
Age		54.3 ± 8.3	78.6 ± 6.5	<.001	49.6 ± 8.2	77.3 ± 7.5	<.001	49.8 ± 7.8	74.2 ± 7.1	<.001	51.3 ± 8.9	76.5 ± 7.8	<.001
Height	(cm)	161.7 ± 6.3	159.3 ± 6.9	<.001	155.2 ± 7.6	150.8 ± 8.8	<.001	161.4 ± 6.1	159.2 ± 6.2	<.001	152.8 ± 7.5	148.9 ± 8.8	<.001
Weight	(kg)	56.9 ± 6.4	55.4 ± 9.2	<.001	55.4 ± 9.3	52.1 ± 10.9	<.001	59.1 ± 8.9	58.9 ± 9.6	<.001	55.2 ± 9.7	53.2 ± 11.3	<.001
BMI		21.8 ± 1.9	21.7 ± 2.9	<.001	22.8 ± 3.6	22.7 ± 3.9	<.001	22.6 ± 2.8	23.2 ± 3.1	<.05	23.4 ± 3.8	23.9 ± 4.2	0.13
PFT													
FVC	(l)	3.37 ± 0.79	2.54 ± 0.85	<.001	3.04 ± 0.79	2.21 ± 0.73	<.001	3.49 ± 0.76	2.78 ± 0.81	<.001	2.94 ± 0.68	2.28 ± 0.69	<.001
%VC	(%)	105.5 ± 18.7	93.1 ± 21.7	<.001	103.5 ± 18.5	90.3 ± 22.3	<.001	108.3 ± 15.5	100.2 ± 21.9	<.001	107 ± 17.3	100.4 ± 23.4	<.001
FEV1	(l)	2.09 ± 0.64	1.28 ± 0.53	<.001	1.72 ± 0.58	1.29 ± 0.51	<.001	2.67 ± 0.58	1.8 ± 0.68	<.001	2.24 ± 0.58	1.61 ± 0.52	<.001
FEV1 /FVC	(%)	64.9 ± 6.5	54.5 ± 2.3	<.001	59.8 ± 10.3	62.1 ± 13.4	<.001	79.1 ± 5.6	67.9 ± 10.2	<.001	79.6 ± 7.1	73.8 ± 13.8	<.001
Dyspnea	Unit Score												
	1	0 0.0%	1 2.1%		1 0.6%	2 1.2%		0 0.0%	1 0.8%		0 0.0%	0 0.0%	
	2	0 0.0%	4 8.3%		4 2.4%	15 8.9%		0 0.0%	2 1.7%		4 1.1%	13 3.7%	
	3	9 18.8%	24 50.0%	<.001	31 18.3%	74 43.8%	<.001	7 5.9%	20 16.8%	<.001	55 15.8%	67 19.2%	<.001
	4	30 62.5%	15 31.3%		111 65.7%	75 44.4%		75 63.0%	77 64.7%		246 70.5%	279 79.9%	
	5	9 18.8%	4 8.3%		22 13.0%	3 1.8%		37 31.1%	19 16.0%		89 25.5%	35 10.0%	
Asthma attack													
	1	0 0.0%	0 0.0%		1 0.6%	0 0.0%		0 0.0%	0 0.0%		1 0.3%	0 0.0%	
	2	0 0.0%	3 6.3%		8 4.7%	19 11.2%		1 0.8%	4 3.4%		13 3.3%	10 2.5%	
	3	7 14.6%	16 33.3%	<.001	51 30.2%	72 42.6%	<.001	11 9.2%	17 14.3%	<.001	66 16.8%	71 18.0%	0.78
	4	14 29.2%	15 31.3%		76 45.0%	51 30.2%		30 25.2%	35 29.4%		139 35.3%	139 35.3%	
	5	27 56.3%	14 29.2%		33 19.5%	27 16.0%		77 64.7%	63 52.9%		175 44.4%	174 44.2%	
Cough and phlegm													
	1	0 0.0%	1 2.1%		2 1.2%	1 0.6%		0 0.0%	0 0.0%		0 0.0%	0 0.0%	
	2	0 0.0%	3 6.3%		5 3.0%	8 4.7%		1 0.8%	2 1.7%		9 2.3%	9 2.3%	
	3	24 50.0%	34 70.8%	<.001	50 29.6%	99 58.6%	<.001	58 48.7%	66 55.5%	<.001	143 36.3%	155 39.3%	0.24
	4	23 47.9%	9 18.8%		90 53.3%	55 32.5%		58 48.7%	49 41.2%		227 57.1%	221 56.1%	
	5	1 2.1%	1 2.1%		22 13.0%	6 3.6%		2 0.8%	2 1.7%		15 3.8%	9 2.3%	

PFT: Pulmonary Function Testing

Figures are presented as mean ±SD

For peer review only

Initial evaluation versus follow-up: Mann-whitney U test

Respiratory symptoms

Numerical values are shown (%).

Initial evaluation versus follow-up: Wilcoxon signed-rank test

For peer review only

Table 4. Mean annual change in yearly spirometry results from initial evaluation to follow-up

		OS Group (n=48)	ONS Group (n=169)	NOS Group (n=119)	NONS Group (n=394)	p	post-hoc test
FEV1 decline/year	(ml)	-36.7±19.1	-26.5±18.5	-37.2±16.4	-25.5±17.2	<.001	*.**.††. t.tt
FEV1/FVC decline/year	(%)	-0.04±0.05	0.09±0.06	-0.04±0.03	-0.02±0.04	<.001	*.**.††. t.tt
%VC decline/year	(%)	-0.05±0.08	-0.05±0.08	-0.03±0.06	-0.03±0.07	<.001	† .††. t.tt

Figures are presented as mean ±SD

p-values were calculated using the Kruskal-wallis test post-hoc test: Mann-whitney U test

* : OS Group vs ONS Group

**: NOS Group vs NONS Group

†: OS Group vs NOS Group

††: ONS Group vs NONS Group

t: OS Group vs NONS Group

tt: ONS Group vs NOS Group

*,**: Current smokers / ex-smokersmeans (OS and NOS Groups) vs never smokers (ONS and NONS Groups)

†, ††: Diagnosis of obstructive ventilatory defect (OS and ONS Groups) vs no obstructive ventilatory defect (NOS and NONS Groups)

For peer review only

Table 5. Change in severity of smoking-related respiratory symptoms by comparison group at follow-up

Score	OS Group (n=48)		ONS Group (n=169)		NOS Group (n=119)		NONS Group (n=394)		p	post-hoc test
Dyspnea										
1	1	2.1%	2	1.2%	1	0.8%	0	0.0%		
2	4	8.3%	15	8.9%	2	1.7%	13	3.7%		
3	24	50.0%	74	43.8%	20	16.8%	67	19.2%	<.001	†.††. t.tt
4	15	31.3%	75	44.4%	77	64.7%	279	79.9%		
5	4	8.3%	3	1.8%	19	16.0%	35	10.0%		
Asthma attack										
1	0	0.0%	0	0.0%	0	0.0%	0	0.0%		
2	3	6.3%	19	11.2%	4	3.4%	10	2.5%		
3	16	33.3%	72	42.6%	17	14.3%	71	18.0%	<.001	*.†.††. t.tt
4	15	31.3%	51	30.2%	35	29.4%	139	35.3%		
5	14	29.2%	27	16.0%	63	52.9%	174	44.2%		
Cough and phlegm										
1	1	2.1%	1	0.6%	0	0.0%	0	0.0%		
2	3	6.3%	8	4.7%	2	1.7%	9	2.3%		
3	34	70.8%	99	58.6%	66	55.5%	155	39.3%	<.001	*.**.†.††. t.
4	9	18.8%	55	32.5%	49	41.2%	221	56.1%		
5	1	2.1%	6	3.6%	2	1.7%	9	2.3%		

Numerical values are shown (%)

p-values were calculated using the Kruskal-Wallis test post-hoc test: Mann–Whitney U test

*: OS Group vs ONS Group

**: NOS Group vs NONS Group

†: OS Group vs NOS Group

††: ONS Group vs NONS Group

t: OS Group vs NONS Group

tt :Group ONS vs NOS Group

*,**: Current smokers / ex-smokers (OS and NOS Groups) vs never smokers (ONS and NONS Groups)

†, ††: Diagnosis of obstructive ventilatory defect (OS and ONS Groups) vs no obstructive ventilatory defect (NOS and NONS Groups)

For peer review only

Acknowledgments

We thank the study participants, technical staff, administrative support team, and our coworkers for their help. This study was supported by the Environmental Restoration and Conservation Agency and Mizushima-Kyodo Hospital.

Competing interests, the authors declare that they have no competing interests.

Funding

This work was supported by the Enviromental Restoration and Conservation Agency

Competing interests

The authors declare that they have no competing interests.

Contributorship statement

HS was the principal investigator and contributed to the design of the study, administered the funding, supervised the team's work and made critical revisions to the manuscript for important intellectual content. KK designed the study, collected data, analyzed and interpreted data, and prepared the manuscript. SH took part in analyzing and interpreting the data, and made critical revisions to the manuscript for important

For peer review only

intellectual content. All other authors took part in data collection and interpretation, and have read and approved the final manuscript.

Data sharing statement:

No additional data are available.

REFERENCES

1 Yang G, Wang Y, Zeng Y, *et al.* Rapid health transition in China, 1990-2010: findings from the Global Burden of Disease Study 2010. *Lancet* 2013;381(9882):1987-2015.

2 Liu Y, Lee K, Perez-Padilla R, *et al.* Outdoor and indoor air pollution and COPD-related diseases in high- and low-income countries. *Int J Tuberc Lung Dis* 2008;12(2):115-27.

3 Yang JY, Xin JY, Ji DS, *et al.* [Variation analysis of background atmospheric pollutants in North China during the summer of 2008 to 2011]. *Huan Jing Ke Xue* 2012;33(11):3693-704.

4 Tanaka T, Asai M, Yanagita Y, *et al.* Longitudinal study of respiratory function and symptoms in a non-smoking group of long-term officially-acknowledged victims of pollution-related illness. *BMC Public Health* 2013;13:766.

5 Liu RL, Yang Y, Travers MJ, *et al.* A cross-sectional study on levels of secondhand smoke

For peer review only

in restaurants and bars in five cities in China. *Tob Control* 2011;20(6):397-402.

6 Usmanova G, Mokdad AH. Results of the Global Youth Tobacco Survey and implementation of the WHO Framework Convention on Tobacco Control in the WHO Eastern Mediterranean Region (EMR) countries. *J Epidemiol Glob Health* 2013;3(4):217-34.

7 Satomura K, Iwanaga S, Noami M*, et al.* The Framework Convention on Tobacco Control (FCTC) and Japanese anti-tobacco measures. *Tob Induc Dis* 2008;4:3.

8 Zhang J, Ou JX, Bai CX. Tobacco smoking in China: prevalence, disease burden, challenges and future strategies. *Respirology* 2011;16(8):1165-72.

9 Yang G, Fan L, Tan J*, et al.* Smoking in China: findings of the 1996 National Prevalence Survey. *JAMA* 1999;282(13):1247-53.

10 Baldwin ED CA, Richards DW Jr. Pulmonary insufficiency; physiological classification, clinical methods of analysis, standard values in normal subjects. *Medicine (Baltimore)* 1948; 27(3):243-78.

11 Yamagata T, Hirano T, Sugiura H*, et al.* Comparison of bronchodilatory properties of transdermal and inhaled long-acting beta 2-agonists. *Pulm Pharmacol Ther* 2008;21(1):160-5.

12 Curjuric I, Imboden M, Nadif R*, et al.* Different genes interact with particulate matter

For peer review only

and tobacco smoke exposure in affecting lung function decline in the general population. *PLoS One* 2012;7(7):e40175.

13 Ceylan E, Kocyigit A, Gencer M*, et al.* Increased DNA damage in patients with chronic obstructive pulmonary disease who had once smoked or been exposed to biomass. *Respir Med* 2006:1270-6.

14 Downs SH, Schindler C, Liu LJ*, et al.* Reduced exposure to PM10 and attenuated age-related decline in lung function. *N Engl J Med* 2007;357(23):2338-47.

15 Lee PN, Fry JS. Systematic review of the evidence relating FEV1 decline to giving up smoking. *BMC Med* 2010;8:84.

16 Downs SH, Schindler C, Liu LJ*, et al.* Reduced exposure to PM10 and attenuated age-related decline in lung function. *N Engl J Med* 2007;357(23):2338-47.

17 Siroux V, Pin I, Oryszczyn MP*, et al.* Relationships of active smoking to asthma and asthma severity in the EGEA study. Epidemiological study on the Genetics and Environment of Asthma. *Eur Respir J* 2000;15(3):470-7.

18 Wang Z, Chen C, Niu T*, et al.* Association of asthma with beta(2)-adrenergic receptor gene polymorphism and cigarette smoking. *Am J Respir Crit Care Med* 2001;163(6):1404-9.

19 Pelkonen M, Notkola IL, Nissinen A*, et al.* Thirty-year cumulative incidence of chronic

For peer review only

bronchitis and COPD in relation to 30-year pulmonary function and 40-year mortality: a follow-up in middle-aged rural men. *Chest* 2006;130(4):1129-37.

20 Berglund DJ, Abbey DE, Lebowitz MD*, et al.* Respiratory symptoms and pulmonary function in an elderly nonsmoking population. *Chest* 1999;115(1):49-59.

21 Oga T TM, Hajiro T, Ikeda A, *et al.* Analysis of longitudinal changes in dyspnea of patients with chronic obstructive pulmonary disease: an observational study. *Respir Res* 2012;13:85.

22 Dong GH, Zhang P, Sun B*, et al.* Long-term exposure to ambient air pollution and respiratory disease mortality in Shenyang, China: a 12-year population-based retrospective cohort study. *Respiration* 2012;84(5):360-8.

23 C. Arden Pope I caRTB, Michelle C. Turner, Aaron Cohen, Daniel Krewski, Michael Jerrett, Susan M. Gapstur, and Michael J. Thun. Lung Cancer and Cardiovascular Disease Mortality Associated with Ambient Air Pollution and Cigarette Smoke: Shape of the Exposure–Response Relationships. *Environ Health Perspect* 2011;119(11): 1616–21.

24 Kaufman G. Chronic obstructive pulmonary disease: diagnosis and management. *Nurs Stand* 2013;27(21):53-7, 60-2.

25 website.http GIfCOLDG, //www.goldcopd.org/. Global strategy for the diagnosisGlobal

For peer review only

strategy for the diagnosis. management and prevention of COPD. 2011.

For peer review only

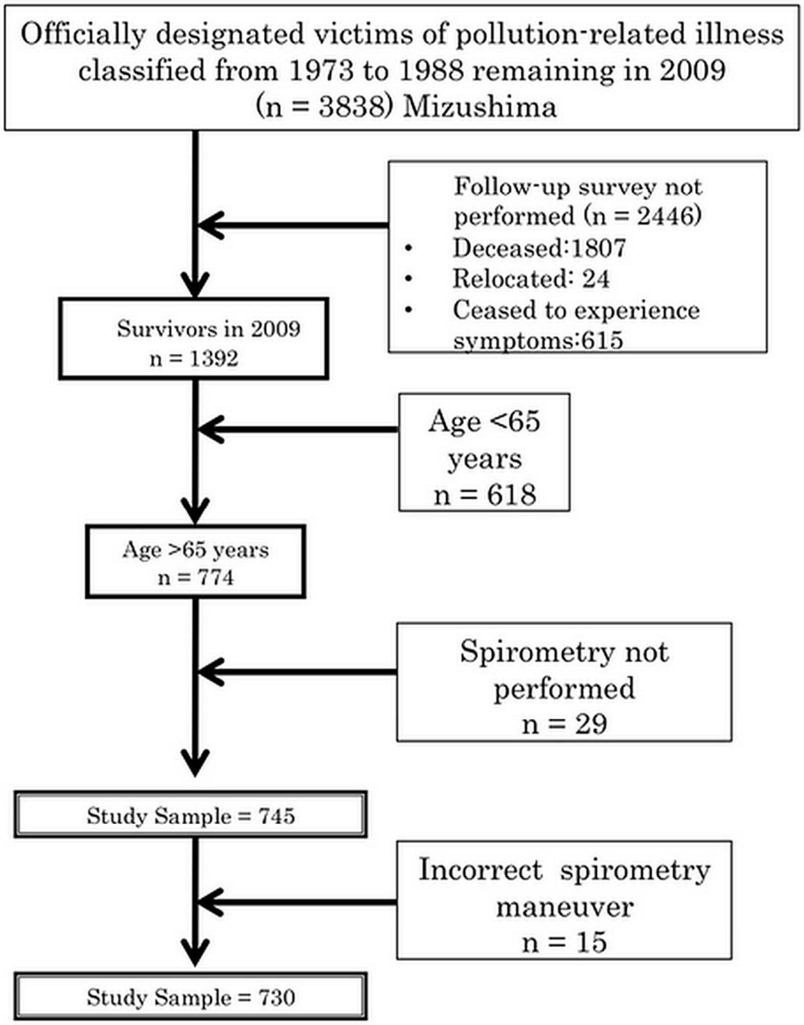

Figure 1. Flowchart showing the selection procedure for the subjects. The study included officially designated victims of pollution-related illness in Kurashiki aged 65 years or older in 2009, and for whom full data were available for statistical analysis.

STROBE 2007 (v4) Statement—Checklist of items that should be included in reports of *cohort studies*

Section/Topic	Item #	Recommendation	Reported on page #
**Title and abstract**	1	(*a*) Indicate the study's design with a commonly used term in the title or the abstract	
		(*b*) Provide in the abstract an informative and balanced summary of what was done and what was found	
**Introduction**			
Background/rationale	2	Explain the scientific background and rationale for the investigation being reported	
Objectives	3	State specific objectives, including any prespecified hypotheses	
**Methods**			
Study design	4	Present key elements of study design early in the paper	
Setting	5	Describe the setting, locations, and relevant dates, including periods of recruitment, exposure, follow-up, and data collection	
Participants	6	(*a*) Give the eligibility criteria, and the sources and methods of selection of participants. Describe methods of follow-up	
		(*b*) For matched studies, give matching criteria and number of exposed and unexposed	
Variables	7	Clearly define all outcomes, exposures, predictors, potential confounders, and effect modifiers. Give diagnostic criteria, if applicable	
Data sources/ measurement	8*	For each variable of interest, give sources of data and details of methods of assessment (measurement). Describe comparability of assessment methods if there is more than one group	
Bias	9	Describe any efforts to address potential sources of bias	
Study size	10	Explain how the study size was arrived at	
Quantitative variables	11	Explain how quantitative variables were handled in the analyses. If applicable, describe which groupings were chosen and why	
Statistical methods	12	(*a*) Describe all statistical methods, including those used to control for confounding	
		(*b*) Describe any methods used to examine subgroups and interactions	
		(*c*) Explain how missing data were addressed	
		(*d*) If applicable, explain how loss to follow-up was addressed	
		(*e*) Describe any sensitivity analyses	
**Results**			

For peer review only

Participants	13*	(a) Report numbers of individuals at each stage of study—eg numbers potentially eligible, examined for eligibility, confirmed eligible, included in the study, completing follow-up, and analysed	
		(b) Give reasons for non-participation at each stage	
		(c) Consider use of a flow diagram	
Descriptive data	14*	(a) Give characteristics of study participants (eg demographic, clinical, social) and information on exposures and potential confounders	
		(b) Indicate number of participants with missing data for each variable of interest	
		(c) Summarise follow-up time (eg, average and total amount)	
Outcome data	15*	Report numbers of outcome events or summary measures over time	
Main results	16	(*a*) Give unadjusted estimates and, if applicable, confounder-adjusted estimates and their precision (eg, 95% confidence interval). Make clear which confounders were adjusted for and why they were included	
		(*b*) Report category boundaries when continuous variables were categorized	
		(*c*) If relevant, consider translating estimates of relative risk into absolute risk for a meaningful time period	
Other analyses	17	Report other analyses done—eg analyses of subgroups and interactions, and sensitivity analyses	
**Discussion**			
Key results	18	Summarise key results with reference to study objectives	
**Limitations**			
Interpretation	20	Give a cautious overall interpretation of results considering objectives, limitations, multiplicity of analyses, results from similar studies, and other relevant evidence	
Generalisability	21	Discuss the generalisability (external validity) of the study results	
**Other information**			
Funding	22	Give the source of funding and the role of the funders for the present study and, if applicable, for the original study on which the present article is based	

*Give information separately for cases and controls in case-control studies and, if applicable, for exposed and unexposed groups in cohort and cross-sectional studies.

Note: An Explanation and Elaboration article discusses each checklist item and gives methodological background and published examples of transparent reporting. The STROBE checklist is best used in conjunction with this article (freely available on the Web sites of PLoS Medicine at http://www.plosmedicine.org/, Annals of Internal Medicine at http://www.annals.org/, and Epidemiology at http://www.epidem.com/). Information on the STROBE Initiative is available at www.strobe-statement.org.

For peer review only

BMJ Open

Tobacco use among designated air pollution victims and its impact on lung function and respiratory symptoms

Journal:	*BMJ Open*
Manuscript ID:	bmjopen-2014-005393.R1
Article Type:	Research
Date Submitted by the Author:	24-May-2014
Complete List of Authors:	kotaki, kenji; Nagasaki University Graduate School of Biomedical Sciences, Course of Cardiopulmonary Rehabilitation Science<br>Senjyu, Hideaki; Nagasaki University Graduate School of Biomedical Science, Course of Cardiopulmonary Rehabilitation Science<br>Tanaka, Takako; Nagasaki University Graduate School of Biomedical Science, Course of Cardiopulmonary Rehabilitation Science<br>Yano, Yudai; Nagasaki University Graduate School of Biomedical Science, Course of Cardiopulmonary Rehabilitation Science<br>Miyamoto, Naomi; Nagasaki University Graduate School of Biomedical Science, Course of Cardiopulmonary Rehabilitation Science<br>Nishinakagawa, Tsuyoshi; Nagasaki University Graduate School of Biomedical Science, Course of Cardiopulmonary Rehabilitation Science<br>Yanagita, Yorihide; Nagasaki University Graduate School of Biomedical Science, Course of Cardiopulmonary Rehabilitation Science<br>Asai, Masaharu; Nagasaki University Graduate School of Biomedical Science, Course of Cardiopulmonary Rehabilitation Science<br>Kozu, Ryo; Nagasaki University Hospital, Department of Rehabilitation Medicine<br>Tabusadani, Mitsuru; Nagasaki University, Center for Industry,University and Government Cooperation<br>Sawai, Terumitsu; Nagasaki University Graduate School of Biomedical Science, Course of Cardiopulmonary Rehabilitation Science<br>Honda, Sumihisa; Nagasaki University Graduate School of Biomedical Science, Course of Cardiopulmonary Rehabilitation Science
**Primary Subject Heading**:	Smoking and tobacco
Secondary Subject Heading:	Epidemiology, Respiratory medicine
Keywords:	EPIDEMIOLOGY, SOCIAL MEDICINE, Health & safety < HEALTH SERVICES ADMINISTRATION & MANAGEMENT, Public health < INFECTIOUS DISEASES, Chronic airways disease < THORACIC MEDICINE, PUBLIC HEALTH

SCHOLARONE™
Manuscripts

For peer review only - http://bmjopen.bmj.com/site/about/guidelines.xhtml

Title

Tobacco use among designated air pollution victims and its impact on lung function and respiratory symptoms

Authors

Kenji Kotaki, Hideaki Senjyu*, Takako Tanaka, Yudai Yano, Naomi Miyamoto, Tsuyoshi Nishinakagawa, Yorihide Yanagita, Masaharu Asai, Ryo Kozu, Mitsuru Tabusadani, Terumitsu Sawai, Sumihisa Honda

Author details

Department of Cardiopulmonary Rehabilitation Science, Nagasaki University Graduate School of Biomedical Sciences, Nagasaki, Japan

***Corresponding author**

Department of Cardiopulmonary Rehabilitation Science

Nagasaki University Graduate School of Biomedical Sciences

1-7-1 Sakamoto, Nagasaki 852-8520, Japan

Email: senjyu@nagasaki-u.ac.jp

Tel: +81-95-819-7963; Fax: +81-95-819-7963

Keywords: Air pollution, Tobacco, Respiratory function, Respiratory symptoms, Pulmonary function

Word count: 3685 words

For peer review only

ABSTRACT

Objectives: We sought to elucidate the long-term effects of tobacco use in designated pollution victims with and without obstructive pulmonary defects.

Design: A retrospective cross-sectional study.

Setting: The register of pollution victims in Kurashiki, Japan.

Participants: 730 individuals over 65 years of age previously diagnosed with pollution-related respiratory disease. Patients were classified into four groups according to their smoking status and whether or not they had obstructive pulmonary disease. We then compared the prevalence of respiratory symptoms and lung function over time between groups.

Primary outcome measures: Spirometry was performed and a respiratory health questionnaire completed in the same season each year for up to 30 years.

Results: Rates of smoking and respiratory disease were high in our sample. While respiratory function in non-smoking patients did not completely recover, the annual rate of change in lung function was within the normal range ($p < 0.01$). However, smokers had worse lung function and were more likely to report more severe pulmonary symptoms ($p < 0.01$).

Conclusions: Patients' respiratory function did not fully recover despite improved air quality. Our results suggest that, in the context of exposure to air pollution, tobacco use causes additional loss of lung function and exacerbates respiratory symptoms.

Article summary

Strengths and limitations

- This first study to have analysed changes in respiratory function and respiratory symptoms in a group of designated pollution victims in Japan over up to 30 years.
- Tobacco use caused additional loss of lung function and exacerbated respiratory symptoms in those who have been exposed to air pollution.
- We did not have data on the quantity of cigarettes smoked, the duration of cigarette smoking, or individual-level exposure to environmental pollutants.

For peer review only

- We were unable to account for possible confounding factors such as socio-economic class and other lifestyle factors.

For peer review only

INTRODUCTION

In many Asian nations, particularly China, air pollution is increasingly being recognised as an important emerging environmental and public health issue. Previous work has shown that levels of ambient air pollution are associated with an elevated incidence of respiratory diseases and reported prevalence of respiratory symptoms,[1] particularly asthma, pulmonary emphysema and chronic bronchitis.[2] Atmospheric pollution caused by particulate matter less than 2.5 μm in diameter PM 2.5 is reported to be particularly acute in China and in surrounding countries as a result of wind dispersal. This is a growing public health issue in the region, especially given that PM 2.5 is capable of penetrating deep into lung tissue and precipitating a number of respiratory diseases.[3]

Japan was also severely affected during its period of accelerated economic growth in the wake of post-war reconstruction in the 1960s, with both air pollution and the prevalence of respiratory diseases rising markedly. In Kurashiki, a city located in Okayama Prefecture, reports of pulmonary toxicity owing to oxidant air pollutants rose by a factor of 1.73 among the city's inhabitants in the period leading up to 1970 as yearly average SO3 levels increased. The majority of excess pollution-related deaths in this period were caused by acute exacerbation of asthma.

Although patients do not commonly recover fully from pollution-related respiratory diseases, FEV1/ FVC ratios (calculated by dividing Forced Expiratory Volume exhaled at the end of the first second of forced expiration by Forced Vital Capacity) may return to within the normal range once air quality is improved.[4] As a result, Japan passed the Pollution-Related Health Damage Compensation Law in 1973 with the aim of identifying designated air pollution victims within affected areas for compensation. Patients with a diagnosis of bronchial asthma, chronic bronchitis or pulmonary emphysema who had been resident in any designated air pollution zone for a specified period of time were legally recognised as pollution victims. These designated victims were fully reimbursed the costs of all related medical treatment by the state. However, patients' lung function, as measured

For peer review only

using FEV1/FVC ratios, was not among the diagnostic criteria used to determine eligibility for compensation, as no agreed clinical definition for Chronic Obstructive Pulmonary Disease (COPD) existed at that time.

Tobacco smoke has been shown to contain high levels of PM 2.5, and is likely to have a number of effects in common with air pollution. Tobacco use has been shown to reduce lung function, as measured using FEV1/ FVC ratios, and has been identified as a cause of COPD and other tobacco-related diseases. The World Health Organization (WHO) Framework Convention on Tobacco Control was therefore adopted in 2003.[5] However, tobacco use remains a significant public health issue. According to a WHO report, more than 1.1 billion people globally are regular smokers.[6] Furthermore, Asia continues to account for a third of total world cigarette consumption, despite local reductions in air pollution.[7]

Both air pollution and tobacco smoke have been shown to reduce patients' FEV1/FVC ratios, to increase the risk of obstructive ventilatory defects, and to exacerbate existing respiratory symptoms. Tanaka et al. have reported that when air quality is improved, the annual change in patients' FEV1 can return to within the normal range.[4] However, the effect of ongoing tobacco use on respiratory function and pulmonary symptoms in patients with already reduced FEV1/FVC ratios resulting from bronchial hypersensitivity due to air pollution has not yet been elucidated.

The purpose of the present study, carried out among a population of confirmed pollution victims living in a designated pollution-affected area, was to determine the effect of tobacco use on measures of respiratory function and prevalence of pulmonary symptoms up to 30 years after an improvement in air quality. In particular, we sought to compare these effects in patients with and without diagnosed obstructive ventilatory defects resulting from exposure to air pollution.

For peer review only

METHODS

Study design and setting

The present study was embedded in a retrospective cross-sectional analysis of lung function and respiratory symptoms in designated victims of pollution-related illness in Kurashiki between their initial evaluation (from 1976 to 1988) and follow-up in 2009. Study subjects were drawn from the register of pollution victims in Kurashiki. All study participants met the following conditions, as determined by the Public Relief System of Kurashiki City, in accordance with the Pollution-Related Health Damage Special Measures Law (1969) and the Pollution-Related Health Damage Compensation Law (1973): (1) they had resided or were employed within a designated air pollution zone, and (2) they were diagnosed with chronic bronchitis, asthma, or emphysema by a medical practitioner.

In accordance with the Public Nuisance Countermeasures Law (1967), registered victims were entitled to various forms of compensation including a monthly consultation with a doctor, yearly assessments of respiratory symptoms consisting of a detailed questionnaire and spirometry tests, and prescriptions for expectorants and bronchodilators. These patients received treatment with bronchodilators such as inhaled corticosteroids and long-acting β2 agonists, which may have caused improvements in lung function.[8] However, this study did not collect detailed data regarding treatment regimen, and we were therefore unable to evaluate the effects of treatment.

Of 419,203 total residents (204,958 males, 214,245 females), 3,838 (0.9%) were officially classified as designated pollution victims in the period up to 1988. The records of these 3,838 residents were reviewed in 2009 with the permission of the Kurashiki City Public Office (Figure 1). Of the 1,392 remaining 'officially designated victims of pollution-related illness', we screened 774 individuals aged ≥ 65 years for inclusion in the study, to focus the study on elderly people. The elderly people

For peer review only

have a higher risk factor for the onset of COPD and Show more respiratory symptoms of COPD are remarkable, as we was restricted to the patients over 65 years.After excluding 44 records that did not have complete spirometry data, the resulting sample size was 730.

We classified patients into two groups according to whether they had an obstructive impairment using the FEV1/FVC ratios recorded at their initial evaluation. Participants in each group were then further sub-divided according to whether they had ever smoked or not(current smoker or ex-smoker or ever smoked) – determined using self-reporting at follow-up, but we could not identify smoking period. This resulted in four comparison groups, labelled as Group A (n=48, current smokers and ex-smokers diagnosed with an obstructive impairment reporting), Group B (n=169, non-smokers diagnosed with an obstructive impairment), Group C (n=119, smokers and ex-smokers without a diagnosed obstructive impairment) and Group D (n=394, non-smokers without an obstructive impairment). The majority of these patients were diagnosed with chronic bronchitis (n = 528, 68.2%), asthma (n = 242, 31.3%), or emphysema (n = 4, 0.5%), as described in case reports provided by authorized medical practitioners.

The study protocol was approved by the Ethical Committee of the Nagasaki University Graduate School of Biomedical Sciences.

Clinical measurements

Patients' gender, height, weight, Body Mass Index (BMI), FVC, Vital Capacity (%VC), FEV1, FEV1/ FVC ratios, diagnosed respiratory conditions and respiratory symptoms were all recorded at baseline and at follow-up.

Spirometry measurements

Spirometry was performed by trained staff at the Mizushima-Kyodo Hospital using an electronic

For peer review only

spirometry device (FUDAC 70, Fukuda Sangyo Inc., Chiba, Japan). Tests were performed on patients in the sitting position, and were repeated until at least three reproducible forced expiratory curves had been obtained. Both FEV1 and FVC were measured and used to calculate FEV1/FVC ratios in addition to %VC using the method proposed by Baldwin et al. [9]

Annual change

The mean annual change in spirometry data was calculated by subtracting the follow-up results from the initial evaluation results and dividing by the number of years between initial evaluation and follow-up.

Respiratory symptoms

Respiratory symptoms (including dyspnoea, asthma attacks, cough, and sputum production) were assessed by physicians in the same season each year using a respiratory health questionnaire designed by the government of Japan. Each symptom was graded using a standardised five-point scale.

Dyspnoea

1. Too breathless to leave the house, or breathless when dressing or undressing.

2. Breathless after walking about 50 m or after a few minutes on level ground.

3. Breathless when walking on level ground and keeping up with people of the same age, but not breathless when walking at own pace.

4. Breathless when walking up a slight hill or the stairs.

5. Breathless only during strenuous exercise.

Wheeze

1. Severe episode ≥ 10 days each month during the last year.

For peer review only

2. Severe episode ≥ 5 days each month during the last year, or mild episode ≥ 10 days each month during the last year.

3. Severe episode ≥ 1 day each month during the last year, or mild episode ≥ 5 days each month during the last year.

4. Mild episode ≥ 1 day each month during the last year.

5. No episodes of wheezing.

Cough and sputum

1. Daily cough and sputum, with a large amount of sputum or difficulty clearing sputum.

2. Daily cough and sputum, with a moderate amount of sputum or difficulty clearing sputum.

3. Daily cough and sputum, but not troublesome during daily life.

4. Daily cough and sputum for ≤ 3 months each year.

5. No cough or sputum.

Air pollution monitoring

Data on mean daily concentrations of air pollutants were obtained at 21 points in Kurashiki. Monitoring of SO2 concentrations started in 1965, while monitoring of nitrogen dioxide (NO2) concentrations commenced in 1971.

Statistical analysis

Data from patients in each of the four comparison groups were compared using the Kruskal-Wallis test and the Mann-Whitney U test as a post-hoc test. Differences in gender distributions and the prevalence of respiratory diagnoses in each group were analysed using the Cochran-Armitage test, using the chi-square test to test for statistical significance. Changes in lung function between initial evaluation and follow-up were analysed using the Wilcoxon signed-rank test and the Mann-Whitney U test as a post-hoc test. A normality test was performed on all continuous variables using the

For peer review only

Kolmogorov-Smirnov test before analysis. All analyses were performed using PASW Statistics, version 18. Finally, p-values of <0.05 were considered statistically significant.

RESULTS

Air pollutants

Figure 2 shows the annual mean daily levels of SO_2 and NO_2 recorded from 1965 to 2009 in Kurashiki. The Air Pollution Control Law was enacted in 1968. SO_2 levels were above the acceptable level every year from 1968 to 1974 and then decreased to below 40 parts per billion (ppb), which is the acceptable level defined by the Air Pollution Control Law. In 1973, the acceptable NO_2 level was changed by the Air Pollution Control Law from 20 ppb to 40 ppb. The NO_2 level exceeded the revised acceptable level only in 1973.

Sample characteristics

Table 1 shows the characteristics of our patient sample at initial evaluation. We found significant differences ($p<0.001$) in the age distributions between groups (the mean ages were 54.3±8.3, 49.6±8.2, 49.8±7.8 and 51.3±8.9 in the Group A–D respectively). Males outnumbered females in the Group A and Group C ($p<0.001$). Patients in the Group B and Group D had significantly lower heights and weights ($p<0.001$).

We found differences in FVC values between groups, with patients in the Group B and Group D presenting significantly lower values than those in the Group A and Group C (3.04±0.79 and 2.94±0.68 versus 3.37±0.79 and 3.49±0.76, $p<0.001$). FEV_1 values, which also differed significantly between comparison groups ($p<0.001$), were lowest in the Group B (1.72±0.58ml), followed by in the Group A (2.09±0.64ml). %VC and FEV_1/FVC ratios were lowest the Group A and Group B.

For peer review only

Finally, the prevalence of bronchial asthma at initial evaluation (57.9%) was much higher in the Group B compared with all other groups ($p<0.001$).

Change in respiratory function and symptoms between initial evaluation and follow-up

Table 2 and 3 show the results of our evaluation of the change in severity of respiratory symptoms and respiratory function between initial evaluation and follow-up. Respiratory function had deteriorated in almost all subjects by follow-up. In particular, FEV/FVC ratios in Group C, which averaged 79.1±5.6% at baseline, had fallen to 67.9±10.2% at follow-up because of the development of obstructive ventilatory defects in a number of subjects. Conversely, average FEV/FVC ratios increased from 59.8±10.3% to 62.1±13.4% in Group B ($p<0.001$). The median level of respiratory symptoms worsened from level II to level III in all groups. However, Group D saw a decrease in the proportion of patients reporting asthma attacks ($p=0.78$), cough, and sputum production ($p=0.24$) over the period studied.

Mean annual change in respiratory function

Table 4 shows the mean annual change in respiratory function in each comparison group.

Mean FEV1 decreased to a similar extent in both Group A (-36.7±19.1ml/year) and Group C (-37.2± 16.4ml/year). While there was a slight fall in FEV1 in Group D (-25.5 ±17.2 ml/year), there was a marginal improvement in FEV1 (26.5±18.5ml/year) in Group B ($p<0.001$). At the same time, while FEV1/FVC ratios fell ($p<0.001$) in both Group A (-0.04±0.05) and Group C (-0.04±0.03), an improvement was seen in Group B (0.09±0.06%). Finally, %VC fell in all groups ($p<0.001$), although the decline was greater in Group A (-0.05±0.08) and Group B (-0.05±0.08) than in Group C (-0.03±0.06) and Group D (-0.03±0.07), as shown in Table 4.

For peer review only

Comparison of respiratory symptoms at final evaluation

Table 5 shows the severity of respiratory symptoms in our four comparison groups at follow-up.

In Group A and Group B, whose subjects were diagnosed with obstructive ventilatory defects at baseline, the great majority reported severe dyspnoea, asthma, cough and sputum production. Severe dyspnoea was also highly prevalent in Group A and Group B. While severity of asthma was high in both Group B and Group D at initial evaluation, the proportion of patients reporting severe asthma had only increased significantly in Group A and Group C at follow-up ($p<0.001$). Severity of asthma was similar in Group C and Group D at follow-up. Prevalence of severe cough and sputum production increased significantly in Group A and Group C ($p<0.001$), with 80% of subjects in Group A reporting scores that changed from I to III due to a worsening of symptoms.

DISCUSSION

This retrospective cross-sectional study analysed changes in respiratory function and respiratory symptoms between two points in time (initial evaluation and follow-up) among a group of designated pollution victims in Japan. Our results show for the first time that improvements in respiratory function and reductions in respiratory symptoms are prevented by continuing tobacco use.

Among those patients diagnosed with an obstructive impairment due to air pollution exposure (Groups A and B), we found that FEV1/FVC ratios had deteriorated by follow-up with no reduction in morbidity. However, our results showed an improvement in FEV1/FVC ratios in Group B, which could be attributed to the high proportion of subjects in this group diagnosed with bronchial asthma at initial evaluation (57.9%) when compared with the other groups.

For peer review only

We observed that respiratory function had deteriorated among patients in Group A despite the improvement in local air quality, resulting in depressed FEV1/FVC ratios (54.5±12.3%). Furthermore, while FEV1/FVC ratios in Group C were relatively low at the time of initial diagnosis, continuing tobacco use had resulted in some cases in obstructive impairment, causing FEV1/FVC ratios at follow-up to decrease to 67.9±10.2%.

While air pollution can cause airway obstruction resulting in asthma, reactive airway dysfunction syndrome and reduced lung function, tobacco use, which can result in airway hyper-responsiveness, has been shown to exert a stronger influence on respiratory health in a number of studies.[10 11] These effects were evident in Group A and Group C, which showed similar annual rates of change in respiratory function over time. Conversely, Downs et al. have shown that, in non-smokers, annual rates of change in FEV1 can return to within the normal range in response to an improvement in air quality.[12] These findings are reflected in the results of the present study. Previous studies have reported a mean annual rate of change in FEV1 of between -30 and -22 ml/year in healthy, non-smoking males and females aged > 65 years.[13] Patients with COPD had a mean annual change in FEV1 of 30–80 ml/year.[14] However, exposure to air pollution and tobacco smoke, both of which can result in tracheobronchial injury, can worsen annual rates of change in lung function markedly.

A high proportion of our subjects, particularly those with obstructive impairment, reported respiratory symptoms at baseline. These symptoms were more pronounced among the smokers in our sample. In Group D, however, the proportion of patients reporting asthma attacks, sputum production and cough had not increased, suggesting that these symptoms may be prevented by smoking cessation. The proportion of subjects in Group B reporting asthma attacks had increased by follow-up. Furthermore, the proportion of subjects reporting asthma attacks in Group C had increased to the same levels as that found in Group D at initial evaluation. Repeated bronchial inflammation caused by smoking and pollutant exposure can result in thickening of the bronchial

For peer review only

wall and airway remodelling that cannot be reversed even by steroid treatment following the onset of hypersensitivity.[15] Furthermore, Siroux et al. have shown that continued tobacco use can significantly increase the incidence of asthma attacks.[16] Because of this association, smoking is listed as a potential trigger for asthma and COPD in asthma management guidelines.[17] Tobacco use is also likely to have been the cause of the higher prevalence of severe cough and sputum production found in in Group A and Group C.

A number of studies have shown that smoking can cause respiratory tract inflammation and exacerbate symptoms such as cough and sputum production.[18]This was reflected in our finding that the severity of dyspnoea among subjects in Group A and Group B had increased markedly at follow-up. While a number of causes of dyspnoea have been identified, including age, poor physical fitness, muscle atrophy and social and environmental factors, we could not identify the primary determinants of dyspnoea in the present study.

[19] Finally, progression of respiratory disease and a decline in lung function can further aggravate these symptoms.[20]

In Asia, increased mortality and morbidity resulting from lung cancer, COPD, asthma and heart disorders caused by rising atmospheric PM 2.5 concentrations is a growing public health concern. According to the Organization for Economic Cooperation and Development, air pollution is expected to become the leading cause of death worldwide by 2050, and is likely to be responsible for between three and six million deaths annually.[21] Furthermore, Pope et al. have reported that worldwide pollution-related deaths could rise by 6% for each additional 10 μg/m3 increase in atmospheric PM 2.5 concentrations.[22]

The present study emphasizes the need to prevent or reduce air pollution and to improve public education on the negative health effects of smoking to reduce pulmonary morbidity on the population level. This is especially the case among designated victims, for whom additional

For peer review only

education programs, behavioural interventions and support for smoking cessation would be particularly beneficial. One such behavioural intervention that has been proven to be cost-effective in reducing smoking prevalence is the five-step Global Initiative for Obstructive Lung Disease (GOLD) framework (ask, advise, assess, assist, arrange).[23] This strategy, which aims at delaying the progress of COPD through continuing support for smoking cessation, may be applicable for use in similarly affected communities.[24]

This study has several limitations. First, information on number of cigarettes smoked and individual-level exposure to environmental pollutants was not available. Second, it was not within the scope of the present study to explore the role of gender-specific differences in exposure to tobacco smoke and pollutants – much of which may have been occupational. Third, we were unable to clearly distinguish between the effects of mainstream and side-stream cigarette smoke when considering the health effects of air pollution. Finally, we were unable to account for possible confounding factors such as socio-economic class and lifestyle factors, which may have resulted in individual-level differences in exposure to environmental pollutants.

Further work is needed to inform future prevention and treatment programs to improve the quality-of-life of the remaining designated pollution victims in Japan. The results of the present study are of particular importance in the context of high rates of smoking and worsening air quality in a number of Asian countries as they undergo rapid economic change.

For peer review only

Table 1. Demographics and respiratory characteristics of comparison groups at initial evaluation for designation of air pollution victim status

		Group A (n=48)	Group B (n=169)	Group C (n=119)	Group D (n=394)	p	post-hoc test
Age		54.3 ± 8.3	49.6 ± 8.2	49.8 ± 7.8	51.3 ± 8.9	<.001	b.c.f
Gender (male/female)		35/13	66/103	81/38	90/304	<.001	a.b.c.d.e.f
Height	(cm)	161.7 ± 6.3	155.2 ± 7.6	161.4 ± 6.1	152.8 ± 7.5	<.001	a.b.d.e.f
Weight	(kg)	56.9 ± 6.4	55.4 ± 9.3	59.1 ± 8.9	55.2 ± 9.7	<.001	b.f
BMI		21.8 ± 1.9	22.8 ± 3.6	22.6 ± 2.8	23.4 ± 3.8	<.001	a.b.d.e.
FVC	(l)	3.37 ± 0.79	3.04 ± 0.79	3.49 ± 0.76	2.94 ± 0.68	<.001	a.b.e.f
%VC	(%)	105.5 ± 18.7	103.5 ± 18.5	108.3 ± 15.5	107.6 ± 17.3	<.05	d.f
FEV1	(l)	2.09 ± 0.64	1.72 ± 0.58	2.67 ± 0.58	2.24 ± 0.58	<.001	a.b.c.d.f
FEV1/FVC	(%)	64.9 ± 6.5	59.8 ± 10.3	79.1 ± 5.6	79.6 ± 7.1	<.001	a.c.d.e.f
Data of duration after certification	(yr)	25.9±4.1	26.9±4.4	24.8±4.2	25.5±4.6	<.001	d.f
Diagnosed pulmonary disease CB,BA,PE CB+BA CB+PE, BA+PE	no	32/10/1/5/ 0/ 0	52/98/3/11/3/2	98/14/0/7/ 0/ 0	249/99/0/46/0/0	<.001	a.c.d.f
	Unit Score						
Dyspnea	1	0 (0.0%)	1(0.6%)	0 (0.0%)	0 (0.0%)		
	2	0 (0.0%)	4 (2.4%)	0 (0.0%)	4 (1.1%)		
	3	9 (18.8%)	31 (18.3%)	7 (5.9%)	55 (15.8%)	<.001	b.c.d.f
	4	30 (62.5%)	111 (65.7%)	75 (63.0%)	246 (70.5%)		
	5	9 (18.8%)	22 (13.0%)	37 (31.1%)	89 (25.5%)		
Asthma attack	1	0 (0.0%)	1 (0.6%)	0 (0.0%)	1 (0.3%)		
	2	0 (0.0%)	8 (4.7%)	1 (0.8%)	13 (3.3%)		
	3	7 (14.6%)	51 (30.2%)	11 (9.2%)	66 (16.8%)	<.001	a.b.d.f
	4	14 (29.2%)	76 (45.0%)	30 (25.2%)	139 (35.3%)		
	5	27 (56.3%)	33 (19.5%)	77 (64.7%)	175 (44.4%)		
Cough and phlegm	1	0 (0.0%)	2 (1.2%)	0 (0.0%)	0 (0.0%)		
	2	0 (0.0%)	5 (3.0%)	1 (0.8%)	9 (2.3%)		
	3	24 (50.0%)	50 (29.6%)	58 (48.7%)	143 (36.3%)	<.05	a.b.d.f
	4	23 (47.9%)	90 (53.3%)	58 (48.7%)	225 (57.1%)		
	5	1 (2.1%)	22 (13.0%)	1 (0.8%)	15 (3.8%)		

For peer review only

Figures are presented as mean ±SD

FEV1: Forced Expiratory Volume in 1 second, FVC: Forced Vital Capacity, VC%: % Vital Capacity

FEV1/FVC (%) Forced Expiratory Volume in 1 second.

CB: Chronic Bronchitis BA: Bronchial Asthma PE: Pulmonary Emphysema

CB+BA CB+PE BA+PE

Differences in sample characteristics analysed using the Kruskal-Wallis test with Cochran-Armitage tests for significance

post-hoc tests: Mann-Whitney U test, chi-square test for significance

a: Group A vs Group B

b:Group C vs Group D

c: Group A vs Group C

d: Group B vs Group D

e: Group A vs Group D

f:Group B vs Group C

a, b: Current smokers / ex-smokers (A and C Groups) vs never smokers (B and D Groups)

c, d: Diagnosis of obstructive ventilatory defect (A and B Groups) vs no obstructive ventilatory defect (C and D Groups)

For peer review only

Table 2. Change in demographic characteristics and respiratory function of designated air pollution victims by comparison group between initial evaluation and follow-up

		Initial evaluation	Follow-up	p	Initial evaluation	Follow-up	p	Initial evaluation	Follow-up	p	Initial evaluation	Follow-up	p
		Group A			Group B			Group C			Group D		
Age		54.3 ± 8.3	78.6 ± 6.5	<.001	49.6 ± 8.2	77.3 ± 7.5	<.001	49.8 ± 7.8	74.2 ± 7.1	<.001	51.3 ± 8.9	76.5 ± 7.8	<.001
Height	(cm)	161.7 ± 6.3	159.3 ± 6.9	<.001	155.2 ± 7.6	150.8 ± 8.8	<.001	161.4 ± 6.1	159.2 ± 6.2	<.001	152.8 ± 7.5	148.9 ± 8.8	<.001
Weight	(kg)	56.9 ± 6.4	55.4 ± 9.2	<.001	55.4 ± 9.3	52.1 ± 10.9	<.001	59.1 ± 8.9	58.9 ± 9.6	<.001	55.2 ± 9.7	53.2 ± 11.3	<.001
BMI		21.8 ± 1.9	21.7 ± 2.9	<.001	22.8 ± 3.6	22.7 ± 3.9	<.001	22.6 ± 2.8	23.2 ± 3.1	<.05	23.4 ± 3.8	23.9 ± 4.2	0.13
PFT													
FVC	(l)	3.37 ± 0.79	2.54 ± 0.85	<.001	3.04 ± 0.79	2.21 ± 0.73	<.001	3.49 ± 0.76	2.78 ± 0.81	<.001	2.94 ± 0.68	2.28 ± 0.69	<.001
%VC	(%)	105.5 ± 18.7	93.1 ± 21.7	<.001	103.5 ± 18.5	90.3 ± 22.3	<.001	108.3 ± 15.5	100.2 ± 21.9	<.001	107 ± 17.3	100.4 ± 23.4	<.001
FEV1	(l)	2.09 ± 0.64	1.28 ± 0.53	<.001	1.72 ± 0.58	1.29 ± 0.51	<.001	2.67 ± 0.58	1.8 ± 0.68	<.001	2.24 ± 0.58	1.61 ± 0.52	<.001
FEV1 /FVC	(%)	64.9 ± 6.5	54.5 ± 2.3	<.001	59.8 ± 10.3	62.1 ± 13.4	<.001	79.1 ± 5.6	67.9 ± 10.2	<.001	79.6 ± 7.1	73.8 ± 13.8	<.001

PFT: Pulmonary Function Testing

Figures are presented as mean ±SD

Initial evaluation versus follow-up: Mann-Whitney U test

For peer review only

Table 3. Change in respiratory symptoms of designated air pollution victims by comparison group between initial evaluation and follow-up

Unit Score	Initial evaluation		Follow-up		p	Initial evaluation		Follow-up		p	Initial evaluation		Follow-up		p	Initial evaluation		Follow-up		p
Dyspnoea	Group A					Group B					Group C					Group D				
1	0	0.0%	1	2.1%		1	0.6%	2	1.2%		0	0.0%	1	0.8%		0	0.0%	0	0.0%	
2	0	0.0%	4	8.3%		4	2.4%	15	8.9%		0	0.0%	2	1.7%		4	1.1%	13	3.7%	
3	9	18.8%	24	50.0%	<.001	31	18.3%	74	43.8%	<.001	7	5.9%	20	16.8%	<.001	55	15.8%	67	19.2%	<.001
4	30	62.5%	15	31.3%		111	65.7%	75	44.4%		75	63.0%	77	64.7%		246	70.5%	279	79.9%	
5	9	18.8%	4	8.3%		22	13.0%	3	1.8%		37	31.1%	19	16.0%		89	25.5%	35	10.0%	
Asthma attack																				
1	0	0.0%	0	0.0%		1	0.6%	0	0.0%		0	0.0%	0	0.0%		1	0.3%	0	0.0%	
2	0	0.0%	3	6.3%		8	4.7%	19	11.2%		1	0.8%	4	3.4%		13	3.3%	10	2.5%	
3	7	14.6%	16	33.3%	<.001	51	30.2%	72	42.6%	<.001	11	9.2%	17	14.3%	<.001	66	16.8%	71	18.0%	0.78
4	14	29.2%	15	31.3%		76	45.0%	51	30.2%		30	25.2%	35	29.4%		139	35.3%	139	35.3%	
5	27	56.3%	14	29.2%		33	19.5%	27	16.0%		77	64.7%	63	52.9%		175	44.4%	174	44.2%	
Cough and phlegm																				
1	0	0.0%	1	2.1%		2	1.2%	1	0.6%		0	0.0%	0	0.0%		0	0.0%	0	0.0%	
2	0	0.0%	3	6.3%		5	3.0%	8	4.7%		1	0.8%	2	1.7%		9	2.3%	9	2.3%	
3	24	50.0%	34	70.8%	<.001	50	29.6%	99	58.6%	<.001	58	48.7%	66	55.5%	<.001	143	36.3%	155	39.3%	0.24
4	23	47.9%	9	18.8%		90	53.3%	55	32.5%		58	48.7%	49	41.2%		227	57.1%	221	56.1%	
5	1	2.1%	1	2.1%		22	13.0%	6	3.6%		2	0.8%	2	1.7%		15	3.8%	9	2.3%	

Respiratory symptoms

Numerical values are shown (%).

Initial evaluation versus follow-up: Wilcoxon signed-rank test

For peer review only

Table 4. Mean annual change in yearly spirometry results from initial evaluation to follow-up by comparison group

		Group A (n=48)	Group B (n=169)	Group C (n=119)	Group D (n=394)	p	post-hoc test
FEV1 decline/year	(ml)	-36.7±19.1	-26.5±18.5	-37.2±16.4	-25.5±17.2	<.001	a.b.d.e.f
FEV1/FVC decline/year	(%)	-0.04±0.05	0.09±0.06	-0.04±0.03	-0.02±0.04	<.001	a.b.d.e.f
FVC decline/year	(ml)	-31.7±24.5	-30.1±20.7	-29.9±22.8	-25.3±17.1	<.05	d
%VC decline/year	(%)	-0.05±0.08	-0.05±0.08	-0.03±0.06	-0.03±0.07	<.001	c.d.e.f

Figures are presented as mean ±SD

p-values were calculated using the Kruskal-Wallis test post-hoc test: Mann-Whitney U test

a: Group A vs Group B

b:Group C vs Group D

c: Group A vs Group C

d: Group B vs Group D

e: Group A vs Group D

f:Group B vs Group C

a, b: Current smokers / ex-smokers (A and C Groups) vs never smokers (B and D Groups)

c, d: Diagnosis of obstructive ventilatory defect (A and B Groups) vs no obstructive ventilatory defect (C and D Groups)

For peer review only

Table 5. Change in severity of smoking-related respiratory symptoms by comparison group at follow-up

Score	Group A (n=48)		Group B (n=169)		Group C (n=119)		Group D (n=394)		p	post-hoc test
Dyspnea										
1	1	2.1%	2	1.2%	1	0.8%	0	0.0%		
2	4	8.3%	15	8.9%	2	1.7%	13	3.7%		
3	24	50.0%	74	43.8%	20	16.8%	67	19.2%	<.001	c.d.e.f
4	15	31.3%	75	44.4%	77	64.7%	279	79.9%		
5	4	8.3%	3	1.8%	19	16.0%	35	10.0%		
Asthma attack										
1	0	0.0%	0	0.0%	0	0.0%	0	0.0%		
2	3	6.3%	19	11.2%	4	3.4%	10	2.5%		
3	16	33.3%	72	42.6%	17	14.3%	71	18.0%	<.001	a.c.d.e.f
4	15	31.3%	51	30.2%	35	29.4%	139	35.3%		
5	14	29.2%	27	16.0%	63	52.9%	174	44.2%		
Cough and phlegm										
1	1	2.1%	1	0.6%	0	0.0%	0	0.0%		
2	3	6.3%	8	4.7%	2	1.7%	9	2.3%		
3	34	70.8%	99	58.6%	66	55.5%	155	39.3%	<.001	a.b.c.d.e.
4	9	18.8%	55	32.5%	49	41.2%	221	56.1%		
5	1	2.1%	6	3.6%	2	1.7%	9	2.3%		

Numerical values are shown (%)

p-values were calculated using the Kruskal-Wallis test post-hoc test: Mann–Whitney U test

a: Group A vs Group B

b:Group C vs Group D

c: Group A vs Group C

d: Group B vs Group D

e: Group A vs Group D

f:Group B vs Group C

a, b: Current smokers / ex-smokers (A and C Groups) vs never smokers (B and D Groups)

c, d: Diagnosis of obstructive ventilatory defect (A and B Groups) vs no obstructive ventilatory defect (C and D Groups)

For peer review only

Acknowledgements

We thank the study participants, technical staff, administrative support team, and our co-workers for their help. We thank the Environmental Restoration and Conservation Agency and Mizushima-Kyodo Hospital for their support.

Funding

This work was supported by the Environmental Restoration and Conservation Agency and Mizushima-Kyodo Hospital.

Competing interests

The authors declare that they have no competing interests.

Contributions

HS was the principal investigator and contributed to the design of the study, administered the funding, supervised the team's work and made critical revisions to the manuscript for intellectual content. KK designed the study, collected data, analysed and interpreted data, and prepared the manuscript. SH took part in analysing and interpreting the data, and made critical revisions to the manuscript for intellectual content. All other authors took part in data collection and interpretation, and have read and approved the final manuscript.

Data sharing statement:

Extra data can be accessed via the Dryad data repository at http://datadryad.org/ with the doi:10.5061/dryad.17tq7

For peer review only

REFERENCES

1. Yang G, Wang Y, Zeng Y, et al. Rapid health transition in China, 1990-2010: findings from the Global Burden of Disease Study 2010. Lancet 2013;**381**(9882):1987-2015 doi: 10.1016/S0140-6736(13)61097-1
S0140-6736(13)61097-1 [pii][published Online First: Epub Date]|.
2. Liu Y, Lee K, Perez-Padilla R, et al. Outdoor and indoor air pollution and COPD-related diseases in high- and low-income countries. Int J Tuberc Lung Dis 2008;**12**(2):115-27
3. Yang JY, Xin JY, Ji DS, et al. [Variation analysis of background atmospheric pollutants in North China during the summer of 2008 to 2011]. Huan Jing Ke Xue 2012;**33**(11):3693-704
4. Tanaka T, Asai M, Yanagita Y, et al. Longitudinal study of respiratory function and symptoms in a non-smoking group of long-term officially-acknowledged victims of pollution-related illness. BMC Public Health 2013;**13**:766 doi: 10.1186/1471-2458-13-766
1471-2458-13-766 [pii][published Online First: Epub Date]|.
5. Usmanova G, Mokdad AH. Results of the Global Youth Tobacco Survey and implementation of the WHO Framework Convention on Tobacco Control in the WHO Eastern Mediterranean Region (EMR) countries. J Epidemiol Glob Health 2013;**3**(4):217-34 doi: 10.1016/j.jegh.2013.07.001
S2210-6006(13)00084-1 [pii][published Online First: Epub Date]|.
6. Yang G, Fan L, Tan J, et al. Smoking in China: findings of the 1996 National Prevalence Survey. JAMA 1999;**282**(13):1247-53 doi: joc80784 [pii][published Online First: Epub Date]|.
7. M C. WHO report on the global tobacco epidemic 2008. Geneva:World Health Organization 2008
8. Yamagata T, Hirano T, Sugiura H, et al. Comparison of bronchodilatory properties of transdermal and inhaled long-acting beta 2-agonists. Pulm Pharmacol Ther 2008;**21**(1):160-5 doi: S1094-5539(07)00047-8 [pii]
10.1016/j.pupt.2007.05.004[published Online First: Epub Date]|.
9. Baldwin ED CA, Richards DW Jr. Pulmonary insufficiency; physiological classification, clinical methods of analysis, standard values in normal subjects. Medicine (Baltimore) 1948; **27(3)**:243-78
10. Curjuric I, Imboden M, Nadif R, et al. Different genes interact with particulate matter and tobacco smoke exposure in affecting lung function decline in the general population. PLoS One 2012;**7**(7):e40175 doi: 10.1371/journal.pone.0040175
PONE-D-11-21280 [pii][published Online First: Epub Date]|.
11. Ceylan E, Kocyigit A, Gencer M, et al. Jul 2006 patent 0954-6111 (Print)
0954-6111 (Linking).
12. Downs SH, Schindler C, Liu LJ, et al. Reduced exposure to PM10 and attenuated age-related decline in lung function. N Engl J Med 2007;**357**(23):2338-47 doi: 357/23/2338 [pii]
10.1056/NEJMoa073625[published Online First: Epub Date]|.
13. Lee PN, Fry JS. Systematic review of the evidence relating FEV1 decline to giving up smoking. BMC Med 2010;**8**:84 doi: 10.1186/1741-7015-8-84
1741-7015-8-84 [pii][published Online First: Epub Date]|.
14. Fletcher C, Peto R. The natural history of chronic airflow obstruction. British medical journal 1977;**1**(6077):1645-8
15. Downs SH, Schindler C, Liu LJ, et al. Reduced exposure to PM10 and attenuated age-related decline in lung function. N Engl J Med 2007;**357**(23):2338-47 doi: 10.1056/NEJMoa073625[published Online First: Epub Date]|.
16. Siroux V, Pin I, Oryszczyn MP, et al. Relationships of active smoking to asthma and asthma severity in the EGEA study. Epidemiological study on the Genetics and Environment of Asthma. Eur Respir J 2000;**15**(3):470-7

For peer review only

17. Gruffydd-Jones K. GOLD guidelines 2011: what are the implications for primary care? Primary care respiratory journal : journal of the General Practice Airways Group 2012;**21**(4):437-41 doi: 10.4104/pcrj.2012.00058[published Online First: Epub Date]|.
18. Pelkonen M, Notkola IL, Nissinen A, et al. Thirty-year cumulative incidence of chronic bronchitis and COPD in relation to 30-year pulmonary function and 40-year mortality: a follow-up in middle-aged rural men. Chest 2006;**130**(4):1129-37 doi: 130/4/1129 [pii]
10.1378/chest.130.4.1129[published Online First: Epub Date]|.
19. Berglund DJ, Abbey DE, Lebowitz MD, et al. Respiratory symptoms and pulmonary function in an elderly nonsmoking population. Chest 1999;**115**(1):49-59
20. Oga T TM, Hajiro T, Ikeda A, Nishimura K. Analysis of longitudinal changes in dyspnea of patients with chronic obstructive pulmonary disease: an observational study. Respir Res 2012;**13**:85
21. Dong GH, Zhang P, Sun B, et al. Long-term exposure to ambient air pollution and respiratory disease mortality in Shenyang, China: a 12-year population-based retrospective cohort study. Respiration; international review of thoracic diseases 2012;**84**(5):360-8 doi: 10.1159/000332930[published Online First: Epub Date]|.
22. C. Arden Pope I caRTB, Michelle C. Turner, Aaron Cohen, Daniel Krewski, Michael Jerrett, Susan M. Gapstur, and Michael J. Thun. Lung Cancer and Cardiovascular Disease Mortality Associated with Ambient Air Pollution and Cigarette Smoke: Shape of the Exposure–Response Relationships. Environ Health Perspect 2011
119(11): 1616–21
23. Kaufman G. Chronic obstructive pulmonary disease: diagnosis and management. Nurs Stand 2013;**27**(21):53-7, 60-2
24. website.http GIfCOLDG, //www.goldcopd.org/. Global strategy for the diagnosisGlobal strategy for the diagnosis. management and prevention of COPD, 2011.

For peer review only

Title

Tobacco use among designated air pollution victims and its impact on lung function and respiratory symptoms

Authors

Kenji Kotaki, Hideaki Senjyu*, Takako Tanaka, Yudai Yano, Naomi Miyamoto, Tsuyoshi Nishinakagawa, Yorihide Yanagita, Masaharu Asai, Ryo Kozu, Mitsuru Tabusadani, Terumitsu Sawai, Sumihisa Honda

Author details

Department of Cardiopulmonary Rehabilitation Science, Nagasaki University Graduate School of Biomedical Sciences, Nagasaki, Japan

*Corresponding author

Department of Cardiopulmonary Rehabilitation Science

Nagasaki University Graduate School of Biomedical Sciences

1-7-1 Sakamoto, Nagasaki 852-8520, Japan

Email: senjyu@nagasaki-u.ac.jp

Tel: +81-95-819-7963; Fax: +81-95-819-7963

Keywords: Air pollution, Tobacco, Respiratory function, Respiratory symptoms, Pulmonary function

Word count: 3685 words

For peer review only

ABSTRACT

Objectives: We sought to elucidate the long-term effects of tobacco use in designated pollution victims with and without obstructive pulmonary defects.

Design: A retrospective cross-sectional study.

Setting: The register of pollution victims in Kurashiki, Japan.

Participants: 730 individuals over 65 years of age previously diagnosed with pollution-related respiratory disease. Patients were classified into four groups according to their smoking status and whether or not they had obstructive pulmonary disease. We then compared the prevalence of respiratory symptoms and lung function over time between groups.

Primary outcome measures: Spirometry was performed and a respiratory health questionnaire completed in the same season each year for up to 30 years.

Results: Rates of smoking and respiratory disease were high in our sample. While respiratory function in non-smoking patients did not completely recover, the annual rate of change in lung function was within the normal range ($p < 0.01$). However, smokers had worse lung function and were more likely to report more severe pulmonary symptoms ($p < 0.01$).

Conclusions: Patients' respiratory function did not fully recover despite improved air quality. Our results suggest that, in the context of exposure to air pollution, tobacco use causes additional loss of lung function and exacerbates respiratory symptoms.

Article summary

Strengths and limitations

- This first study to have analysed changes in respiratory function and respiratory symptoms in a group of designated pollution victims in Japan over up to 30 years.
- Tobacco use caused additional loss of lung function and exacerbated respiratory symptoms in those who have been exposed to air pollution.
- We did not have data on the quantity of cigarettes smoked, the duration of cigarette smoking, or individual-level exposure to environmental pollutants.

For peer review only

- We were unable to account for possible confounding factors such as socio-economic class and other lifestyle factors.

For peer review only

INTRODUCTION

In many Asian nations, particularly China, air pollution is increasingly being recognised as an important emerging environmental and public health issue. Previous work has shown that levels of ambient air pollution are associated with an elevated incidence of respiratory diseases and reported prevalence of respiratory symptoms,[1] particularly asthma, pulmonary emphysema and chronic bronchitis.[2] Atmospheric pollution caused by particulate matter less than 2.5 μm in diameter PM 2.5 is reported to be particularly acute in China and in surrounding countries as a result of wind dispersal. This is a growing public health issue in the region, especially given that PM 2.5 is capable of penetrating deep into lung tissue and precipitating a number of respiratory diseases.[3]

Japan was also severely affected during its period of accelerated economic growth in the wake of post-war reconstruction in the 1960s, with both air pollution and the prevalence of respiratory diseases rising markedly. In Kurashiki, a city located in Okayama Prefecture, reports of pulmonary toxicity owing to oxidant air pollutants rose by a factor of 1.73 among the city's inhabitants in the period leading up to 1970 as yearly average SO3 levels increased. The majority of excess pollution-related deaths in this period were caused by acute exacerbation of asthma.

Although patients do not commonly recover fully from pollution-related respiratory diseases, FEV1/ FVC ratios (calculated by dividing Forced Expiratory Volume exhaled at the end of the first second of forced expiration by Forced Vital Capacity) may return to within the normal range once air quality is improved.[4] As a result, Japan passed the Pollution-Related Health Damage Compensation Law in 1973 with the aim of identifying designated air pollution victims within affected areas for compensation. Patients with a diagnosis of bronchial asthma, chronic bronchitis or pulmonary emphysema who had been resident in any designated air pollution zone for a specified period of time were legally recognised as pollution victims. These designated victims were fully reimbursed the costs of all related medical treatment by the state. However, patients' lung function, as measured

using FEV1/FVC ratios, was not among the diagnostic criteria used to determine eligibility for compensation, as no agreed clinical definition for Chronic Obstructive Pulmonary Disease (COPD) existed at that time.

Tobacco smoke has been shown to contain high levels of PM 2.5, and is likely to have a number of effects in common with air pollution. Tobacco use has been shown to reduce lung function, as measured using FEV1/ FVC ratios, and has been identified as a cause of COPD and other tobacco-related diseases. The World Health Organization (WHO) Framework Convention on Tobacco Control was therefore adopted in 2003.[5] However, tobacco use remains a significant public health issue. According to a WHO report, more than 1.1 billion people globally are regular smokers.[6] Furthermore, Asia continues to account for a third of total world cigarette consumption, despite local reductions in air pollution.[7]

Both air pollution and tobacco smoke have been shown to reduce patients' FEV1/FVC ratios, to increase the risk of obstructive ventilatory defects, and to exacerbate existing respiratory symptoms. Tanaka et al. have reported that when air quality is improved, the annual change in patients' FEV1 can return to within the normal range.[4] However, the effect of ongoing tobacco use on respiratory function and pulmonary symptoms in patients with already reduced FEV1/FVC ratios resulting from bronchial hypersensitivity due to air pollution has not yet been elucidated.

The purpose of the present study, carried out among a population of confirmed pollution victims living in a designated pollution-affected area, was to determine the effect of tobacco use on measures of respiratory function and prevalence of pulmonary symptoms up to 30 years after an improvement in air quality. In particular, we sought to compare these effects in patients with and without diagnosed obstructive ventilatory defects resulting from exposure to air pollution.

For peer review only

METHODS

Study design and setting

The present study was embedded in a retrospective cross-sectional analysis of lung function and respiratory symptoms in designated victims of pollution-related illness in Kurashiki between their initial evaluation (from 1976 to 1988) and follow-up in 2009. Study subjects were drawn from the register of pollution victims in Kurashiki. All study participants met the following conditions, as determined by the Public Relief System of Kurashiki City, in accordance with the Pollution-Related Health Damage Special Measures Law (1969) and the Pollution-Related Health Damage Compensation Law (1973): (1) they had resided or were employed within a designated air pollution zone, and (2) they were diagnosed with chronic bronchitis, asthma, or emphysema by a medical practitioner.

In accordance with the Public Nuisance Countermeasures Law (1967), registered victims were entitled to various forms of compensation including a monthly consultation with a doctor, yearly assessments of respiratory symptoms consisting of a detailed questionnaire and spirometry tests, and prescriptions for expectorants and bronchodilators. These patients received treatment with bronchodilators such as inhaled corticosteroids and long-acting β2 agonists, which may have caused improvements in lung function.[8] However, this study did not collect detailed data regarding treatment regimen, and we were therefore unable to evaluate the effects of treatment.

Of 419,203 total residents (204,958 males, 214,245 females), 3,838 (0.9%) were officially classified as designated pollution victims in the period up to 1988. The records of these 3,838 residents were reviewed in 2009 with the permission of the Kurashiki City Public Office (Figure 1). Of the 1,392 remaining 'officially designated victims of pollution-related illness', we screened 774 individuals aged ≥ 65 years for inclusion in the study, to focus the study on elderly people. The elderly people

have a higher risk factor for the onset of COPD and Show more respiratory symptoms of COPD are remarkable, as we was restricted to the patients over 65 years.After excluding 44 records that did not have complete spirometry data, the resulting sample size was 730.

We classified patients into two groups according to whether they had an obstructive impairment using the FEV1/FVC ratios recorded at their initial evaluation. Participants in each group were then further sub-divided according to whether they had ever smoked or not(current smoker or ex-smoker or ever smoked) – determined using self-reporting at follow-up, but we could not identify smoking period. This resulted in four comparison groups, labelled as Group A (n=48, current smokers and ex-smokers diagnosed with an obstructive impairment reporting), Group B (n=169, non-smokers diagnosed with an obstructive impairment), Group C (n=119, smokers and ex-smokers without a diagnosed obstructive impairment) and Group D (n=394, non-smokers without an obstructive impairment). The majority of these patients were diagnosed with chronic bronchitis (n = 528, 68.2%), asthma (n = 242, 31.3%), or emphysema (n = 4, 0.5%), as described in case reports provided by authorized medical practitioners.

The study protocol was approved by the Ethical Committee of the Nagasaki University Graduate School of Biomedical Sciences.

Clinical measurements

Patients' gender, height, weight, Body Mass Index (BMI), FVC, Vital Capacity (%VC), FEV1, FEV1/ FVC ratios, diagnosed respiratory conditions and respiratory symptoms were all recorded at baseline and at follow-up.

Spirometry measurements

Spirometry was performed by trained staff at the Mizushima-Kyodo Hospital using an electronic

For peer review only

spirometry device (FUDAC 70, Fukuda Sangyo Inc., Chiba, Japan). Tests were performed on patients in the sitting position, and were repeated until at least three reproducible forced expiratory curves had been obtained. Both FEV1 and FVC were measured and used to calculate FEV1/FVC ratios in addition to %VC using the method proposed by Baldwin et al. [9]

Annual change

The mean annual change in spirometry data was calculated by subtracting the follow-up results from the initial evaluation results and dividing by the number of years between initial evaluation and follow-up.

Respiratory symptoms

Respiratory symptoms (including dyspnoea, asthma attacks, cough, and sputum production) were assessed by physicians in the same season each year using a respiratory health questionnaire designed by the government of Japan. Each symptom was graded using a standardised five-point scale.

Dyspnoea

1. Too breathless to leave the house, or breathless when dressing or undressing.

2. Breathless after walking about 50 m or after a few minutes on level ground.

3. Breathless when walking on level ground and keeping up with people of the same age, but not breathless when walking at own pace.

4. Breathless when walking up a slight hill or the stairs.

5. Breathless only during strenuous exercise.

Wheeze

1. Severe episode ≥ 10 days each month during the last year.

For peer review only

2. Severe episode ≥ 5 days each month during the last year, or mild episode ≥ 10 days each month during the last year.

3. Severe episode ≥ 1 day each month during the last year, or mild episode ≥ 5 days each month during the last year.

4. Mild episode ≥ 1 day each month during the last year.

5. No episodes of wheezing.

Cough and sputum

1. Daily cough and sputum, with a large amount of sputum or difficulty clearing sputum.

2. Daily cough and sputum, with a moderate amount of sputum or difficulty clearing sputum.

3. Daily cough and sputum, but not troublesome during daily life.

4. Daily cough and sputum for ≤ 3 months each year.

5. No cough or sputum.

Air pollution monitoring

Data on mean daily concentrations of air pollutants were obtained at 21 points in Kurashiki. Monitoring of SO2 concentrations started in 1965, while monitoring of nitrogen dioxide (NO2) concentrations commenced in 1971.

Statistical analysis

Data from patients in each of the four comparison groups were compared using the Kruskal-Wallis test and the Mann-Whitney U test as a post-hoc test. Differences in gender distributions and the prevalence of respiratory diagnoses in each group were analysed using the Cochran-Armitage test, using the chi-square test to test for statistical significance. Changes in lung function between initial evaluation and follow-up were analysed using the Wilcoxon signed-rank test and the Mann-Whitney U test as a post-hoc test. A normality test was performed on all continuous variables using the

For peer review only

Kolmogorov-Smirnov test before analysis. All analyses were performed using PASW Statistics, version 18. Finally, p-values of <0.05 were considered statistically significant.

RESULTS

Air pollutants

Figure 2 shows the annual mean daily levels of SO_2 and NO_2 recorded from 1965 to 2009 in Kurashiki. The Air Pollution Control Law was enacted in 1968. SO_2 levels were above the acceptable level every year from 1968 to 1974 and then decreased to below 40 parts per billion (ppb), which is the acceptable level defined by the Air Pollution Control Law. In 1973, the acceptable NO_2 level was changed by the Air Pollution Control Law from 20 ppb to 40 ppb. The NO_2 level exceeded the revised acceptable level only in 1973.

Sample characteristics

Table 1 shows the characteristics of our patient sample at initial evaluation. We found significant differences ($p<0.001$) in the age distributions between groups (the mean ages were 54.3±8.3, 49.6±8.2, 49.8±7.8 and 51.3±8.9 in the Group A–D respectively). Males outnumbered females in the Group A and Group C ($p<0.001$). Patients in the Group B and Group D had significantly lower heights and weights ($p<0.001$).

We found differences in FVC values between groups, with patients in the Group B and Group D presenting significantly lower values than those in the Group A and Group C (3.04±0.79 and 2.94±0.68 versus 3.37±0.79 and 3.49±0.76, $p<0.001$). FEV1 values, which also differed significantly between comparison groups ($p<0.001$), were lowest in the Group B (1.72±0.58ml), followed by in the Group A (2.09±0.64ml). %VC and FEV1/FVC ratios were lowest the Group A and Group B.

For peer review only

Finally, the prevalence of bronchial asthma at initial evaluation (57.9%) was much higher in the Group B compared with all other groups ($p<0.001$).

Change in respiratory function and symptoms between initial evaluation and follow-up

Table 2 and 3 show the results of our evaluation of the change in severity of respiratory symptoms and respiratory function between initial evaluation and follow-up. Respiratory function had deteriorated in almost all subjects by follow-up. In particular, FEV/FVC ratios in Group C, which averaged 79.1±5.6% at baseline, had fallen to 67.9±10.2% at follow-up because of the development of obstructive ventilatory defects in a number of subjects. Conversely, average FEV/FVC ratios increased from 59.8±10.3% to 62.1±13.4% in Group B ($p<0.001$). The median level of respiratory symptoms worsened from level II to level III in all groups. However, Group D saw a decrease in the proportion of patients reporting asthma attacks ($p=0.78$), cough, and sputum production ($p=0.24$) over the period studied.

Mean annual change in respiratory function

Table 4 shows the mean annual change in respiratory function in each comparison group.

Mean FEV1 decreased to a similar extent in both Group A (-36.7±19.1ml/year) and Group C (-37.2± 16.4ml/year). While there was a slight fall in FEV1 in Group D (-25.5 ±17.2 ml/year), there was a marginal improvement in FEV1 (26.5±18.5ml/year) in Group B ($p<0.001$). At the same time, while FEV1/FVC ratios fell ($p<0.001$) in both Group A (-0.04±0.05) and Group C (-0.04±0.03), an improvement was seen in Group B (0.09±0.06%). Finally, %VC fell in all groups ($p<0.001$), although the decline was greater in Group A (-0.05±0.08) and Group B (-0.05±0.08) than in Group C (-0.03±0.06) and Group D (-0.03±0.07), as shown in Table 4.

For peer review only

Comparison of respiratory symptoms at final evaluation

Table 5 shows the severity of respiratory symptoms in our four comparison groups at follow-up.

In Group A and Group B, whose subjects were diagnosed with obstructive ventilatory defects at baseline, the great majority reported severe dyspnoea, asthma, cough and sputum production. Severe dyspnoea was also highly prevalent in Group A and Group B. While severity of asthma was high in both Group B and Group D at initial evaluation, the proportion of patients reporting severe asthma had only increased significantly in Group A and Group C at follow-up ($p<0.001$). Severity of asthma was similar in Group C and Group D at follow-up. Prevalence of severe cough and sputum production increased significantly in Group A and Group C ($p<0.001$), with 80% of subjects in Group A reporting scores that changed from I to III due to a worsening of symptoms.

DISCUSSION

This retrospective cross-sectional study analysed changes in respiratory function and respiratory symptoms between two points in time (initial evaluation and follow-up) among a group of designated pollution victims in Japan. Our results show for the first time that improvements in respiratory function and reductions in respiratory symptoms are prevented by continuing tobacco use.

Among those patients diagnosed with an obstructive impairment due to air pollution exposure (Groups A and B), we found that FEV1/FVC ratios had deteriorated by follow-up with no reduction in morbidity. However, our results showed an improvement in FEV1/FVC ratios in Group B, which could be attributed to the high proportion of subjects in this group diagnosed with bronchial asthma at initial evaluation (57.9%) when compared with the other groups.

For peer review only

We observed that respiratory function had deteriorated among patients in Group A despite the improvement in local air quality, resulting in depressed FEV1/FVC ratios (54.5±12.3%). Furthermore, while FEV1/FVC ratios in Group C were relatively low at the time of initial diagnosis, continuing tobacco use had resulted in some cases in obstructive impairment, causing FEV1/FVC ratios at follow-up to decrease to 67.9±10.2%.

While air pollution can cause airway obstruction resulting in asthma, reactive airway dysfunction syndrome and reduced lung function, tobacco use, which can result in airway hyper-responsiveness, has been shown to exert a stronger influence on respiratory health in a number of studies.[10 11] These effects were evident in Group A and Group C, which showed similar annual rates of change in respiratory function over time. Conversely, Downs et al. have shown that, in non-smokers, annual rates of change in FEV1 can return to within the normal range in response to an improvement in air quality.[12] These findings are reflected in the results of the present study. Previous studies have reported a mean annual rate of change in FEV1 of between -30 and -22 ml/year in healthy, non-smoking males and females aged > 65 years.[13] Patients with COPD had a mean annual change in FEV1 of 30–80 ml/year.[14] However, exposure to air pollution and tobacco smoke, both of which can result in tracheobronchial injury, can worsen annual rates of change in lung function markedly.

A high proportion of our subjects, particularly those with obstructive impairment, reported respiratory symptoms at baseline. These symptoms were more pronounced among the smokers in our sample. In Group D, however, the proportion of patients reporting asthma attacks, sputum production and cough had not increased, suggesting that these symptoms may be prevented by smoking cessation. The proportion of subjects in Group B reporting asthma attacks had increased by follow-up. Furthermore, the proportion of subjects reporting asthma attacks in Group C had increased to the same levels as that found in Group D at initial evaluation. Repeated bronchial inflammation caused by smoking and pollutant exposure can result in thickening of the bronchial

For peer review only

wall and airway remodelling that cannot be reversed even by steroid treatment following the onset of hypersensitivity.[15] Furthermore, Siroux et al. have shown that continued tobacco use can significantly increase the incidence of asthma attacks.[16] Because of this association, smoking is listed as a potential trigger for asthma and COPD in asthma management guidelines.[17] Tobacco use is also likely to have been the cause of the higher prevalence of severe cough and sputum production found in in Group A and Group C.

A number of studies have shown that smoking can cause respiratory tract inflammation and exacerbate symptoms such as cough and sputum production.[18]This was reflected in our finding that the severity of dyspnoea among subjects in Group A and Group B had increased markedly at follow-up. While a number of causes of dyspnoea have been identified, including age, poor physical fitness, muscle atrophy and social and environmental factors, we could not identify the primary determinants of dyspnoea in the present study.

[19] Finally, progression of respiratory disease and a decline in lung function can further aggravate these symptoms.[20]

In Asia, increased mortality and morbidity resulting from lung cancer, COPD, asthma and heart disorders caused by rising atmospheric PM 2.5 concentrations is a growing public health concern. According to the Organization for Economic Cooperation and Development, air pollution is expected to become the leading cause of death worldwide by 2050, and is likely to be responsible for between three and six million deaths annually.[21] Furthermore, Pope et al. have reported that worldwide pollution-related deaths could rise by 6% for each additional 10 μg/m3 increase in atmospheric PM 2.5 concentrations.[22]

The present study emphasizes the need to prevent or reduce air pollution and to improve public education on the negative health effects of smoking to reduce pulmonary morbidity on the population level. This is especially the case among designated victims, for whom additional

For peer review only

education programs, behavioural interventions and support for smoking cessation would be particularly beneficial. One such behavioural intervention that has been proven to be cost-effective in reducing smoking prevalence is the five-step Global Initiative for Obstructive Lung Disease (GOLD) framework (ask, advise, assess, assist, arrange).[23] This strategy, which aims at delaying the progress of COPD through continuing support for smoking cessation, may be applicable for use in similarly affected communities.[24]

This study has several limitations. First, information on number of cigarettes smoked and individual-level exposure to environmental pollutants was not available. Second, it was not within the scope of the present study to explore the role of gender-specific differences in exposure to tobacco smoke and pollutants – much of which may have been occupational. Third, we were unable to clearly distinguish between the effects of mainstream and side-stream cigarette smoke when considering the health effects of air pollution. Finally, we were unable to account for possible confounding factors such as socio-economic class and lifestyle factors, which may have resulted in individual-level differences in exposure to environmental pollutants.

Further work is needed to inform future prevention and treatment programs to improve the quality-of-life of the remaining designated pollution victims in Japan. The results of the present study are of particular importance in the context of high rates of smoking and worsening air quality in a number of Asian countries as they undergo rapid economic change.

For peer review only

Table 1. Demographics and respiratory characteristics of comparison groups at initial evaluation for designation of air pollution victim status

		Group A (n=48)	Group B (n=169)	Group C (n=119)	Group D (n=394)	p	post-hoc test
Age		54.3 ± 8.3	49.6 ± 8.2	49.8 ± 7.8	51.3 ± 8.9	<.001	b.c.f
Gender (male/female)		35/13	66/103	81/38	90/304	<.001	a.b.c.d.e.f
Height	(cm)	161.7 ± 6.3	155.2 ± 7.6	161.4 ± 6.1	152.8 ± 7.5	<.001	a.b.d.e.f
Weight	(kg)	56.9 ± 6.4	55.4 ± 9.3	59.1 ± 8.9	55.2 ± 9.7	<.001	b.f
BMI		21.8 ± 1.9	22.8 ± 3.6	22.6 ± 2.8	23.4 ± 3.8	<.001	a.b.d.e.
FVC	(l)	3.37 ± 0.79	3.04 ± 0.79	3.49 ± 0.76	2.94 ± 0.68	<.001	a.b.e.f
%VC	(%)	105.5 ± 18.7	103.5 ± 18.5	108.3 ± 15.5	107.6 ± 17.3	<.05	d.f
FEV1	(l)	2.09 ± 0.64	1.72 ± 0.58	2.67 ± 0.58	2.24 ± 0.58	<.001	a.b.c.d.f
FEV1/FVC	(%)	64.9 ± 6.5	59.8 ± 10.3	79.1 ± 5.6	79.6 ± 7.1	<.001	a.c.d.e.f
Data of duration after certification	(yr)	25.9±4.1	26.9±4.4	24.8±4.2	25.5±4.6	<.001	d.f
Diagnosed pulmonary disease CB,BA,PE CB+BA CB+PE, BA+PE	no	32/10/1/5/ 0/ 0	52/98/3/11/3/2	98/14/0/7/ 0/ 0	249/99/0/46/0/0	<.001	a.c.d.f
	Unit Score						
Dyspnea	1	0 (0.0%)	1(0.6%)	0 (0.0%)	0 (0.0%)	<.001	b.c.d.f
	2	0 (0.0%)	4 (2.4%)	0 (0.0%)	4 (1.1%)		
	3	9 (18.8%)	31 (18.3%)	7 (5.9%)	55 (15.8%)		
	4	30 (62.5%)	111 (65.7%)	75 (63.0%)	246 (70.5%)		
	5	9 (18.8%)	22 (13.0%)	37 (31.1%)	89 (25.5%)		
Asthma attack	1	0 (0.0%)	1 (0.6%)	0 (0.0%)	1 (0.3%)	<.001	a.b.d.f
	2	0 (0.0%)	8 (4.7%)	1 (0.8%)	13 (3.3%)		
	3	7 (14.6%)	51 (30.2%)	11 (9.2%)	66 (16.8%)		
	4	14 (29.2%)	76 (45.0%)	30 (25.2%)	139 (35.3%)		
	5	27 (56.3%)	33 (19.5%)	77 (64.7%)	175 (44.4%)		
Cough and phlegm	1	0 (0.0%)	2 (1.2%)	0 (0.0%)	0 (0.0%)	<.05	a.b.d.f
	2	0 (0.0%)	5 (3.0%)	1 (0.8%)	9 (2.3%)		
	3	24 (50.0%)	50 (29.6%)	58 (48.7%)	143 (36.3%)		
	4	23 (47.9%)	90 (53.3%)	58 (48.7%)	225 (57.1%)		
	5	1 (2.1%)	22 (13.0%)	1 (0.8%)	15 (3.8%)		

For peer review only

Figures are presented as mean ±SD

FEV1: Forced Expiratory Volume in 1 second, FVC: Forced Vital Capacity, VC%: % Vital Capacity

FEV1/FVC (%) Forced Expiratory Volume in 1 second.

CB: Chronic Bronchitis BA: Bronchial Asthma PE: Pulmonary Emphysema

CB+BA CB+PE BA+PE

Differences in sample characteristics analysed using the Kruskal-Wallis test with Cochran-Armitage tests for significance

post-hoc tests: Mann-Whitney U test, chi-square test for significance

a: Group A vs Group B

b:Group C vs Group D

c: Group A vs Group C

d: Group B vs Group D

e: Group A vs Group D

f:Group B vs Group C

a, b: Current smokers / ex-smokers (A and C Groups) vs never smokers (B and D Groups)

c, d: Diagnosis of obstructive ventilatory defect (A and B Groups) vs no obstructive ventilatory defect (C and D Groups)

For peer review only

Table 2. Change in demographic characteristics and respiratory function of designated air pollution victims by comparison group between initial evaluation and follow-up

		Initial evaluation	Follow-up	p	Initial evaluation	Follow-up	p	Initial evaluation	Follow-up	p	Initial evaluation	Follow-up	p
		Group A			Group B			Group C			Group D		
Age		54.3 ± 8.3	78.6 ± 6.5	<.001	49.6 ± 8.2	77.3 ± 7.5	<.001	49.8 ± 7.8	74.2 ± 7.1	<.001	51.3 ± 8.9	76.5 ± 7.8	<.001
Height	(cm)	161.7 ± 6.3	159.3 ± 6.9	<.001	155.2 ± 7.6	150.8 ± 8.8	<.001	161.4 ± 6.1	159.2 ± 6.2	<.001	152.8 ± 7.5	148.9 ± 8.8	<.001
Weight	(kg)	56.9 ± 6.4	55.4 ± 9.2	<.001	55.4 ± 9.3	52.1 ± 10.9	<.001	59.1 ± 8.9	58.9 ± 9.6	<.001	55.2 ± 9.7	53.2 ± 11.3	<.001
BMI		21.8 ± 1.9	21.7 ± 2.9	<.001	22.8 ± 3.6	22.7 ± 3.9	<.001	22.6 ± 2.8	23.2 ± 3.1	<.05	23.4 ± 3.8	23.9 ± 4.2	0.13
PFT													
FVC	(l)	3.37 ± 0.79	2.54 ± 0.85	<.001	3.04 ± 0.79	2.21 ± 0.73	<.001	3.49 ± 0.76	2.78 ± 0.81	<.001	2.94 ± 0.68	2.28 ± 0.69	<.001
%VC	(%)	105.5 ± 18.7	93.1 ± 21.7	<.001	103.5 ± 18.5	90.3 ± 22.3	<.001	108.3 ± 15.5	100.2 ± 21.9	<.001	107 ± 17.3	100.4 ± 23.4	<.001
FEV1	(l)	2.09 ± 0.64	1.28 ± 0.53	<.001	1.72 ± 0.58	1.29 ± 0.51	<.001	2.67 ± 0.58	1.8 ± 0.68	<.001	2.24 ± 0.58	1.61 ± 0.52	<.001
FEV1 /FVC	(%)	64.9 ± 6.5	54.5 ± 2.3	<.001	59.8 ± 10.3	62.1 ± 13.4	<.001	79.1 ± 5.6	67.9 ± 10.2	<.001	79.6 ± 7.1	73.8 ± 13.8	<.001

PFT: Pulmonary Function Testing

Figures are presented as mean ±SD

Initial evaluation versus follow-up: Mann-Whitney U test

For peer review only

Table 3. Change in respiratory symptoms of designated air pollution victims by comparison group between initial evaluation and follow-up

	Unit Score	Initial evaluation		Follow-up		p	Initial evaluation		Follow-up		p	Initial evaluation		Follow-up		p	Initial evaluation		Follow-up		p
Dyspnoea		Group A					Group B					Group C					Group D				
	1	0	0.0%	1	2.1%		1	0.6%	2	1.2%		0	0.0%	1	0.8%		0	0.0%	0	0.0%	
	2	0	0.0%	4	8.3%		4	2.4%	15	8.9%		0	0.0%	2	1.7%		4	1.1%	13	3.7%	
	3	9	18.8%	24	50.0%	<.001	31	18.3%	74	43.8%	<.001	7	5.9%	20	16.8%	<.001	55	15.8%	67	19.2%	<.001
	4	30	62.5%	15	31.3%		111	65.7%	75	44.4%		75	63.0%	77	64.7%		246	70.5%	279	79.9%	
	5	9	18.8%	4	8.3%		22	13.0%	3	1.8%		37	31.1%	19	16.0%		89	25.5%	35	10.0%	
Asthma attack																					
	1	0	0.0%	0	0.0%		1	0.6%	0	0.0%		0	0.0%	0	0.0%		1	0.3%	0	0.0%	
	2	0	0.0%	3	6.3%		8	4.7%	19	11.2%		1	0.8%	4	3.4%		13	3.3%	10	2.5%	
	3	7	14.6%	16	33.3%	<.001	51	30.2%	72	42.6%	<.001	11	9.2%	17	14.3%	<.001	66	16.8%	71	18.0%	0.78
	4	14	29.2%	15	31.3%		76	45.0%	51	30.2%		30	25.2%	35	29.4%		139	35.3%	139	35.3%	
	5	27	56.3%	14	29.2%		33	19.5%	27	16.0%		77	64.7%	63	52.9%		175	44.4%	174	44.2%	
Cough and phlegm																					
	1	0	0.0%	1	2.1%		2	1.2%	1	0.6%		0	0.0%	0	0.0%		0	0.0%	0	0.0%	
	2	0	0.0%	3	6.3%		5	3.0%	8	4.7%		1	0.8%	2	1.7%		9	2.3%	9	2.3%	
	3	24	50.0%	34	70.8%	<.001	50	29.6%	99	58.6%	<.001	58	48.7%	66	55.5%	<.001	143	36.3%	155	39.3%	0.24
	4	23	47.9%	9	18.8%		90	53.3%	55	32.5%		58	48.7%	49	41.2%		227	57.1%	221	56.1%	
	5	1	2.1%	1	2.1%		22	13.0%	6	3.6%		2	0.8%	2	1.7%		15	3.8%	9	2.3%	

Respiratory symptoms

Numerical values are shown (%).

Initial evaluation versus follow-up: Wilcoxon signed-rank test

For peer review only

Table 4. Mean annual change in yearly spirometry results from initial evaluation to follow-up by comparison group

		Group A (n=48)	Group B (n=169)	Group C (n=119)	Group D (n=394)	p	post-hoc test
FEV1 decline/year	(ml)	-36.7±19.1	-26.5±18.5	-37.2±16.4	-25.5±17.2	<.001	a.b.d.e.f
FEV1/FVC decline/year	(%)	-0.04±0.05	0.09±0.06	-0.04±0.03	-0.02±0.04	<.001	a.b.d.e.f
FVC decline/year	(ml)	-31.7±24.5	-30.1±20.7	-29.9±22.8	-25.3±17.1	<.05	d
%VC decline/year	(%)	-0.05±0.08	-0.05±0.08	-0.03±0.06	-0.03±0.07	<.001	c.d.e.f

Figures are presented as mean ±SD

p-values were calculated using the Kruskal-Wallis test post-hoc test: Mann-Whitney U test

a: Group A vs Group B

b:Group C vs Group D

c: Group A vs Group C

d: Group B vs Group D

e: Group A vs Group D

f:Group B vs Group C

a, b: Current smokers / ex-smokers (A and C Groups) vs never smokers (B and D Groups)

c, d: Diagnosis of obstructive ventilatory defect (A and B Groups) vs no obstructive ventilatory defect (C and D Groups)

For peer review only

Table 5. Change in severity of smoking-related respiratory symptoms by comparison group at follow-up

Score	Group A (n=48)		Group B (n=169)		Group C (n=119)		Group D (n=394)		p	post-hoc test
Dyspnea										
1	1	2.1%	2	1.2%	1	0.8%	0	0.0%		
2	4	8.3%	15	8.9%	2	1.7%	13	3.7%		
3	24	50.0%	74	43.8%	20	16.8%	67	19.2%	<.001	c.d.e.f
4	15	31.3%	75	44.4%	77	64.7%	279	79.9%		
5	4	8.3%	3	1.8%	19	16.0%	35	10.0%		
Asthma attack										
1	0	0.0%	0	0.0%	0	0.0%	0	0.0%		
2	3	6.3%	19	11.2%	4	3.4%	10	2.5%		
3	16	33.3%	72	42.6%	17	14.3%	71	18.0%	<.001	a.c.d.e.f
4	15	31.3%	51	30.2%	35	29.4%	139	35.3%		
5	14	29.2%	27	16.0%	63	52.9%	174	44.2%		
Cough and phlegm										
1	1	2.1%	1	0.6%	0	0.0%	0	0.0%		
2	3	6.3%	8	4.7%	2	1.7%	9	2.3%		
3	34	70.8%	99	58.6%	66	55.5%	155	39.3%	<.001	a.b.c.d.e.
4	9	18.8%	55	32.5%	49	41.2%	221	56.1%		
5	1	2.1%	6	3.6%	2	1.7%	9	2.3%		

Numerical values are shown (%)

p-values were calculated using the Kruskal-Wallis test post-hoc test: Mann–Whitney U test

a: Group A vs Group B

b:Group C vs Group D

c: Group A vs Group C

d: Group B vs Group D

e: Group A vs Group D

f:Group B vs Group C

a, b: Current smokers / ex-smokers (A and C Groups) vs never smokers (B and D Groups)

c, d: Diagnosis of obstructive ventilatory defect (A and B Groups) vs no obstructive ventilatory defect (C and D Groups)

For peer review only

Acknowledgements

We thank the study participants, technical staff, administrative support team, and our co-workers for their help. We thank the Environmental Restoration and Conservation Agency and Mizushima-Kyodo Hospital for their support.

Funding

This work was supported by the Environmental Restoration and Conservation Agency and Mizushima-Kyodo Hospital.

Competing interests

The authors declare that they have no competing interests.

Contributions

HS was the principal investigator and contributed to the design of the study, administered the funding, supervised the team's work and made critical revisions to the manuscript for intellectual content. KK designed the study, collected data, analysed and interpreted data, and prepared the manuscript. SH took part in analysing and interpreting the data, and made critical revisions to the manuscript for intellectual content. All other authors took part in data collection and interpretation, and have read and approved the final manuscript.

Data sharing statement:

Additional unpublished data from the study are available from TT (tanakataka@nagasaki-u.ac.jp) and YY (dependonhero@yahoo.co.jp). They can be reached by email.

For peer review only

REFERENCES

1. Yang G, Wang Y, Zeng Y, et al. Rapid health transition in China, 1990-2010: findings from the Global Burden of Disease Study 2010. Lancet 2013;**381**(9882):1987-2015 doi: 10.1016/S0140-6736(13)61097-1
S0140-6736(13)61097-1 [pii][published Online First: Epub Date]|.
2. Liu Y, Lee K, Perez-Padilla R, et al. Outdoor and indoor air pollution and COPD-related diseases in high- and low-income countries. Int J Tuberc Lung Dis 2008;**12**(2):115-27
3. Yang JY, Xin JY, Ji DS, et al. [Variation analysis of background atmospheric pollutants in North China during the summer of 2008 to 2011]. Huan Jing Ke Xue 2012;**33**(11):3693-704
4. Tanaka T, Asai M, Yanagita Y, et al. Longitudinal study of respiratory function and symptoms in a non-smoking group of long-term officially-acknowledged victims of pollution-related illness. BMC Public Health 2013;**13**:766 doi: 10.1186/1471-2458-13-766
1471-2458-13-766 [pii][published Online First: Epub Date]|.
5. Usmanova G, Mokdad AH. Results of the Global Youth Tobacco Survey and implementation of the WHO Framework Convention on Tobacco Control in the WHO Eastern Mediterranean Region (EMR) countries. J Epidemiol Glob Health 2013;**3**(4):217-34 doi: 10.1016/j.jegh.2013.07.001
S2210-6006(13)00084-1 [pii][published Online First: Epub Date]|.
6. Yang G, Fan L, Tan J, et al. Smoking in China: findings of the 1996 National Prevalence Survey. JAMA 1999;**282**(13):1247-53 doi: joc80784 [pii][published Online First: Epub Date]|.
7. M C. WHO report on the global tobacco epidemic 2008. Geneva:World Health Organization 2008
8. Yamagata T, Hirano T, Sugiura H, et al. Comparison of bronchodilatory properties of transdermal and inhaled long-acting beta 2-agonists. Pulm Pharmacol Ther 2008;**21**(1):160-5 doi: S1094-5539(07)00047-8 [pii]
10.1016/j.pupt.2007.05.004[published Online First: Epub Date]|.
9. Baldwin ED CA, Richards DW Jr. Pulmonary insufficiency; physiological classification, clinical methods of analysis, standard values in normal subjects. Medicine (Baltimore) 1948; **27(3)**:243-78
10. Curjuric I, Imboden M, Nadif R, et al. Different genes interact with particulate matter and tobacco smoke exposure in affecting lung function decline in the general population. PLoS One 2012;**7**(7):e40175 doi: 10.1371/journal.pone.0040175
PONE-D-11-21280 [pii][published Online First: Epub Date]|.
11. Ceylan E, Kocyigit A, Gencer M, et al. Jul 2006 patent 0954-6111 (Print)
0954-6111 (Linking).
12. Downs SH, Schindler C, Liu LJ, et al. Reduced exposure to PM10 and attenuated age-related decline in lung function. N Engl J Med 2007;**357**(23):2338-47 doi: 357/23/2338 [pii]
10.1056/NEJMoa073625[published Online First: Epub Date]|.
13. Lee PN, Fry JS. Systematic review of the evidence relating FEV1 decline to giving up smoking. BMC Med 2010;**8**:84 doi: 10.1186/1741-7015-8-84
1741-7015-8-84 [pii][published Online First: Epub Date]|.
14. Fletcher C, Peto R. The natural history of chronic airflow obstruction. British medical journal 1977;**1**(6077):1645-8
15. Downs SH, Schindler C, Liu LJ, et al. Reduced exposure to PM10 and attenuated age-related decline in lung function. N Engl J Med 2007;**357**(23):2338-47 doi: 10.1056/NEJMoa073625[published Online First: Epub Date]|.
16. Siroux V, Pin I, Oryszczyn MP, et al. Relationships of active smoking to asthma and asthma severity in the EGEA study. Epidemiological study on the Genetics and Environment of Asthma. Eur Respir J 2000;**15**(3):470-7

For peer review only

17. Gruffydd-Jones K. GOLD guidelines 2011: what are the implications for primary care? Primary care respiratory journal : journal of the General Practice Airways Group 2012;**21**(4):437-41 doi: 10.4104/pcrj.2012.00058[published Online First: Epub Date]|.
18. Pelkonen M, Notkola IL, Nissinen A, et al. Thirty-year cumulative incidence of chronic bronchitis and COPD in relation to 30-year pulmonary function and 40-year mortality: a follow-up in middle-aged rural men. Chest 2006;**130**(4):1129-37 doi: 130/4/1129 [pii]
10.1378/chest.130.4.1129[published Online First: Epub Date]|.
19. Berglund DJ, Abbey DE, Lebowitz MD, et al. Respiratory symptoms and pulmonary function in an elderly nonsmoking population. Chest 1999;**115**(1):49-59
20. Oga T TM, Hajiro T, Ikeda A, Nishimura K. Analysis of longitudinal changes in dyspnea of patients with chronic obstructive pulmonary disease: an observational study. Respir Res 2012;**13**:85
21. Dong GH, Zhang P, Sun B, et al. Long-term exposure to ambient air pollution and respiratory disease mortality in Shenyang, China: a 12-year population-based retrospective cohort study. Respiration; international review of thoracic diseases 2012;**84**(5):360-8 doi: 10.1159/000332930[published Online First: Epub Date]|.
22. C. Arden Pope I caRTB, Michelle C. Turner, Aaron Cohen, Daniel Krewski, Michael Jerrett, Susan M. Gapstur, and Michael J. Thun. Lung Cancer and Cardiovascular Disease Mortality Associated with Ambient Air Pollution and Cigarette Smoke: Shape of the Exposure–Response Relationships. Environ Health Perspect 2011
119(11): 1616–21
23. Kaufman G. Chronic obstructive pulmonary disease: diagnosis and management. Nurs Stand 2013;**27**(21):53-7, 60-2
24. website.http GIfCOLDG, //www.goldcopd.org/. Global strategy for the diagnosisGlobal strategy for the diagnosis. management and prevention of COPD, 2011.

For peer review only

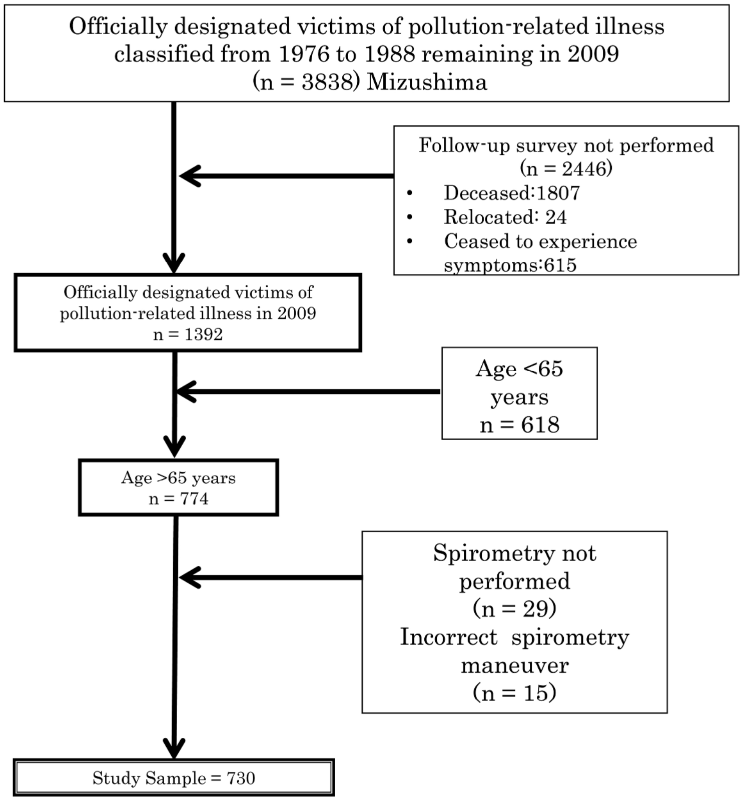

Figure 1: Flowchart showing the selection procedure for study subjects.
The study included officially designated victims of pollution-related illness in Kurashiki aged 65 years or older in 2009, and for whom full data were available for statistical analysis.
Ceased to experience symptoms: Officially designated victims who did renew the designation because of disappearance of clinical symptoms.

254x338mm (300 x 300 DPI)

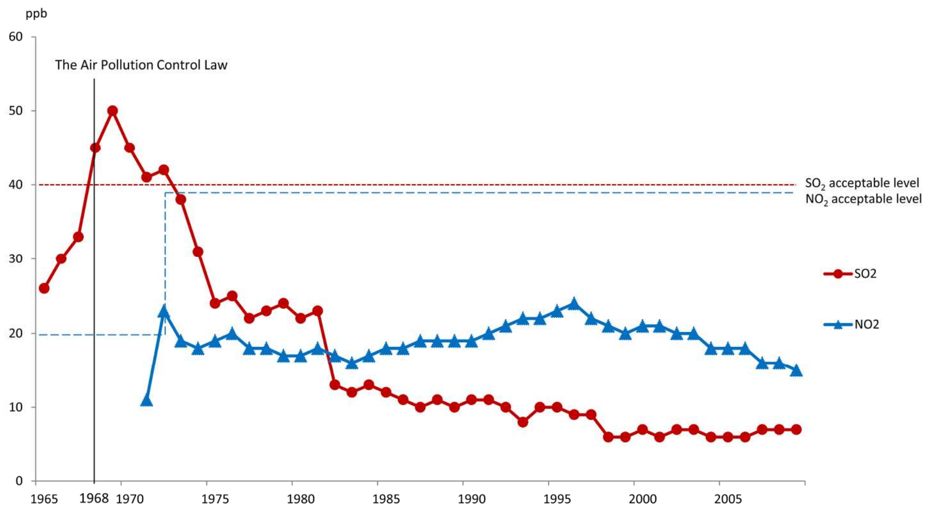

Figure 2 levels of air pollution

190x142mm (300 x 300 DPI)

STROBE 2007 (v4) Statement—Checklist of items that should be included in reports of *cohort studies*

Section/Topic	Item #	Recommendation	Reported on page #
**Title and abstract**	1	(*a*) Indicate the study's design with a commonly used term in the title or the abstract	
		(*b*) Provide in the abstract an informative and balanced summary of what was done and what was found	
**Introduction**			
Background/rationale	2	Explain the scientific background and rationale for the investigation being reported	
Objectives	3	State specific objectives, including any prespecified hypotheses	
**Methods**			
Study design	4	Present key elements of study design early in the paper	
Setting	5	Describe the setting, locations, and relevant dates, including periods of recruitment, exposure, follow-up, and data collection	
Participants	6	(*a*) Give the eligibility criteria, and the sources and methods of selection of participants. Describe methods of follow-up	
		(*b*) For matched studies, give matching criteria and number of exposed and unexposed	
Variables	7	Clearly define all outcomes, exposures, predictors, potential confounders, and effect modifiers. Give diagnostic criteria, if applicable	
Data sources/ measurement	8*	For each variable of interest, give sources of data and details of methods of assessment (measurement). Describe comparability of assessment methods if there is more than one group	
Bias	9	Describe any efforts to address potential sources of bias	
Study size	10	Explain how the study size was arrived at	
Quantitative variables	11	Explain how quantitative variables were handled in the analyses. If applicable, describe which groupings were chosen and why	
Statistical methods	12	(*a*) Describe all statistical methods, including those used to control for confounding	
		(*b*) Describe any methods used to examine subgroups and interactions	
		(*c*) Explain how missing data were addressed	
		(*d*) If applicable, explain how loss to follow-up was addressed	
		(*e*) Describe any sensitivity analyses	
**Results**			

For peer review only

Participants	13*	(a) Report numbers of individuals at each stage of study—eg numbers potentially eligible, examined for eligibility, confirmed eligible, included in the study, completing follow-up, and analysed	
		(b) Give reasons for non-participation at each stage	
		(c) Consider use of a flow diagram	
Descriptive data	14*	(a) Give characteristics of study participants (eg demographic, clinical, social) and information on exposures and potential confounders	
		(b) Indicate number of participants with missing data for each variable of interest	
		(c) Summarise follow-up time (eg, average and total amount)	
Outcome data	15*	Report numbers of outcome events or summary measures over time	
Main results	16	(*a*) Give unadjusted estimates and, if applicable, confounder-adjusted estimates and their precision (eg, 95% confidence interval). Make clear which confounders were adjusted for and why they were included	
		(*b*) Report category boundaries when continuous variables were categorized	
		(*c*) If relevant, consider translating estimates of relative risk into absolute risk for a meaningful time period	
Other analyses	17	Report other analyses done—eg analyses of subgroups and interactions, and sensitivity analyses	
**Discussion**			
Key results	18	Summarise key results with reference to study objectives	
**Limitations**			
Interpretation	20	Give a cautious overall interpretation of results considering objectives, limitations, multiplicity of analyses, results from similar studies, and other relevant evidence	
Generalisability	21	Discuss the generalisability (external validity) of the study results	
**Other information**			
Funding	22	Give the source of funding and the role of the funders for the present study and, if applicable, for the original study on which the present article is based	

*Give information separately for cases and controls in case-control studies and, if applicable, for exposed and unexposed groups in cohort and cross-sectional studies.

Note: An Explanation and Elaboration article discusses each checklist item and gives methodological background and published examples of transparent reporting. The STROBE checklist is best used in conjunction with this article (freely available on the Web sites of PLoS Medicine at http://www.plosmedicine.org/, Annals of Internal Medicine at http://www.annals.org/, and Epidemiology at http://www.epidem.com/). Information on the STROBE Initiative is available at www.strobe-statement.org.

For peer review only

Tobacco use among designated air pollution victims and its impact on lung function and respiratory symptoms: a retrospective cross-sectional study

Journal:	*BMJ Open*
Manuscript ID:	bmjopen-2014-005393.R2
Article Type:	Research
Date Submitted by the Author:	24-Jun-2014

Complete List of Authors:	kotaki, kenji; Nagasaki University Graduate School of Biomedical Sciences, Course of Cardiopulmonary Rehabilitation Science<br>Senjyu, Hideaki; Nagasaki University Graduate School of Biomedical Science, Course of Cardiopulmonary Rehabilitation Science<br>Tanaka, Takako; Nagasaki University Graduate School of Biomedical Science, Course of Cardiopulmonary Rehabilitation Science<br>Yano, Yudai; Nagasaki University Graduate School of Biomedical Science, Course of Cardiopulmonary Rehabilitation Science<br>Miyamoto, Naomi; Kio University Faculty of Health Science, Department of Physical Therapy<br>Nishinakagawa, Tsuyoshi; Nagasaki University Graduate School of Biomedical Science, Course of Cardiopulmonary Rehabilitation Science<br>Yanagita, Yorihide; Seirei Mikatahara General Hospital, Department of Rehabilitation Medicine<br>Asai, Masaharu; Nagasaki University Graduate School of Biomedical Science, Course of Cardiopulmonary Rehabilitation Science<br>Kozu, Ryo; Nagasaki University Hospital, Department of Rehabilitation Medicine<br>Tabusadani, Mitsuru; Nagasaki University, Center for Industry,University and Government Cooperation<br>Sawai, Terumitsu; Nagasaki University Graduate School of Biomedical Science, Course of Cardiopulmonary Rehabilitation Science<br>Honda, Sumihisa; Nagasaki University Graduate School of Biomedical Science, Course of Cardiopulmonary Rehabilitation Science

**Primary Subject Heading**:	Smoking and tobacco
Secondary Subject Heading:	Epidemiology, Respiratory medicine
Keywords:	EPIDEMIOLOGY, SOCIAL MEDICINE, Health & safety < HEALTH SERVICES ADMINISTRATION & MANAGEMENT, Public health < INFECTIOUS DISEASES, Chronic airways disease < THORACIC MEDICINE, PUBLIC HEALTH

SCHOLARONE™ Manuscripts

For peer review only

Title

Tobacco use among designated air pollution victims and its impact on lung function and respiratory symptoms: a retrospective cross-sectional study

Authors

Kenji Kotaki, Hideaki Senjyu*, Takako Tanaka, Yudai Yano, Naomi Miyamoto, Tsuyoshi Nishinakagawa, Yorihide Yanagita, Masaharu Asai, Ryo Kozu, Mitsuru Tabusadani, Terumitsu Sawai, Sumihisa Honda

Author details

Department of Cardiopulmonary Rehabilitation Science, Nagasaki University Graduate School of Biomedical Sciences, Nagasaki, Japan

***Corresponding author**

Department of Cardiopulmonary Rehabilitation Science

Nagasaki University Graduate School of Biomedical Sciences

1-7-1 Sakamoto, Nagasaki 852-8520, Japan

Email: senjyu@nagasaki-u.ac.jp

Tel: +81-95-819-7963; Fax: +81-95-819-7963

Keywords: Air pollution, Tobacco, Respiratory function, Respiratory symptoms, Pulmonary function

Word count: 3644 words

For peer review only - http://bmjopen.bmj.com/site/about/guidelines.xhtml

For peer review only

ABSTRACT

Objectives: We sought to elucidate the long-term effects of tobacco use in designated pollution victims with and without obstructive pulmonary defects.

Design: A retrospective cross-sectional study.

Setting: The register of pollution victims in Kurashiki, Japan.

Participants: 730 individuals over 65 years of age previously diagnosed with pollution-related respiratory disease. Patients were classified into four groups according to their smoking status and whether or not they had obstructive pulmonary disease. We then compared the prevalence of respiratory symptoms and lung function over time between groups.

Primary outcome measures: Spirometry was performed and a respiratory health questionnaire completed in the same season each year for up to 30 years.

Results: Rates of smoking and respiratory disease were high in our sample. While respiratory function in non-smoking patients did not completely recover, the annual rate of change in lung function was within the normal range ($p < 0.01$). However, smokers had worse lung function and were more likely to report more severe pulmonary symptoms ($p < 0.01$).

Conclusions: Patients' respiratory function did not fully recover despite improved air quality. Our results suggest that, in the context of exposure to air pollution, tobacco use causes additional loss of lung function and exacerbates respiratory symptoms.

Article summary

Strengths and limitations

- This first study to have analysed changes in respiratory function and respiratory symptoms in a group of designated pollution victims in Japan over up to 30 years.
- Tobacco use caused additional loss of lung function and exacerbated respiratory symptoms in those who have been exposed to air pollution.
- We did not have data on the quantity of cigarettes smoked, the duration of cigarette smoking, or individual-level exposure to environmental pollutants.

For peer review only

- We were unable to account for possible confounding factors such as socio-economic class and other lifestyle factors.

For peer review only

For peer review only - http://bmjopen.bmj.com/site/about/guidelines.xhtml

INTRODUCTION

In many Asian nations, particularly China, air pollution is increasingly being recognised as an important emerging environmental and public health issue. Previous work has shown that levels of ambient air pollution are associated with an elevated incidence of respiratory diseases and reported prevalence of respiratory symptoms,[1] particularly asthma, pulmonary emphysema and chronic bronchitis.[2] Atmospheric pollution caused by particulate matter less than 2.5 μm in diameter (PM2.5) is reported to be particularly acute in China and in surrounding countries as a result of wind dispersal. This is a growing public health issue in the region, especially given that PM 2.5 is capable of penetrating deep into lung tissue and precipitating a number of respiratory diseases.[3]

Japan was also severely affected during its period of accelerated economic growth in the wake of post-war reconstruction in the 1960s, with both air pollution and the prevalence of respiratory diseases rising markedly. In Kurashiki, a city located in Okayama Prefecture, reports of pulmonary toxicity owing to oxidant air pollutants rose by a factor of 1.73 among the city's inhabitants in the period leading up to 1970 as yearly average SO3 levels increased. The majority of excess pollution-related deaths in this period were caused by acute exacerbation of asthma.

Although patients do not commonly recover fully from pollution-related respiratory diseases, FEV1/ FVC ratios (calculated by dividing Forced Expiratory Volume exhaled at the end of the first second of forced expiration by Forced Vital Capacity) may return to within the normal range once air quality is improved.[4] As a result, Japan passed the Pollution-Related Health Damage Compensation Law in 1973 with the aim of identifying designated air pollution victims within affected areas for compensation. Patients with a diagnosis of bronchial asthma, chronic bronchitis or pulmonary emphysema who had been resident in any designated air pollution zone for a specified period of time were legally recognised as pollution victims. These designated victims were fully reimbursed the costs of all related medical treatment by the state. However, patients' lung function, as measured

For peer review only - http://bmjopen.bmj.com/site/about/guidelines.xhtml

using FEV1/FVC ratios, was not among the diagnostic criteria used to determine eligibility for compensation, as no agreed clinical definition for Chronic Obstructive Pulmonary Disease (COPD) existed at that time.

Tobacco smoke has been shown to contain high levels of PM 2.5, and is likely to have a number of effects in common with air pollution. Tobacco use has been shown to reduce lung function, as measured using FEV1/ FVC ratios, and has been identified as a cause of COPD and other tobacco-related diseases. The World Health Organization (WHO) Framework Convention on Tobacco Control was therefore adopted in 2003.[5] However, tobacco use remains a significant public health issue. According to a WHO report, more than 1.1 billion people globally are regular smokers.[6] Furthermore, Asia accounts for a third of total world cigarette consumption, and there has been a local rise in air pollution. [7]

Both air pollution and tobacco smoke have been shown to reduce patients' FEV1/FVC ratios, to increase the risk of obstructive ventilatory defects, and to exacerbate existing respiratory symptoms. Tanaka et al. have reported that when air quality is improved, the annual change in patients' FEV1 can return to within the normal range.[4] However, the effect of ongoing tobacco use on respiratory function and pulmonary symptoms in patients with already reduced FEV1/FVC ratios resulting from bronchial hypersensitivity due to air pollution has not yet been elucidated.

The purpose of the present study, carried out among a population of confirmed pollution victims living in a designated pollution-affected area, was to determine the effect of tobacco use on measures of respiratory function and prevalence of pulmonary symptoms up to 30 years after an improvement in air quality. In particular, we sought to compare these effects in patients with and without diagnosed obstructive ventilatory defects resulting from exposure to air pollution.

For peer review only - http://bmjopen.bmj.com/site/about/guidelines.xhtml

METHODS

Study design and setting

The present study was embedded in a retrospective cross-sectional analysis of lung function and respiratory symptoms in designated victims of pollution-related illness in Kurashiki between their initial evaluation (from 1976 to 1988) and follow-up in 2009. Study subjects were drawn from the register of pollution victims in Kurashiki. All study participants met the following conditions, as determined by the Public Relief System of Kurashiki City, in accordance with the Pollution-Related Health Damage Special Measures Law (1969) and the Pollution-Related Health Damage Compensation Law (1973): (1) they had resided or were employed within a designated air pollution zone, and (2) they were diagnosed with chronic bronchitis, asthma, or emphysema by a medical practitioner.

In accordance with the Public Nuisance Countermeasures Law (1967), registered victims were entitled to various forms of compensation including a monthly consultation with a doctor, yearly assessments of respiratory symptoms consisting of a detailed questionnaire and spirometry tests. These patients received treatment with inhaled corticosteroids and long-acting β2 agonists and expectorants, which may have caused improvements in lung function.[8] However, this study did not collect detailed data regarding treatment regimen, and we were therefore unable to evaluate the effects of treatment.

Of 419,203 total residents (204,958 males, 214,245 females), 3,838 (0.9%) were officially classified as designated pollution victims in the period up to 1988. The records of these 3,838 residents were reviewed in 2009 with the permission of the Kurashiki City Public Office (Figure 1). Of the 1,392 remaining ‘officially designated victims of pollution-related illness’, we screened 774 individuals aged ≥ 65 years for inclusion in the study, to focus the study on elderly people. The elderly people

For peer review only - http://bmjopen.bmj.com/site/about/guidelines.xhtml

have a higher risk factor for the onset of COPD and show remarkably more respiratory symptoms of COPD, as we restricted to the patients over 65 years in this study. After excluding 44 records that did not have complete spirometry data, the resulting sample size was 730.

We classified patients into two groups according to whether they had an obstructive impairment using the FEV1/FVC ratios recorded at their initial evaluation. Participants in each group were then further sub-divided according to whether they had ever smoked or not(current smoker or ex-smoker or ever smoked) – determined using self-reporting at follow-up, but we could not identify smoking period. This resulted in four comparison groups, labelled as Group A (n=48, current smokers and ex-smokers diagnosed with an obstructive impairment reporting), Group B (n=169, non-smokers diagnosed with an obstructive impairment), Group C (n=119, smokers and ex-smokers without a diagnosed obstructive impairment) and Group D (n=394, non-smokers without an obstructive impairment). The majority of these patients were diagnosed with chronic bronchitis (n = 528, 68.2%), asthma (n = 242, 31.3%), or emphysema (n = 4, 0.5%), as described in case reports provided by authorized medical practitioners.

The study protocol was approved by the Ethical Committee of the Nagasaki University Graduate School of Biomedical Sciences.

Clinical measurements

Patients' gender, height, weight, Body Mass Index (BMI), FVC, Vital Capacity (%VC), FEV1, FEV1/ FVC ratios, diagnosed respiratory conditions and respiratory symptoms were all recorded at baseline and at follow-up.

Spirometry measurements

Spirometry was performed by trained staff at the Mizushima-Kyodo Hospital using an electronic

For peer review only - http://bmjopen.bmj.com/site/about/guidelines.xhtml

spirometry device (FUDAC 70, Fukuda Sangyo Inc., Chiba, Japan). Tests were performed on patients in the sitting position, and were repeated until at least three reproducible forced expiratory curves had been obtained. Both FEV1 and FVC were measured and used to calculate FEV1/FVC ratios in addition to %VC using the method proposed by Baldwin et al. [9]

Annual change of spirometric data

The mean annual change in spirometry data was calculated by subtracting the follow-up results from the initial evaluation results and dividing by the number of years between initial evaluation and follow-up.

Respiratory symptoms

Respiratory symptoms (including dyspnoea, asthma attacks, cough, and sputum production) were assessed by physicians in the same season each year using a respiratory health questionnaire designed by the government of Japan. Each symptom was graded using a standardised five-point scale.

Dyspnoea

1. Too breathless to leave the house, or breathless when dressing or undressing.
2. Breathless after walking about 50 m or after a few minutes on level ground.
3. Breathless when walking on level ground and keeping up with people of the same age, but not breathless when walking at own pace.
4. Breathless when walking up a slight hill or the stairs.
5. Breathless only during strenuous exercise.

Wheeze

1. Severe episode ≥ 10 days each month during the last year.

For peer review only - http://bmjopen.bmj.com/site/about/guidelines.xhtml

2. Severe episode ≥ 5 days each month during the last year, or mild episode ≥ 10 days each month during the last year.

3. Severe episode ≥ 1 day each month during the last year, or mild episode ≥ 5 days each month during the last year.

4. Mild episode ≥ 1 day each month during the last year.

5. No episodes of wheezing.

Cough and sputum

1. Daily cough and sputum, with a large amount of sputum or difficulty clearing sputum.

2. Daily cough and sputum, with a moderate amount of sputum or difficulty clearing sputum.

3. Daily cough and sputum, but not troublesome during daily life.

4. Daily cough and sputum for ≤ 3 months each year.

5. No cough or sputum.

Air pollution monitoring

Data on mean daily concentrations of air pollutants were obtained at 21 points in Kurashiki. Monitoring of SO2 concentrations started in 1965, while monitoring of nitrogen dioxide (NO2) concentrations commenced in 1971.

Statistical analysis

Data from patients in each of the four comparison groups were compared using the Kruskal-Wallis test and the Mann-Whitney U test as a post-hoc test. Differences in gender distributions and the prevalence of respiratory diagnoses in each group were analysed using the Cochran-Armitage test, using the chi-square test to test for statistical significance. Changes in lung function between initial evaluation and follow-up were analysed using the Wilcoxon signed-rank test and the Mann-Whitney U test as a post-hoc test. A normality test was performed on all continuous variables using the

For peer review only - http://bmjopen.bmj.com/site/about/guidelines.xhtml

Kolmogorov-Smirnov test before analysis. All analyses were performed using PASW Statistics, version 18. Finally, p-values of <0.05 were considered statistically significant.

RESULTS

Air pollutants

Figure 2 shows the annual mean daily levels of SO_2 and NO_2 recorded from 1965 to 2009 in Kurashiki. The Air Pollution Control Law was enacted in 1968. SO_2 levels were above the acceptable level every year from 1968 to 1974 and then decreased to below 40 parts per billion (ppb), which is the acceptable level defined by the Air Pollution Control Law. In 1973, the acceptable NO_2 level was changed by the Air Pollution Control Law from 20 ppb to 40 ppb. The NO_2 level exceeded the revised acceptable level only in 1973.

Sample characteristics

Table 1 shows the characteristics of our patient sample at initial evaluation. We found significant differences in the age distributions between groups ($p<0.001$). Males outnumbered females in the Group A and Group C ($p<0.001$). Patients in the Group B and Group D had significantly lower heights and weights ($p<0.001$).

We found differences in FVC values between groups, with patients in the Group B and Group D presenting significantly lower values than those in the Group A and Group C ($p<0.001$). FEV_1 values, which also differed significantly between comparison groups ($p<0.001$), were lowest in the Group B, followed by in the Group A. %VC and FEV_1/FVC ratios were lowest the Group A and Group B. Finally, the prevalence of bronchial asthma at initial evaluation was much higher in the Group B compared with all other groups ($p<0.001$).

For peer review only - http://bmjopen.bmj.com/site/about/guidelines.xhtml

Change in respiratory function and symptoms between initial evaluation and follow-up

Table 2 and 3 show the results of our evaluation of the change in severity of respiratory symptoms and respiratory function between initial evaluation and follow-up. Respiratory function had deteriorated in almost all subjects by follow-up. In particular, FEV/FVC ratios in Group C, which averaged 79.1±5.6% at baseline, had fallen to 67.9±10.2% at follow-up because of the development of obstructive ventilatory defects in a number of subjects. Conversely, average FEV/FVC ratios increased from 59.8±10.3% to 62.1±13.4% in Group B ($p<0.001$). The median level of respiratory symptoms worsened from level II to level III in all groups. However, Group D saw a decrease in the proportion of patients reporting asthma attacks ($p=0.78$), cough, and sputum production ($p=0.24$) over the period studied.

Mean annual change in respiratory function

Table 4 shows the mean annual change in respiratory function in each comparison group.

Mean FEV1 decreased to a similar extent in both Group A and Group C. While there was a slight fall in FEV1 in Group D, there was a marginal improvement in FEV1 in Group B ($p<0.001$). At the same time, while FEV1/FVC ratios fell ($p<0.001$) in both Group A and Group C, an improvement was seen in Group B. Finally, %VC fell in all groups ($p<0.001$), although the decline was greater in Group A and Group B than in Group C and Group D, as shown in Table 4.

Comparison of respiratory symptoms at final evaluation

Table 5 shows the severity of respiratory symptoms in our four comparison groups at follow-up.

For peer review only - http://bmjopen.bmj.com/site/about/guidelines.xhtml

In Group A and Group B, whose subjects were diagnosed with obstructive ventilatory defects at baseline, the great majority reported severe dyspnoea, asthma, cough and sputum production. Severe dyspnoea was also highly prevalent in Group A and Group B. While severity of asthma was high in both Group B and Group D at initial evaluation, the proportion of patients reporting severe asthma had only increased significantly in Group A and Group C at follow-up ($p<0.001$). Severity of asthma was similar in Group C and Group D at follow-up. Prevalence of severe cough and sputum production increased significantly in Group A and Group C ($p<0.001$), with 80% of subjects in Group A reporting scores that changed from I to III due to a worsening of symptoms.

DISCUSSION

This retrospective cross-sectional study analysed changes in respiratory function and respiratory symptoms between two points in time (initial evaluation and follow-up) among a group of designated pollution victims in Japan. Our results show for the first time that improvements in respiratory function and reductions in respiratory symptoms are prevented by continuing tobacco use.

Among those patients diagnosed with an obstructive impairment due to air pollution exposure (Groups A and B), we found that FEV1/FVC ratios had deteriorated by follow-up with no reduction in morbidity. However, our results showed an improvement in FEV1/FVC ratios in Group B, which could be attributed to the high proportion of subjects in this group diagnosed with bronchial asthma at initial evaluation when compared with the other groups.

We observed that respiratory function had deteriorated among patients in Group A despite the improvement in local air quality, resulting in depressed FEV1/FVC ratios. Furthermore, while FEV1/FVC ratios in Group C were relatively low at the time of initial diagnosis, continuing tobacco use had resulted in some cases in obstructive impairment, causing FEV1/FVC ratios at follow-up to

For peer review only - http://bmjopen.bmj.com/site/about/guidelines.xhtml

decrease to 67.9±10.2%.

While air pollution can cause airway obstruction resulting in asthma, reactive airways dysfunction syndrome and reduced lung function, tobacco use, which can result in airway hyper-responsiveness, has been shown to exert a stronger influence on respiratory health in a number of studies.[10 11] These effects were evident in Group A and Group C, which showed similar annual rates of change in respiratory function over time. Conversely, Downs et al. have shown that, in non-smokers, annual rates of change in FEV1 can return to within the normal range in response to an improvement in air quality.[12] These findings are similar to the results of the present study. Previous studies have reported a mean annual rate of change in FEV1 of between -30 and -22 ml/year in healthy, non-smoking males and females aged > 65 years.[13] Patients with COPD had a mean annual change in FEV1 of 30–80 ml/year.[14] However, exposure to air pollution and tobacco smoke, both of which can result in tracheobronchial injury, can worsen annual rates of change in lung function markedly.

A high proportion of our subjects, particularly those with obstructive impairment, reported respiratory symptoms at baseline. These symptoms were more pronounced among the smokers in our sample. In Group D, however, the proportion of patients reporting asthma attacks, sputum production and cough had not increased, suggesting that these symptoms may be prevented by smoking cessation. The proportion of subjects in Group B reporting asthma attacks had increased by follow-up. Furthermore, the proportion of subjects reporting asthma attacks in Group C had increased to the same levels as that found in Group D at initial evaluation. Repeated bronchial inflammation caused by smoking and pollutant exposure can result in thickening of the bronchial wall and airway remodelling that cannot be reversed even by steroid treatment following the onset of hypersensitivity.[15] Furthermore, Siroux et al. have shown that continued tobacco use can significantly increase the incidence of asthma attacks.[16] Because of this association, smoking is listed as a potential trigger for asthma and COPD in asthma management guidelines.[17] Tobacco

For peer review only - http://bmjopen.bmj.com/site/about/guidelines.xhtml

use is also likely to have been the cause of the higher prevalence of severe cough and sputum production found in in Group A and Group C.

A number of studies have shown that smoking can cause respiratory tract inflammation and exacerbate symptoms such as cough and sputum production.[18]This was reflected in our finding that the severity of dyspnoea among subjects in Group A and Group B had increased markedly at follow-up. While a number of causes of dyspnoea have been identified, including age, poor physical fitness, muscle atrophy and social and environmental factors, we could not identify the primary determinants of dyspnoea in the present study.

[19] Finally, progression of respiratory disease and a decline in lung function can further aggravate these symptoms.[20]

In Asia, increased mortality and morbidity resulting from lung cancer, COPD, asthma and heart disorders caused by rising atmospheric PM 2.5 concentrations is a growing public health concern. According to the Organization for Economic Cooperation and Development, air pollution is expected to become the leading cause of death worldwide by 2050, and is likely to be responsible for between three and six million deaths annually.[21] Furthermore, Pope et al. have reported that worldwide pollution-related deaths could rise by 6% for each additional 10 μg/m3 increase in atmospheric PM 2.5 concentrations.[22]

The present study emphasizes the need to prevent or reduce air pollution and to improve public education on the negative health effects of smoking to reduce pulmonary morbidity on the population level. This is especially the case among designated victims, for whom additional education programs, behavioural interventions and support for smoking cessation would be particularly beneficial. One such behavioural intervention that has been proven to be cost-effective in reducing smoking prevalence is the five-step Global Initiative for Obstructive Lung Disease (GOLD) framework (ask, advise, assess, assist, arrange).[23] This strategy, which aims at delaying

For peer review only - http://bmjopen.bmj.com/site/about/guidelines.xhtml

the progress of COPD through continuing support for smoking cessation, may be applicable for use in similarly affected communities.[24]

This study has several limitations. First, information on number of cigarettes smoked and individual-level exposure to environmental pollutants was not available. Second, it was not within the scope of the present study to explore the role of gender-specific differences in exposure to tobacco smoke and pollutants – much of which may have been occupational. Third, we were unable to clearly distinguish between the effects of mainstream and side-stream cigarette smoke when considering the health effects of air pollution. Finally, we were unable to account for possible confounding factors such as socio-economic class and lifestyle factors, which may have resulted in individual-level differences in exposure to environmental pollutants.

Further work is needed to inform future prevention and treatment programs to improve the quality-of-life of the remaining designated pollution victims in Japan. The results of the present study are of particular importance in the context of high rates of smoking and worsening air quality in a number of Asian countries as they undergo rapid economic change.

For peer review only

For peer review only - http://bmjopen.bmj.com/site/about/guidelines.xhtml

Acknowledgements

We thank the study participants, technical staff, administrative support team, and our co-workers for their help. We thank the Environmental Restoration and Conservation Agency and Mizushima-Kyodo Hospital for their support.

Funding

This work was supported by the Environmental Restoration and Conservation Agency and Mizushima-Kyodo Hospital.

Competing interests

The authors declare that they have no competing interests.

Contributions

HS was the principal investigator and contributed to the design of the study, administered the funding, supervised the team's work and made critical revisions to the manuscript for intellectual content. KK designed the study, collected data, analysed and interpreted data, and prepared the manuscript. SH took part in analysing and interpreting the data, and made critical revisions to the manuscript for intellectual content. All other authors took part in data collection and interpretation, and have read and approved the final manuscript.

Data sharing statement:

Additional unpublished data from the study are available from TT (tanakataka@nagasaki-u.ac.jp) and YY (dependonhero@yahoo.co.jp). They can be reached by email.

For peer review only

Table 1. Demographics and respiratory characteristics of comparison groups at initial evaluation for designation of air pollution victim status

		Group A (n=48)	Group B (n=169)	Group C (n=119)	Group D (n=394)	p	post-hoc test
Age		54.3 ± 8.3	49.6 ± 8.2	49.8 ± 7.8	51.3 ± 8.9	<.001	b.c.f
Gender (male/female)		35/13	66/103	81/38	90/304	<.001	a.b.c.d.e.f
Height	(cm)	161.7 ± 6.3	155.2 ± 7.6	161.4 ± 6.1	152.8 ± 7.5	<.001	a.b.d.e.f
Weight	(kg)	56.9 ± 6.4	55.4 ± 9.3	59.1 ± 8.9	55.2 ± 9.7	<.001	b.f
BMI		21.8 ± 1.9	22.8 ± 3.6	22.6 ± 2.8	23.4 ± 3.8	<.001	a.b.d.e.
FVC	(l)	3.37 ± 0.79	3.04 ± 0.79	3.49 ± 0.76	2.94 ± 0.68	<.001	a.b.e.f
%VC	(%)	105.5 ± 18.7	103.5 ± 18.5	108.3 ± 15.5	107.6 ± 17.3	<.05	d.f
FEV1	(l)	2.09 ± 0.64	1.72 ± 0.58	2.67 ± 0.58	2.24 ± 0.58	<.001	a.b.c.d.f
FEV1/FVC	(%)	64.9 ± 6.5	59.8 ± 10.3	79.1 ± 5.6	79.6 ± 7.1	<.001	a.c.d.e.f
Data of duration after certification	(yr)	25.9±4.1	26.9±4.4	24.8±4.2	25.5±4.6	<.001	d.f
Diagnosed pulmonary disease CB,BA,PE CB+BA CB+PE, BA+PE	no	32/10/1/5/ 0/ 0	52/98/3/11/3/2	98/14/0/7/ 0/ 0	249/99/0/46/0/0	<.001	a.c.d.f
	Unit Score						
	1	0 (0.0%)	1(0.6%)	0 (0.0%)	0 (0.0%)		
	2	0 (0.0%)	4 (2.4%)	0 (0.0%)	4 (1.1%)		
Dyspnea	3	9 (18.8%)	31 (18.3%)	7 (5.9%)	55 (15.8%)	<.001	b.c.d.f
	4	30 (62.5%)	111 (65.7%)	75 (63.0%)	246 (70.5%)		
	5	9 (18.8%)	22 (13.0%)	37 (31.1%)	89 (25.5%)		
	1	0 (0.0%)	1 (0.6%)	0 (0.0%)	1 (0.3%)		
	2	0 (0.0%)	8 (4.7%)	1 (0.8%)	13 (3.3%)		
Asthma attack	3	7 (14.6%)	51 (30.2%)	11 (9.2%)	66 (16.8%)	<.001	a.b.d.f
	4	14 (29.2%)	76 (45.0%)	30 (25.2%)	139 (35.3%)		
	5	27 (56.3%)	33 (19.5%)	77 (64.7%)	175 (44.4%)		
	1	0 (0.0%)	2 (1.2%)	0 (0.0%)	0 (0.0%)		
	2	0 (0.0%)	5 (3.0%)	1 (0.8%)	9 (2.3%)		
Cough and phlegm	3	24 (50.0%)	50 (29.6%)	58 (48.7%)	143 (36.3%)	<.05	a.b.d.f
	4	23 (47.9%)	90 (53.3%)	58 (48.7%)	225 (57.1%)		
	5	1 (2.1%)	22 (13.0%)	1 (0.8%)	15 (3.8%)		

For peer review only

For peer review only - http://bmjopen.bmj.com/site/about/guidelines.xhtml

Figures are presented as mean ±SD

FEV1: Forced Expiratory Volume in 1 second, FVC: Forced Vital Capacity, VC%: % Vital Capacity

FEV1/FVC (%) Forced Expiratory Volume in 1 second.

CB: Chronic Bronchitis BA: Bronchial Asthma PE: Pulmonary Emphysema

CB+BA CB+PE BA+PE

Differences in sample characteristics analysed using the Kruskal-Wallis test with Cochran-Armitage tests for significance

post-hoc tests: Mann-Whitney U test, chi-square test for significance

a: Group A vs Group B

b:Group C vs Group D

c: Group A vs Group C

d: Group B vs Group D

e: Group A vs Group D

f:Group B vs Group C

a, b: Current smokers / ex-smokers (A and C Groups) vs never smokers (B and D Groups)

c, d: Diagnosis of obstructive ventilatory defect (A and B Groups) vs no obstructive ventilatory defect (C and D Groups)

For peer review only

Table 2. Change in demographic characteristics and respiratory function of designated air pollution victims by comparison group between initial evaluation and follow-up

		Initial evaluation	Follow-up	p	Initial evaluation	Follow-up	p	Initial evaluation	Follow-up	p	Initial evaluation	Follow-up	p
		**Group A**			**Group B**			**Group C**			**Group D**		
Age		54.3 ± 8.3	78.6 ± 6.5	<.001	49.6 ± 8.2	77.3 ± 7.5	<.001	49.8 ± 7.8	74.2 ± 7.1	<.001	51.3 ± 8.9	76.5 ± 7.8	<.001
Height	(cm)	161.7 ± 6.3	159.3 ± 6.9	<.001	155.2 ± 7.6	150.8 ± 8.8	<.001	161.4 ± 6.1	159.2 ± 6.2	<.001	152.8 ± 7.5	148.9 ± 8.8	<.001
Weight	(kg)	56.9 ± 6.4	55.4 ± 9.2	<.001	55.4 ± 9.3	52.1 ± 10.9	<.001	59.1 ± 8.9	58.9 ± 9.6	<.001	55.2 ± 9.7	53.2 ± 11.3	<.001
BMI		21.8 ± 1.9	21.7 ± 2.9	<.001	22.8 ± 3.6	22.7 ± 3.9	<.001	22.6 ± 2.8	23.2 ± 3.1	<.05	23.4 ± 3.8	23.9 ± 4.2	0.13
PFT													
FVC	(l)	3.37 ± 0.79	2.54 ± 0.85	<.001	3.04 ± 0.79	2.21 ± 0.73	<.001	3.49 ± 0.76	2.78 ± 0.81	<.001	2.94 ± 0.68	2.28 ± 0.69	<.001
%VC	(%)	105.5 ± 18.7	93.1 ± 21.7	<.001	103.5 ± 18.5	90.3 ± 22.3	<.001	108.3 ± 15.5	100.2 ± 21.9	<.001	107 ± 17.3	100.4 ± 23.4	<.001
FEV1	(l)	2.09 ± 0.64	1.28 ± 0.53	<.001	1.72 ± 0.58	1.29 ± 0.51	<.001	2.67 ± 0.58	1.8 ± 0.68	<.001	2.24 ± 0.58	1.61 ± 0.52	<.001
FEV1 /FVC	(%)	64.9 ± 6.5	54.5 ± 2.3	<.001	59.8 ± 10.3	62.1 ± 13.4	<.001	79.1 ± 5.6	67.9 ± 10.2	<.001	79.6 ± 7.1	73.8 ± 13.8	<.001

PFT: Pulmonary Function Testing

Figures are presented as mean ±SD

Initial evaluation versus follow-up: Mann-Whitney U test

For peer review only

Table 3. Change in respiratory symptoms of designated air pollution victims by comparison group between initial evaluation and follow-up

Unit Score	Initial evaluation		Follow-up		p	Initial evaluation		Follow-up		p	Initial evaluation		Follow-up		p	Initial evaluation		Follow-up		p
Dyspnoea	Group A					Group B					Group C					Group D				
1	0	0.0%	1	2.1%		1	0.6%	2	1.2%		0	0.0%	1	0.8%		0	0.0%	0	0.0%	
2	0	0.0%	4	8.3%		4	2.4%	15	8.9%		0	0.0%	2	1.7%		4	1.1%	13	3.7%	
3	9	18.8%	24	50.0%	<.001	31	18.3%	74	43.8%	<.001	7	5.9%	20	16.8%	<.001	55	15.8%	67	19.2%	<.001
4	30	62.5%	15	31.3%		111	65.7%	75	44.4%		75	63.0%	77	64.7%		246	70.5%	279	79.9%	
5	9	18.8%	4	8.3%		22	13.0%	3	1.8%		37	31.1%	19	16.0%		89	25.5%	35	10.0%	
Asthma attack																				
1	0	0.0%	0	0.0%		1	0.6%	0	0.0%		0	0.0%	0	0.0%		1	0.3%	0	0.0%	
2	0	0.0%	3	6.3%		8	4.7%	19	11.2%		1	0.8%	4	3.4%		13	3.3%	10	2.5%	
3	7	14.6%	16	33.3%	<.001	51	30.2%	72	42.6%	<.001	11	9.2%	17	14.3%	<.001	66	16.8%	71	18.0%	0.78
4	14	29.2%	15	31.3%		76	45.0%	51	30.2%		30	25.2%	35	29.4%		139	35.3%	139	35.3%	
5	27	56.3%	14	29.2%		33	19.5%	27	16.0%		77	64.7%	63	52.9%		175	44.4%	174	44.2%	
Cough and phlegm																				
1	0	0.0%	1	2.1%		2	1.2%	1	0.6%		0	0.0%	0	0.0%		0	0.0%	0	0.0%	
2	0	0.0%	3	6.3%		5	3.0%	8	4.7%		1	0.8%	2	1.7%		9	2.3%	9	2.3%	
3	24	50.0%	34	70.8%	<.001	50	29.6%	99	58.6%	<.001	58	48.7%	66	55.5%	<.001	143	36.3%	155	39.3%	0.24
4	23	47.9%	9	18.8%		90	53.3%	55	32.5%		58	48.7%	49	41.2%		227	57.1%	221	56.1%	
5	1	2.1%	1	2.1%		22	13.0%	6	3.6%		2	0.8%	2	1.7%		15	3.8%	9	2.3%	

Respiratory symptoms

Numerical values are shown (%).

Initial evaluation versus follow-up: Wilcoxon signed-rank test

For peer review only

Table 4. Mean annual change in yearly spirometry results from initial evaluation to follow-up by comparison group

		Group A (n=48)	Group B (n=169)	Group C (n=119)	Group D (n=394)	p	post-hoc test
FEV1 decline/year	(ml)	-36.7±19.1	-26.5±18.5	-37.2±16.4	-25.5±17.2	<.001	a.b.d.e.f
FEV1/FVC decline/year	(%)	-0.04±0.05	0.09±0.06	-0.04±0.03	-0.02±0.04	<.001	a.b.d.e.f
FVC decline/year	(ml)	-31.7±24.5	-30.1±20.7	-29.9±22.8	-25.3±17.1	<.05	d
%VC decline/year	(%)	-0.05±0.08	-0.05±0.08	-0.03±0.06	-0.03±0.07	<.001	c.d.e.f

Figures are presented as mean ±SD

p-values were calculated using the Kruskal-Wallis test post-hoc test: Mann-Whitney U test

a: Group A vs Group B

b:Group C vs Group D

c: Group A vs Group C

d: Group B vs Group D

e: Group A vs Group D

f:Group B vs Group C

a, b: Current smokers / ex-smokers (A and C Groups) vs never smokers (B and D Groups)

c, d: Diagnosis of obstructive ventilatory defect (A and B Groups) vs no obstructive ventilatory defect (C and D Groups)

For peer review only

For peer review only - http://bmjopen.bmj.com/site/about/guidelines.xhtml

Table 5. Change in severity of smoking-related respiratory symptoms by comparison group at follow-up

Score	Group A (n=48)		Group B (n=169)		Group C (n=119)		Group D (n=394)		p	post-hoc test
Dyspnea										
1	1	2.1%	2	1.2%	1	0.8%	0	0.0%		
2	4	8.3%	15	8.9%	2	1.7%	13	3.7%		
3	24	50.0%	74	43.8%	20	16.8%	67	19.2%	<.001	c.d.e.f
4	15	31.3%	75	44.4%	77	64.7%	279	79.9%		
5	4	8.3%	3	1.8%	19	16.0%	35	10.0%		
Asthma attack										
1	0	0.0%	0	0.0%	0	0.0%	0	0.0%		
2	3	6.3%	19	11.2%	4	3.4%	10	2.5%		
3	16	33.3%	72	42.6%	17	14.3%	71	18.0%	<.001	a.c.d.e.f
4	15	31.3%	51	30.2%	35	29.4%	139	35.3%		
5	14	29.2%	27	16.0%	63	52.9%	174	44.2%		
Cough and phlegm										
1	1	2.1%	1	0.6%	0	0.0%	0	0.0%		
2	3	6.3%	8	4.7%	2	1.7%	9	2.3%		
3	34	70.8%	99	58.6%	66	55.5%	155	39.3%	<.001	a.b.c.d.e.
4	9	18.8%	55	32.5%	49	41.2%	221	56.1%		
5	1	2.1%	6	3.6%	2	1.7%	9	2.3%		

Numerical values are shown (%)

p-values were calculated using the Kruskal-Wallis test post-hoc test: Mann–Whitney U test

a: Group A vs Group B

b:Group C vs Group D

c: Group A vs Group C

d: Group B vs Group D

e: Group A vs Group D

f:Group B vs Group C

a, b: Current smokers / ex-smokers (A and C Groups) vs never smokers (B and D Groups)

c, d: Diagnosis of obstructive ventilatory defect (A and B Groups) vs no obstructive ventilatory defect (C and D Groups)

For peer review only

REFERENCES

1. Yang G, Wang Y, Zeng Y, et al. Rapid health transition in China, 1990-2010: findings from the Global Burden of Disease Study 2010. Lancet 2013;**381**(9882):1987-2015 doi: 10.1016/S0140-6736(13)61097-1
S0140-6736(13)61097-1 [pii][published Online First: Epub Date]|.
2. Liu Y, Lee K, Perez-Padilla R, et al. Outdoor and indoor air pollution and COPD-related diseases in high- and low-income countries. Int J Tuberc Lung Dis 2008;**12**(2):115-27
3. Yang JY, Xin JY, Ji DS, et al. [Variation analysis of background atmospheric pollutants in North China during the summer of 2008 to 2011]. Huan Jing Ke Xue 2012;**33**(11):3693-704
4. Tanaka T, Asai M, Yanagita Y, et al. Longitudinal study of respiratory function and symptoms in a non-smoking group of long-term officially-acknowledged victims of pollution-related illness. BMC Public Health 2013;**13**:766 doi: 10.1186/1471-2458-13-766
1471-2458-13-766 [pii][published Online First: Epub Date]|.
5. Usmanova G, Mokdad AH. Results of the Global Youth Tobacco Survey and implementation of the WHO Framework Convention on Tobacco Control in the WHO Eastern Mediterranean Region (EMR) countries. J Epidemiol Glob Health 2013;**3**(4):217-34 doi: 10.1016/j.jegh.2013.07.001
S2210-6006(13)00084-1 [pii][published Online First: Epub Date]|.
6. Yang G, Fan L, Tan J, et al. Smoking in China: findings of the 1996 National Prevalence Survey. JAMA 1999;**282**(13):1247-53 doi: joc80784 [pii][published Online First: Epub Date]|.
7. M C. WHO report on the global tobacco epidemic 2008. Geneva:World Health Organization 2008
8. Yamagata T, Hirano T, Sugiura H, et al. Comparison of bronchodilatory properties of transdermal and inhaled long-acting beta 2-agonists. Pulm Pharmacol Ther 2008;**21**(1):160-5 doi: S1094-5539(07)00047-8 [pii]
10.1016/j.pupt.2007.05.004[published Online First: Epub Date]|.
9. Baldwin ED CA, Richards DW Jr. Pulmonary insufficiency; physiological classification, clinical methods of analysis, standard values in normal subjects. Medicine (Baltimore) 1948; **27(3)**:243-78
10. Curjuric I, Imboden M, Nadif R, et al. Different genes interact with particulate matter and tobacco smoke exposure in affecting lung function decline in the general population. PLoS One 2012;**7**(7):e40175 doi: 10.1371/journal.pone.0040175
PONE-D-11-21280 [pii][published Online First: Epub Date]|.
11. Ceylan E, Kocyigit A, Gencer M, et al. Jul 2006 patent 0954-6111 (Print)
0954-6111 (Linking).
12. Downs SH, Schindler C, Liu LJ, et al. Reduced exposure to PM10 and attenuated age-related decline in lung function. N Engl J Med 2007;**357**(23):2338-47 doi: 357/23/2338 [pii]
10.1056/NEJMoa073625[published Online First: Epub Date]|.
13. Lee PN, Fry JS. Systematic review of the evidence relating FEV1 decline to giving up smoking. BMC Med 2010;**8**:84 doi: 10.1186/1741-7015-8-84
1741-7015-8-84 [pii][published Online First: Epub Date]|.
14. Fletcher C, Peto R. The natural history of chronic airflow obstruction. British medical journal 1977;**1**(6077):1645-8
15. Downs SH, Schindler C, Liu LJ, et al. Reduced exposure to PM10 and attenuated age-related decline in lung function. N Engl J Med 2007;**357**(23):2338-47 doi: 10.1056/NEJMoa073625[published Online First: Epub Date]|.
16. Siroux V, Pin I, Oryszczyn MP, et al. Relationships of active smoking to asthma and asthma severity in the EGEA study. Epidemiological study on the Genetics and Environment of Asthma. Eur Respir J 2000;**15**(3):470-7

For peer review only - http://bmjopen.bmj.com/site/about/guidelines.xhtml

17. Gruffydd-Jones K. GOLD guidelines 2011: what are the implications for primary care? Primary care respiratory journal : journal of the General Practice Airways Group 2012;**21**(4):437-41 doi: 10.4104/pcrj.2012.00058[published Online First: Epub Date]|.
18. Pelkonen M, Notkola IL, Nissinen A, et al. Thirty-year cumulative incidence of chronic bronchitis and COPD in relation to 30-year pulmonary function and 40-year mortality: a follow-up in middle-aged rural men. Chest 2006;**130**(4):1129-37 doi: 130/4/1129 [pii]
10.1378/chest.130.4.1129[published Online First: Epub Date]|.
19. Berglund DJ, Abbey DE, Lebowitz MD, et al. Respiratory symptoms and pulmonary function in an elderly nonsmoking population. Chest 1999;**115**(1):49-59
20. Oga T TM, Hajiro T, Ikeda A, Nishimura K. Analysis of longitudinal changes in dyspnea of patients with chronic obstructive pulmonary disease: an observational study. Respir Res 2012;**13**:85
21. Dong GH, Zhang P, Sun B, et al. Long-term exposure to ambient air pollution and respiratory disease mortality in Shenyang, China: a 12-year population-based retrospective cohort study. Respiration; international review of thoracic diseases 2012;**84**(5):360-8 doi: 10.1159/000332930[published Online First: Epub Date]|.
22. C. Arden Pope I caRTB, Michelle C. Turner, Aaron Cohen et al. Lung Cancer and Cardiovascular Disease Mortality Associated with Ambient Air Pollution and Cigarette Smoke: Shape of the Exposure–Response Relationships. Environ Health Perspect 2011
119(11): 1616–21
23. Kaufman G. Chronic obstructive pulmonary disease: diagnosis and management. Nurs Stand 2013;**27**(21):53-7, 60-2
24. website.http GIfCOLDG, //www.goldcopd.org/. Global strategy for the diagnosisGlobal strategy for the diagnosis. management and prevention of COPD, 2011.

Figure legends:

Figure 1: Flowchart showing the selection procedure for study subjects.
Figure 2. Levels of air pollution

For peer review only - http://bmjopen.bmj.com/site/about/guidelines.xhtml

Title

Tobacco use among designated air pollution victims and its impact on lung function and respiratory symptoms: a retrospective cross-sectional study

Authors

Kenji Kotaki, Hideaki Senjyu*, Takako Tanaka, Yudai Yano, Naomi Miyamoto, Tsuyoshi Nishinakagawa, Yorihide Yanagita, Masaharu Asai, Ryo Kozu, Mitsuru Tabusadani, Terumitsu Sawai, Sumihisa Honda

Author details

Department of Cardiopulmonary Rehabilitation Science, Nagasaki University Graduate School of Biomedical Sciences, Nagasaki, Japan

***Corresponding author**

Department of Cardiopulmonary Rehabilitation Science

Nagasaki University Graduate School of Biomedical Sciences

1-7-1 Sakamoto, Nagasaki 852-8520, Japan

Email: senjyu@nagasaki-u.ac.jp

Tel: +81-95-819-7963; Fax: +81-95-819-7963

Keywords: Air pollution, Tobacco, Respiratory function, Respiratory symptoms, Pulmonary function

Word count: 3644 words

For peer review only

For peer review only - http://bmjopen.bmj.com/site/about/guidelines.xhtml

ABSTRACT

Objectives: We sought to elucidate the long-term effects of tobacco use in designated pollution victims with and without obstructive pulmonary defects.

Design: A retrospective cross-sectional study.

Setting: The register of pollution victims in Kurashiki, Japan.

Participants: 730 individuals over 65 years of age previously diagnosed with pollution-related respiratory disease. Patients were classified into four groups according to their smoking status and whether or not they had obstructive pulmonary disease. We then compared the prevalence of respiratory symptoms and lung function over time between groups.

Primary outcome measures: Spirometry was performed and a respiratory health questionnaire completed in the same season each year for up to 30 years.

Results: Rates of smoking and respiratory disease were high in our sample. While respiratory function in non-smoking patients did not completely recover, the annual rate of change in lung function was within the normal range ($p < 0.01$). However, smokers had worse lung function and were more likely to report more severe pulmonary symptoms ($p < 0.01$).

Conclusions: Patients' respiratory function did not fully recover despite improved air quality. Our results suggest that, in the context of exposure to air pollution, tobacco use causes additional loss of lung function and exacerbates respiratory symptoms.

Article summary

Strengths and limitations

- This first study to have analysed changes in respiratory function and respiratory symptoms in a group of designated pollution victims in Japan over up to 30 years.
- Tobacco use caused additional loss of lung function and exacerbated respiratory symptoms in those who have been exposed to air pollution.
- We did not have data on the quantity of cigarettes smoked, the duration of cigarette smoking, or individual-level exposure to environmental pollutants.

For peer review only - http://bmjopen.bmj.com/site/about/guidelines.xhtml

- We were unable to account for possible confounding factors such as socio-economic class and other lifestyle factors.

For peer review only

For peer review only - http://bmjopen.bmj.com/site/about/guidelines.xhtml

INTRODUCTION

In many Asian nations, particularly China, air pollution is increasingly being recognised as an important emerging environmental and public health issue. Previous work has shown that levels of ambient air pollution are associated with an elevated incidence of respiratory diseases and reported prevalence of respiratory symptoms,[1] particularly asthma, pulmonary emphysema and chronic bronchitis.[2] Atmospheric pollution caused by particulate matter less than 2.5 μm in diameter (PM2.5) is reported to be particularly acute in China and in surrounding countries as a result of wind dispersal. This is a growing public health issue in the region, especially given that PM 2.5 is capable of penetrating deep into lung tissue and precipitating a number of respiratory diseases.[3]

Japan was also severely affected during its period of accelerated economic growth in the wake of post-war reconstruction in the 1960s, with both air pollution and the prevalence of respiratory diseases rising markedly. In Kurashiki, a city located in Okayama Prefecture, reports of pulmonary toxicity owing to oxidant air pollutants rose by a factor of 1.73 among the city's inhabitants in the period leading up to 1970 as yearly average SO3 levels increased. The majority of excess pollution-related deaths in this period were caused by acute exacerbation of asthma.

Although patients do not commonly recover fully from pollution-related respiratory diseases, FEV1/ FVC ratios (calculated by dividing Forced Expiratory Volume exhaled at the end of the first second of forced expiration by Forced Vital Capacity) may return to within the normal range once air quality is improved.[4] As a result, Japan passed the Pollution-Related Health Damage Compensation Law in 1973 with the aim of identifying designated air pollution victims within affected areas for compensation. Patients with a diagnosis of bronchial asthma, chronic bronchitis or pulmonary emphysema who had been resident in any designated air pollution zone for a specified period of time were legally recognised as pollution victims. These designated victims were fully reimbursed the costs of all related medical treatment by the state. However, patients' lung function, as measured

For peer review only - http://bmjopen.bmj.com/site/about/guidelines.xhtml

using FEV1/FVC ratios, was not among the diagnostic criteria used to determine eligibility for compensation, as no agreed clinical definition for Chronic Obstructive Pulmonary Disease (COPD) existed at that time.

Tobacco smoke has been shown to contain high levels of PM 2.5, and is likely to have a number of effects in common with air pollution. Tobacco use has been shown to reduce lung function, as measured using FEV1/ FVC ratios, and has been identified as a cause of COPD and other tobacco-related diseases. The World Health Organization (WHO) Framework Convention on Tobacco Control was therefore adopted in 2003.[5] However, tobacco use remains a significant public health issue. According to a WHO report, more than 1.1 billion people globally are regular smokers.[6] Furthermore, Asia accounts for a third of total world cigarette consumption, and there has been a local rise in air pollution. [7]

Both air pollution and tobacco smoke have been shown to reduce patients' FEV1/FVC ratios, to increase the risk of obstructive ventilatory defects, and to exacerbate existing respiratory symptoms. Tanaka et al. have reported that when air quality is improved, the annual change in patients' FEV1 can return to within the normal range.[4] However, the effect of ongoing tobacco use on respiratory function and pulmonary symptoms in patients with already reduced FEV1/FVC ratios resulting from bronchial hypersensitivity due to air pollution has not yet been elucidated.

The purpose of the present study, carried out among a population of confirmed pollution victims living in a designated pollution-affected area, was to determine the effect of tobacco use on measures of respiratory function and prevalence of pulmonary symptoms up to 30 years after an improvement in air quality. In particular, we sought to compare these effects in patients with and without diagnosed obstructive ventilatory defects resulting from exposure to air pollution.

For peer review only - http://bmjopen.bmj.com/site/about/guidelines.xhtml

METHODS

Study design and setting

The present study was embedded in a retrospective cross-sectional analysis of lung function and respiratory symptoms in designated victims of pollution-related illness in Kurashiki between their initial evaluation (from 1976 to 1988) and follow-up in 2009. Study subjects were drawn from the register of pollution victims in Kurashiki. All study participants met the following conditions, as determined by the Public Relief System of Kurashiki City, in accordance with the Pollution-Related Health Damage Special Measures Law (1969) and the Pollution-Related Health Damage Compensation Law (1973): (1) they had resided or were employed within a designated air pollution zone, and (2) they were diagnosed with chronic bronchitis, asthma, or emphysema by a medical practitioner.

In accordance with the Public Nuisance Countermeasures Law (1967), registered victims were entitled to various forms of compensation including a monthly consultation with a doctor, yearly assessments of respiratory symptoms consisting of a detailed questionnaire and spirometry tests. These patients received treatment with inhaled corticosteroids and long-acting β2 agonists and expectorants, which may have caused improvements in lung function.[8] However, this study did not collect detailed data regarding treatment regimen, and we were therefore unable to evaluate the effects of treatment.

Of 419,203 total residents (204,958 males, 214,245 females), 3,838 (0.9%) were officially classified as designated pollution victims in the period up to 1988. The records of these 3,838 residents were reviewed in 2009 with the permission of the Kurashiki City Public Office (Figure 1). Of the 1,392 remaining ‘officially designated victims of pollution-related illness’, we screened 774 individuals aged ≥ 65 years for inclusion in the study, to focus the study on elderly people. The elderly people

For peer review only - http://bmjopen.bmj.com/site/about/guidelines.xhtml

have a higher risk factor for the onset of COPD and show remarkably more respiratory symptoms of COPD, as we restricted to the patients over 65 years in this study. After excluding 44 records that did not have complete spirometry data, the resulting sample size was 730.

We classified patients into two groups according to whether they had an obstructive impairment using the FEV1/FVC ratios recorded at their initial evaluation. Participants in each group were then further sub-divided according to whether they had ever smoked or not(current smoker or ex-smoker or ever smoked) – determined using self-reporting at follow-up, but we could not identify smoking period. This resulted in four comparison groups, labelled as Group A (n=48, current smokers and ex-smokers diagnosed with an obstructive impairment reporting), Group B (n=169, non-smokers diagnosed with an obstructive impairment), Group C (n=119, smokers and ex-smokers without a diagnosed obstructive impairment) and Group D (n=394, non-smokers without an obstructive impairment). The majority of these patients were diagnosed with chronic bronchitis (n = 528, 68.2%), asthma (n = 242, 31.3%), or emphysema (n = 4, 0.5%), as described in case reports provided by authorized medical practitioners.

The study protocol was approved by the Ethical Committee of the Nagasaki University Graduate School of Biomedical Sciences.

Clinical measurements

Patients' gender, height, weight, Body Mass Index (BMI), FVC, Vital Capacity (%VC), FEV1, FEV1/ FVC ratios, diagnosed respiratory conditions and respiratory symptoms were all recorded at baseline and at follow-up.

Spirometry measurements

Spirometry was performed by trained staff at the Mizushima-Kyodo Hospital using an electronic

For peer review only - http://bmjopen.bmj.com/site/about/guidelines.xhtml

spirometry device (FUDAC 70, Fukuda Sangyo Inc., Chiba, Japan). Tests were performed on patients in the sitting position, and were repeated until at least three reproducible forced expiratory curves had been obtained. Both FEV1 and FVC were measured and used to calculate FEV1/FVC ratios in addition to %VC using the method proposed by Baldwin et al. [9]

Annual change of spirometric data

The mean annual change in spirometry data was calculated by subtracting the follow-up results from the initial evaluation results and dividing by the number of years between initial evaluation and follow-up.

Respiratory symptoms

Respiratory symptoms (including dyspnoea, asthma attacks, cough, and sputum production) were assessed by physicians in the same season each year using a respiratory health questionnaire designed by the government of Japan. Each symptom was graded using a standardised five-point scale.

Dyspnoea

1. Too breathless to leave the house, or breathless when dressing or undressing.
2. Breathless after walking about 50 m or after a few minutes on level ground.
3. Breathless when walking on level ground and keeping up with people of the same age, but not breathless when walking at own pace.
4. Breathless when walking up a slight hill or the stairs.
5. Breathless only during strenuous exercise.

Wheeze

1. Severe episode ≥ 10 days each month during the last year.

For peer review only - http://bmjopen.bmj.com/site/about/guidelines.xhtml

2. Severe episode ≥ 5 days each month during the last year, or mild episode ≥ 10 days each month during the last year.

3. Severe episode ≥ 1 day each month during the last year, or mild episode ≥ 5 days each month during the last year.

4. Mild episode ≥ 1 day each month during the last year.

5. No episodes of wheezing.

Cough and sputum

1. Daily cough and sputum, with a large amount of sputum or difficulty clearing sputum.

2. Daily cough and sputum, with a moderate amount of sputum or difficulty clearing sputum.

3. Daily cough and sputum, but not troublesome during daily life.

4. Daily cough and sputum for ≤ 3 months each year.

5. No cough or sputum.

Air pollution monitoring

Data on mean daily concentrations of air pollutants were obtained at 21 points in Kurashiki. Monitoring of SO2 concentrations started in 1965, while monitoring of nitrogen dioxide (NO2) concentrations commenced in 1971.

Statistical analysis

Data from patients in each of the four comparison groups were compared using the Kruskal-Wallis test and the Mann-Whitney U test as a post-hoc test. Differences in gender distributions and the prevalence of respiratory diagnoses in each group were analysed using the Cochran-Armitage test, using the chi-square test to test for statistical significance. Changes in lung function between initial evaluation and follow-up were analysed using the Wilcoxon signed-rank test and the Mann-Whitney U test as a post-hoc test. A normality test was performed on all continuous variables using the

For peer review only - http://bmjopen.bmj.com/site/about/guidelines.xhtml

Kolmogorov-Smirnov test before analysis. All analyses were performed using PASW Statistics, version 18. Finally, p-values of <0.05 were considered statistically significant.

RESULTS

Air pollutants

Figure 2 shows the annual mean daily levels of SO_2 and NO_2 recorded from 1965 to 2009 in Kurashiki. The Air Pollution Control Law was enacted in 1968. SO_2 levels were above the acceptable level every year from 1968 to 1974 and then decreased to below 40 parts per billion (ppb), which is the acceptable level defined by the Air Pollution Control Law. In 1973, the acceptable NO_2 level was changed by the Air Pollution Control Law from 20 ppb to 40 ppb. The NO_2 level exceeded the revised acceptable level only in 1973.

Sample characteristics

Table 1 shows the characteristics of our patient sample at initial evaluation. We found significant differences in the age distributions between groups ($p<0.001$). Males outnumbered females in the Group A and Group C ($p<0.001$). Patients in the Group B and Group D had significantly lower heights and weights ($p<0.001$).

We found differences in FVC values between groups, with patients in the Group B and Group D presenting significantly lower values than those in the Group A and Group C ($p<0.001$). FEV_1 values, which also differed significantly between comparison groups ($p<0.001$), were lowest in the Group B, followed by in the Group A. %VC and FEV_1/FVC ratios were lowest the Group A and Group B. Finally, the prevalence of bronchial asthma at initial evaluation was much higher in the Group B compared with all other groups ($p<0.001$).

For peer review only - http://bmjopen.bmj.com/site/about/guidelines.xhtml

For peer review only

Change in respiratory function and symptoms between initial evaluation and follow-up

Table 2 and 3 show the results of our evaluation of the change in severity of respiratory symptoms and respiratory function between initial evaluation and follow-up. Respiratory function had deteriorated in almost all subjects by follow-up. In particular, FEV/FVC ratios in Group C, which averaged 79.1±5.6% at baseline, had fallen to 67.9±10.2% at follow-up because of the development of obstructive ventilatory defects in a number of subjects. Conversely, average FEV/FVC ratios increased from 59.8±10.3% to 62.1±13.4% in Group B ($p<0.001$). The median level of respiratory symptoms worsened from level II to level III in all groups. However, Group D saw a decrease in the proportion of patients reporting asthma attacks ($p=0.78$), cough, and sputum production ($p=0.24$) over the period studied.

Mean annual change in respiratory function

Table 4 shows the mean annual change in respiratory function in each comparison group.

Mean FEV1 decreased to a similar extent in both Group A and Group C. While there was a slight fall in FEV1 in Group D, there was a marginal improvement in FEV1 in Group B ($p<0.001$). At the same time, while FEV1/FVC ratios fell ($p<0.001$) in both Group A and Group C, an improvement was seen in Group B. Finally, %VC fell in all groups ($p<0.001$), although the decline was greater in Group A and Group B than in Group C and Group D, as shown in Table 4.

Comparison of respiratory symptoms at final evaluation

Table 5 shows the severity of respiratory symptoms in our four comparison groups at follow-up.

For peer review only - http://bmjopen.bmj.com/site/about/guidelines.xhtml

In Group A and Group B, whose subjects were diagnosed with obstructive ventilatory defects at baseline, the great majority reported severe dyspnoea, asthma, cough and sputum production. Severe dyspnoea was also highly prevalent in Group A and Group B. While severity of asthma was high in both Group B and Group D at initial evaluation, the proportion of patients reporting severe asthma had only increased significantly in Group A and Group C at follow-up ($p<0.001$). Severity of asthma was similar in Group C and Group D at follow-up. Prevalence of severe cough and sputum production increased significantly in Group A and Group C ($p<0.001$), with 80% of subjects in Group A reporting scores that changed from I to III due to a worsening of symptoms.

DISCUSSION

This retrospective cross-sectional study analysed changes in respiratory function and respiratory symptoms between two points in time (initial evaluation and follow-up) among a group of designated pollution victims in Japan. Our results show for the first time that improvements in respiratory function and reductions in respiratory symptoms are prevented by continuing tobacco use.

Among those patients diagnosed with an obstructive impairment due to air pollution exposure (Groups A and B), we found that FEV1/FVC ratios had deteriorated by follow-up with no reduction in morbidity. However, our results showed an improvement in FEV1/FVC ratios in Group B, which could be attributed to the high proportion of subjects in this group diagnosed with bronchial asthma at initial evaluation when compared with the other groups.

We observed that respiratory function had deteriorated among patients in Group A despite the improvement in local air quality, resulting in depressed FEV1/FVC ratios. Furthermore, while FEV1/FVC ratios in Group C were relatively low at the time of initial diagnosis, continuing tobacco use had resulted in some cases in obstructive impairment, causing FEV1/FVC ratios at follow-up to

For peer review only - http://bmjopen.bmj.com/site/about/guidelines.xhtml

decrease to 67.9±10.2%.

While air pollution can cause airway obstruction resulting in asthma, reactive airways dysfunction syndrome and reduced lung function, tobacco use, which can result in airway hyper-responsiveness, has been shown to exert a stronger influence on respiratory health in a number of studies.[10 11] These effects were evident in Group A and Group C, which showed similar annual rates of change in respiratory function over time. Conversely, Downs et al. have shown that, in non-smokers, annual rates of change in FEV1 can return to within the normal range in response to an improvement in air quality.[12] These findings are similar to the results of the present study. Previous studies have reported a mean annual rate of change in FEV1 of between -30 and -22 ml/year in healthy, non-smoking males and females aged > 65 years.[13] Patients with COPD had a mean annual change in FEV1 of 30–80 ml/year.[14] However, exposure to air pollution and tobacco smoke, both of which can result in tracheobronchial injury, can worsen annual rates of change in lung function markedly.

A high proportion of our subjects, particularly those with obstructive impairment, reported respiratory symptoms at baseline. These symptoms were more pronounced among the smokers in our sample. In Group D, however, the proportion of patients reporting asthma attacks, sputum production and cough had not increased, suggesting that these symptoms may be prevented by smoking cessation. The proportion of subjects in Group B reporting asthma attacks had increased by follow-up. Furthermore, the proportion of subjects reporting asthma attacks in Group C had increased to the same levels as that found in Group D at initial evaluation. Repeated bronchial inflammation caused by smoking and pollutant exposure can result in thickening of the bronchial wall and airway remodelling that cannot be reversed even by steroid treatment following the onset of hypersensitivity.[15] Furthermore, Siroux et al. have shown that continued tobacco use can significantly increase the incidence of asthma attacks.[16] Because of this association, smoking is listed as a potential trigger for asthma and COPD in asthma management guidelines.[17] Tobacco

For peer review only - http://bmjopen.bmj.com/site/about/guidelines.xhtml

use is also likely to have been the cause of the higher prevalence of severe cough and sputum production found in in Group A and Group C.

A number of studies have shown that smoking can cause respiratory tract inflammation and exacerbate symptoms such as cough and sputum production.[18]This was reflected in our finding that the severity of dyspnoea among subjects in Group A and Group B had increased markedly at follow-up. While a number of causes of dyspnoea have been identified, including age, poor physical fitness, muscle atrophy and social and environmental factors, we could not identify the primary determinants of dyspnoea in the present study.

[19] Finally, progression of respiratory disease and a decline in lung function can further aggravate these symptoms.[20]

In Asia, increased mortality and morbidity resulting from lung cancer, COPD, asthma and heart disorders caused by rising atmospheric PM 2.5 concentrations is a growing public health concern. According to the Organization for Economic Cooperation and Development, air pollution is expected to become the leading cause of death worldwide by 2050, and is likely to be responsible for between three and six million deaths annually.[21] Furthermore, Pope et al. have reported that worldwide pollution-related deaths could rise by 6% for each additional 10 µg/m3 increase in atmospheric PM 2.5 concentrations.[22]

The present study emphasizes the need to prevent or reduce air pollution and to improve public education on the negative health effects of smoking to reduce pulmonary morbidity on the population level. This is especially the case among designated victims, for whom additional education programs, behavioural interventions and support for smoking cessation would be particularly beneficial. One such behavioural intervention that has been proven to be cost-effective in reducing smoking prevalence is the five-step Global Initiative for Obstructive Lung Disease (GOLD) framework (ask, advise, assess, assist, arrange).[23] This strategy, which aims at delaying

For peer review only - http://bmjopen.bmj.com/site/about/guidelines.xhtml

the progress of COPD through continuing support for smoking cessation, may be applicable for use in similarly affected communities.[24]

This study has several limitations. First, information on number of cigarettes smoked and individual-level exposure to environmental pollutants was not available. Second, it was not within the scope of the present study to explore the role of gender-specific differences in exposure to tobacco smoke and pollutants – much of which may have been occupational. Third, we were unable to clearly distinguish between the effects of mainstream and side-stream cigarette smoke when considering the health effects of air pollution. Finally, we were unable to account for possible confounding factors such as socio-economic class and lifestyle factors, which may have resulted in individual-level differences in exposure to environmental pollutants.

Further work is needed to inform future prevention and treatment programs to improve the quality-of-life of the remaining designated pollution victims in Japan. The results of the present study are of particular importance in the context of high rates of smoking and worsening air quality in a number of Asian countries as they undergo rapid economic change.

Acknowledgements

We thank the study participants, technical staff, administrative support team, and our co-workers for their help. We thank the Environmental Restoration and Conservation Agency and Mizushima-Kyodo Hospital for their support.

Funding

This work was supported by the Environmental Restoration and Conservation Agency and Mizushima-Kyodo Hospital.

Competing interests

For peer review only

The authors declare that they have no competing interests.

Contributions

HS was the principal investigator and contributed to the design of the study, administered the funding, supervised the team's work and made critical revisions to the manuscript for intellectual content. KK designed the study, collected data, analysed and interpreted data, and prepared the manuscript. SH took part in analysing and interpreting the data, and made critical revisions to the manuscript for intellectual content. All other authors took part in data collection and interpretation, and have read and approved the final manuscript.

Data sharing statement:

Additional unpublished data from the study are available from TT (tanakataka@nagasaki-u.ac.jp) and YY (dependonhero@yahoo.co.jp). They can be reached by email.

For peer review only

Table 1. Demographics and respiratory characteristics of comparison groups at initial evaluation for designation of air pollution victim status

		Group A (n=48)	Group B (n=169)	Group C (n=119)	Group D (n=394)	p	post-hoc test
Age		54.3 ± 8.3	49.6 ± 8.2	49.8 ± 7.8	51.3 ± 8.9	<.001	b.c.f
Gender (male/female)		35/13	66/103	81/38	90/304	<.001	a.b.c.d.e.f
Height	(cm)	161.7 ± 6.3	155.2 ± 7.6	161.4 ± 6.1	152.8 ± 7.5	<.001	a.b.d.e.f
Weight	(kg)	56.9 ± 6.4	55.4 ± 9.3	59.1 ± 8.9	55.2 ± 9.7	<.001	b.f
BMI		21.8 ± 1.9	22.8 ± 3.6	22.6 ± 2.8	23.4 ± 3.8	<.001	a.b.d.e.
FVC	(l)	3.37 ± 0.79	3.04 ± 0.79	3.49 ± 0.76	2.94 ± 0.68	<.001	a.b.e.f
%VC	(%)	105.5 ± 18.7	103.5 ± 18.5	108.3 ± 15.5	107.6 ± 17.3	<.05	d.f
FEV1	(l)	2.09 ± 0.64	1.72 ± 0.58	2.67 ± 0.58	2.24 ± 0.58	<.001	a.b.c.d.f
FEV1/FVC	(%)	64.9 ± 6.5	59.8 ± 10.3	79.1 ± 5.6	79.6 ± 7.1	<.001	a.c.d.e.f
Data of duration after certification	(yr)	25.9±4.1	26.9±4.4	24.8±4.2	25.5±4.6	<.001	d.f
Diagnosed pulmonary disease CB,BA,PE CB+BA CB+PE, BA+PE	no	32/10/1/5/ 0/ 0	52/98/3/11/3/2	98/14/0/7/ 0/ 0	249/99/0/46/0/0	<.001	a.c.d.f
	Unit Score						
	1	0 (0.0%)	1(0.6%)	0 (0.0%)	0 (0.0%)		
	2	0 (0.0%)	4 (2.4%)	0 (0.0%)	4 (1.1%)		
Dyspnea	3	9 (18.8%)	31 (18.3%)	7 (5.9%)	55 (15.8%)	<.001	b.c.d.f
	4	30 (62.5%)	111 (65.7%)	75 (63.0%)	246 (70.5%)		
	5	9 (18.8%)	22 (13.0%)	37 (31.1%)	89 (25.5%)		
	1	0 (0.0%)	1 (0.6%)	0 (0.0%)	1 (0.3%)		
	2	0 (0.0%)	8 (4.7%)	1 (0.8%)	13 (3.3%)		
Asthma attack	3	7 (14.6%)	51 (30.2%)	11 (9.2%)	66 (16.8%)	<.001	a.b.d.f
	4	14 (29.2%)	76 (45.0%)	30 (25.2%)	139 (35.3%)		
	5	27 (56.3%)	33 (19.5%)	77 (64.7%)	175 (44.4%)		
	1	0 (0.0%)	2 (1.2%)	0 (0.0%)	0 (0.0%)		
	2	0 (0.0%)	5 (3.0%)	1 (0.8%)	9 (2.3%)		
Cough and phlegm	3	24 (50.0%)	50 (29.6%)	58 (48.7%)	143 (36.3%)	<.05	a.b.d.f
	4	23 (47.9%)	90 (53.3%)	58 (48.7%)	225 (57.1%)		
	5	1 (2.1%)	22 (13.0%)	1 (0.8%)	15 (3.8%)		

For peer review only

For peer review only - http://bmjopen.bmj.com/site/about/guidelines.xhtml

Figures are presented as mean ±SD

FEV1: Forced Expiratory Volume in 1 second, FVC: Forced Vital Capacity, VC%: % Vital Capacity

FEV1/FVC (%) Forced Expiratory Volume in 1 second.

CB: Chronic Bronchitis BA: Bronchial Asthma PE: Pulmonary Emphysema

CB+BA CB+PE BA+PE

Differences in sample characteristics analysed using the Kruskal-Wallis test with Cochran-Armitage tests for significance

post-hoc tests: Mann-Whitney U test, chi-square test for significance

a: Group A vs Group B

b:Group C vs Group D

c: Group A vs Group C

d: Group B vs Group D

e: Group A vs Group D

f:Group B vs Group C

a, b: Current smokers / ex-smokers (A and C Groups) vs never smokers (B and D Groups)

c, d: Diagnosis of obstructive ventilatory defect (A and B Groups) vs no obstructive ventilatory defect (C and D Groups)

For peer review only

For peer review only - http://bmjopen.bmj.com/site/about/guidelines.xhtml

Table 2. Change in demographic characteristics and respiratory function of designated air pollution victims by comparison group between initial evaluation and follow-up

		Initial evaluation	Follow-up	p	Initial evaluation	Follow-up	p	Initial evaluation	Follow-up	p	Initial evaluation	Follow-up	p
		Group A			Group B			Group C			Group D		
Age		54.3 ± 8.3	78.6 ± 6.5	<.001	49.6 ± 8.2	77.3 ± 7.5	<.001	49.8 ± 7.8	74.2 ± 7.1	<.001	51.3 ± 8.9	76.5 ± 7.8	<.001
Height	(cm)	161.7 ± 6.3	159.3 ± 6.9	<.001	155.2 ± 7.6	150.8 ± 8.8	<.001	161.4 ± 6.1	159.2 ± 6.2	<.001	152.8 ± 7.5	148.9 ± 8.8	<.001
Weight	(kg)	56.9 ± 6.4	55.4 ± 9.2	<.001	55.4 ± 9.3	52.1 ± 10.9	<.001	59.1 ± 8.9	58.9 ± 9.6	<.001	55.2 ± 9.7	53.2 ± 11.3	<.001
BMI		21.8 ± 1.9	21.7 ± 2.9	<.001	22.8 ± 3.6	22.7 ± 3.9	<.001	22.6 ± 2.8	23.2 ± 3.1	<.05	23.4 ± 3.8	23.9 ± 4.2	0.13
PFT													
FVC	(l)	3.37 ± 0.79	2.54 ± 0.85	<.001	3.04 ± 0.79	2.21 ± 0.73	<.001	3.49 ± 0.76	2.78 ± 0.81	<.001	2.94 ± 0.68	2.28 ± 0.69	<.001
%VC	(%)	105.5 ± 18.7	93.1 ± 21.7	<.001	103.5 ± 18.5	90.3 ± 22.3	<.001	108.3 ± 15.5	100.2 ± 21.9	<.001	107 ± 17.3	100.4 ± 23.4	<.001
FEV1	(l)	2.09 ± 0.64	1.28 ± 0.53	<.001	1.72 ± 0.58	1.29 ± 0.51	<.001	2.67 ± 0.58	1.8 ± 0.68	<.001	2.24 ± 0.58	1.61 ± 0.52	<.001
FEV1 /FVC	(%)	64.9 ± 6.5	54.5 ± 2.3	<.001	59.8 ± 10.3	62.1 ± 13.4	<.001	79.1 ± 5.6	67.9 ± 10.2	<.001	79.6 ± 7.1	73.8 ± 13.8	<.001

PFT: Pulmonary Function Testing

Figures are presented as mean ±SD

Initial evaluation versus follow-up: Mann-Whitney U test

For peer review only

For peer review only - http://bmjopen.bmj.com/site/about/guidelines.xhtml

Table 3. Change in respiratory symptoms of designated air pollution victims by comparison group between initial evaluation and follow-up

	Unit Score	Initial evaluation		Follow-up		p	Initial evaluation		Follow-up		p	Initial evaluation		Follow-up		p	Initial evaluation		Follow-up		p
Dyspnoea		Group A					Group B					Group C					Group D				
	1	0	0.0%	1	2.1%		1	0.6%	2	1.2%		0	0.0%	1	0.8%		0	0.0%	0	0.0%	
	2	0	0.0%	4	8.3%		4	2.4%	15	8.9%		0	0.0%	2	1.7%		4	1.1%	13	3.7%	
	3	9	18.8%	24	50.0%	<.001	31	18.3%	74	43.8%	<.001	7	5.9%	20	16.8%	<.001	55	15.8%	67	19.2%	<.001
	4	30	62.5%	15	31.3%		111	65.7%	75	44.4%		75	63.0%	77	64.7%		246	70.5%	279	79.9%	
	5	9	18.8%	4	8.3%		22	13.0%	3	1.8%		37	31.1%	19	16.0%		89	25.5%	35	10.0%	
Asthma attack																					
	1	0	0.0%	0	0.0%		1	0.6%	0	0.0%		0	0.0%	0	0.0%		1	0.3%	0	0.0%	
	2	0	0.0%	3	6.3%		8	4.7%	19	11.2%		1	0.8%	4	3.4%		13	3.3%	10	2.5%	
	3	7	14.6%	16	33.3%	<.001	51	30.2%	72	42.6%	<.001	11	9.2%	17	14.3%	<.001	66	16.8%	71	18.0%	0.78
	4	14	29.2%	15	31.3%		76	45.0%	51	30.2%		30	25.2%	35	29.4%		139	35.3%	139	35.3%	
	5	27	56.3%	14	29.2%		33	19.5%	27	16.0%		77	64.7%	63	52.9%		175	44.4%	174	44.2%	
Cough and phlegm																					
	1	0	0.0%	1	2.1%		2	1.2%	1	0.6%		0	0.0%	0	0.0%		0	0.0%	0	0.0%	
	2	0	0.0%	3	6.3%		5	3.0%	8	4.7%		1	0.8%	2	1.7%		9	2.3%	9	2.3%	
	3	24	50.0%	34	70.8%	<.001	50	29.6%	99	58.6%	<.001	58	48.7%	66	55.5%	<.001	143	36.3%	155	39.3%	0.24
	4	23	47.9%	9	18.8%		90	53.3%	55	32.5%		58	48.7%	49	41.2%		227	57.1%	221	56.1%	
	5	1	2.1%	1	2.1%		22	13.0%	6	3.6%		2	0.8%	2	1.7%		15	3.8%	9	2.3%	

Respiratory symptoms

Numerical values are shown (%).

Initial evaluation versus follow-up: Wilcoxon signed-rank test

For peer review only

For peer review only - http://bmjopen.bmj.com/site/about/guidelines.xhtml

Table 4. Mean annual change in yearly spirometry results from initial evaluation to follow-up by comparison group

		Group A (n=48)	Group B (n=169)	Group C (n=119)	Group D (n=394)	p	post-hoc test
FEV1 decline/year	(ml)	-36.7±19.1	-26.5±18.5	-37.2±16.4	-25.5±17.2	<.001	a.b.d.e.f
FEV1/FVC decline/year	(%)	-0.04±0.05	0.09±0.06	-0.04±0.03	-0.02±0.04	<.001	a.b.d.e.f
FVC decline/year	(ml)	-31.7±24.5	-30.1±20.7	-29.9±22.8	-25.3±17.1	<.05	d
%VC decline/year	(%)	-0.05±0.08	-0.05±0.08	-0.03±0.06	-0.03±0.07	<.001	c.d.e.f

Figures are presented as mean ±SD

p-values were calculated using the Kruskal-Wallis test post-hoc test: Mann-Whitney U test

a: Group A vs Group B

b:Group C vs Group D

c: Group A vs Group C

d: Group B vs Group D

e: Group A vs Group D

f:Group B vs Group C

a, b: Current smokers / ex-smokers (A and C Groups) vs never smokers (B and D Groups)

c, d: Diagnosis of obstructive ventilatory defect (A and B Groups) vs no obstructive ventilatory defect (C and D Groups)

For peer review only

For peer review only - http://bmjopen.bmj.com/site/about/guidelines.xhtml

Table 5. Change in severity of smoking-related respiratory symptoms by comparison group at follow-up

Score	Group A (n=48)		Group B (n=169)		Group C (n=119)		Group D (n=394)		p	post-hoc test
Dyspnea										
1	1	2.1%	2	1.2%	1	0.8%	0	0.0%		
2	4	8.3%	15	8.9%	2	1.7%	13	3.7%		
3	24	50.0%	74	43.8%	20	16.8%	67	19.2%	<.001	c.d.e.f
4	15	31.3%	75	44.4%	77	64.7%	279	79.9%		
5	4	8.3%	3	1.8%	19	16.0%	35	10.0%		
Asthma attack										
1	0	0.0%	0	0.0%	0	0.0%	0	0.0%		
2	3	6.3%	19	11.2%	4	3.4%	10	2.5%		
3	16	33.3%	72	42.6%	17	14.3%	71	18.0%	<.001	a.c.d.e.f
4	15	31.3%	51	30.2%	35	29.4%	139	35.3%		
5	14	29.2%	27	16.0%	63	52.9%	174	44.2%		
Cough and phlegm										
1	1	2.1%	1	0.6%	0	0.0%	0	0.0%		
2	3	6.3%	8	4.7%	2	1.7%	9	2.3%		
3	34	70.8%	99	58.6%	66	55.5%	155	39.3%	<.001	a.b.c.d.e.
4	9	18.8%	55	32.5%	49	41.2%	221	56.1%		
5	1	2.1%	6	3.6%	2	1.7%	9	2.3%		

Numerical values are shown (%)

p-values were calculated using the Kruskal-Wallis test post-hoc test: Mann–Whitney U test

a: Group A vs Group B

b:Group C vs Group D

c: Group A vs Group C

d: Group B vs Group D

e: Group A vs Group D

f:Group B vs Group C

a, b: Current smokers / ex-smokers (A and C Groups) vs never smokers (B and D Groups)

c, d: Diagnosis of obstructive ventilatory defect (A and B Groups) vs no obstructive ventilatory defect (C and D Groups)

For peer review only

REFERENCES

1. Yang G, Wang Y, Zeng Y, et al. Rapid health transition in China, 1990-2010: findings from the Global Burden of Disease Study 2010. Lancet 2013;**381**(9882):1987-2015 doi: 10.1016/S0140-6736(13)61097-1
S0140-6736(13)61097-1 [pii][published Online First: Epub Date]|.
2. Liu Y, Lee K, Perez-Padilla R, et al. Outdoor and indoor air pollution and COPD-related diseases in high- and low-income countries. Int J Tuberc Lung Dis 2008;**12**(2):115-27
3. Yang JY, Xin JY, Ji DS, et al. [Variation analysis of background atmospheric pollutants in North China during the summer of 2008 to 2011]. Huan Jing Ke Xue 2012;**33**(11):3693-704
4. Tanaka T, Asai M, Yanagita Y, et al. Longitudinal study of respiratory function and symptoms in a non-smoking group of long-term officially-acknowledged victims of pollution-related illness. BMC Public Health 2013;**13**:766 doi: 10.1186/1471-2458-13-766
1471-2458-13-766 [pii][published Online First: Epub Date]|.
5. Usmanova G, Mokdad AH. Results of the Global Youth Tobacco Survey and implementation of the WHO Framework Convention on Tobacco Control in the WHO Eastern Mediterranean Region (EMR) countries. J Epidemiol Glob Health 2013;**3**(4):217-34 doi: 10.1016/j.jegh.2013.07.001
S2210-6006(13)00084-1 [pii][published Online First: Epub Date]|.
6. Yang G, Fan L, Tan J, et al. Smoking in China: findings of the 1996 National Prevalence Survey. JAMA 1999;**282**(13):1247-53 doi: joc80784 [pii][published Online First: Epub Date]|.
7. M C. WHO report on the global tobacco epidemic 2008. Geneva:World Health Organization 2008
8. Yamagata T, Hirano T, Sugiura H, et al. Comparison of bronchodilatory properties of transdermal and inhaled long-acting beta 2-agonists. Pulm Pharmacol Ther 2008;**21**(1):160-5 doi: S1094-5539(07)00047-8 [pii]
10.1016/j.pupt.2007.05.004[published Online First: Epub Date]|.
9. Baldwin ED CA, Richards DW Jr. Pulmonary insufficiency; physiological classification, clinical methods of analysis, standard values in normal subjects. Medicine (Baltimore) 1948; **27(3)**:243-78
10. Curjuric I, Imboden M, Nadif R, et al. Different genes interact with particulate matter and tobacco smoke exposure in affecting lung function decline in the general population. PLoS One 2012;**7**(7):e40175 doi: 10.1371/journal.pone.0040175
PONE-D-11-21280 [pii][published Online First: Epub Date]|.
11. Ceylan E, Kocyigit A, Gencer M, et al. Jul 2006 patent 0954-6111 (Print)
0954-6111 (Linking).
12. Downs SH, Schindler C, Liu LJ, et al. Reduced exposure to PM10 and attenuated age-related decline in lung function. N Engl J Med 2007;**357**(23):2338-47 doi: 357/23/2338 [pii]
10.1056/NEJMoa073625[published Online First: Epub Date]|.
13. Lee PN, Fry JS. Systematic review of the evidence relating FEV1 decline to giving up smoking. BMC Med 2010;**8**:84 doi: 10.1186/1741-7015-8-84
1741-7015-8-84 [pii][published Online First: Epub Date]|.
14. Fletcher C, Peto R. The natural history of chronic airflow obstruction. British medical journal 1977;**1**(6077):1645-8
15. Downs SH, Schindler C, Liu LJ, et al. Reduced exposure to PM10 and attenuated age-related decline in lung function. N Engl J Med 2007;**357**(23):2338-47 doi: 10.1056/NEJMoa073625[published Online First: Epub Date]|.
16. Siroux V, Pin I, Oryszczyn MP, et al. Relationships of active smoking to asthma and asthma severity in the EGEA study. Epidemiological study on the Genetics and Environment of Asthma. Eur Respir J 2000;**15**(3):470-7

For peer review only - http://bmjopen.bmj.com/site/about/guidelines.xhtml

17. Gruffydd-Jones K. GOLD guidelines 2011: what are the implications for primary care? Primary care respiratory journal : journal of the General Practice Airways Group 2012;**21**(4):437-41 doi: 10.4104/pcrj.2012.00058[published Online First: Epub Date]|.

18. Pelkonen M, Notkola IL, Nissinen A, et al. Thirty-year cumulative incidence of chronic bronchitis and COPD in relation to 30-year pulmonary function and 40-year mortality: a follow-up in middle-aged rural men. Chest 2006;**130**(4):1129-37 doi: 130/4/1129 [pii]
10.1378/chest.130.4.1129[published Online First: Epub Date]|.

19. Berglund DJ, Abbey DE, Lebowitz MD, et al. Respiratory symptoms and pulmonary function in an elderly nonsmoking population. Chest 1999;**115**(1):49-59

20. Oga T TM, Hajiro T, Ikeda A, Nishimura K. Analysis of longitudinal changes in dyspnea of patients with chronic obstructive pulmonary disease: an observational study. Respir Res 2012;**13**:85

21. Dong GH, Zhang P, Sun B, et al. Long-term exposure to ambient air pollution and respiratory disease mortality in Shenyang, China: a 12-year population-based retrospective cohort study. Respiration; international review of thoracic diseases 2012;**84**(5):360-8 doi: 10.1159/000332930[published Online First: Epub Date]|.

22. C. Arden Pope I caRTB, Michelle C. Turner, Aaron Cohen, Daniel Krewski, Michael Jerrett, Susan M. Gapstur, and Michael J. Thun. Lung Cancer and Cardiovascular Disease Mortality Associated with Ambient Air Pollution and Cigarette Smoke: Shape of the Exposure–Response Relationships. Environ Health Perspect 2011
119(11): 1616–21

23. Kaufman G. Chronic obstructive pulmonary disease: diagnosis and management. Nurs Stand 2013;**27**(21):53-7, 60-2

24. website.http GIfCOLDG, //www.goldcopd.org/. Global strategy for the diagnosisGlobal strategy for the diagnosis. management and prevention of COPD, 2011.

For peer review only

For peer review only - http://bmjopen.bmj.com/site/about/guidelines.xhtml

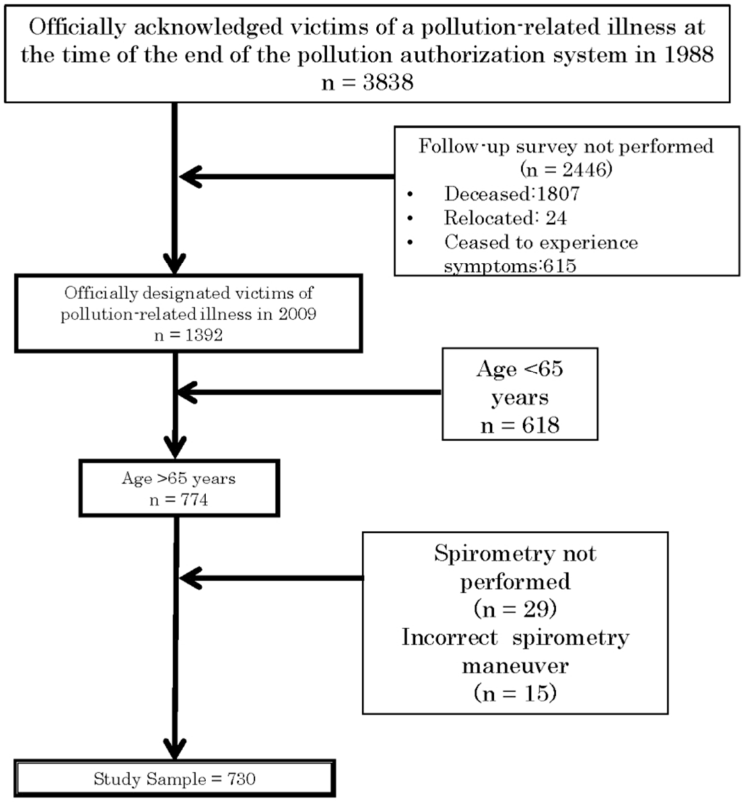

Figure 1: Flowchart showing the selection procedure for study subjects.
The study included officially designated victims of pollution-related illness in Kurashiki aged 65 years or older in 2009, and for whom full data were available for statistical analysis.
Ceased to experience symptoms: Officially designated victims who did renew the designation because of disappearance of clinical symptoms.

90x119mm (300 x 300 DPI)

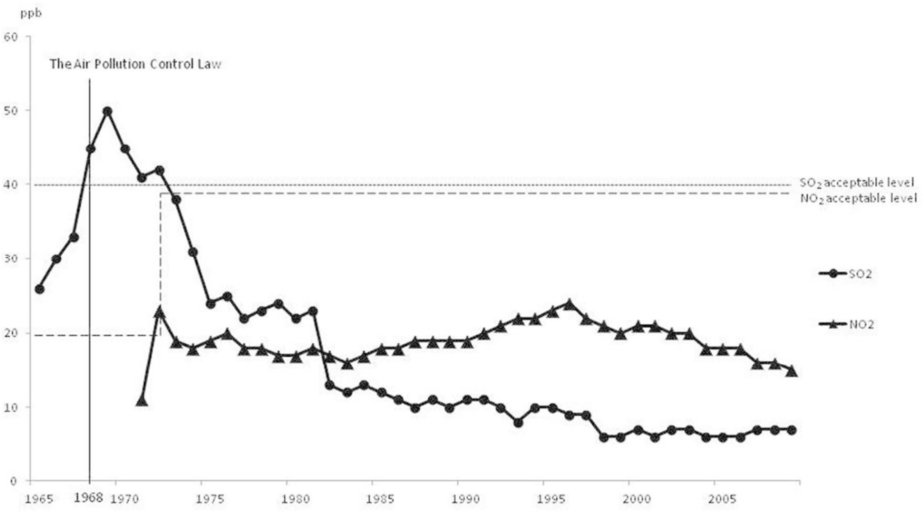

Figure 2 levels of air pollution

90x67mm (300 x 300 DPI)

STROBE 2007 (v4) Statement—Checklist of items that should be included in reports of *cohort studies*

Section/Topic	Item #	Recommendation	Reported on page #
**Title and abstract**	1	(*a*) Indicate the study's design with a commonly used term in the title or the abstract	
		(*b*) Provide in the abstract an informative and balanced summary of what was done and what was found	
**Introduction**			
Background/rationale	2	Explain the scientific background and rationale for the investigation being reported	
Objectives	3	State specific objectives, including any prespecified hypotheses	
**Methods**			
Study design	4	Present key elements of study design early in the paper	
Setting	5	Describe the setting, locations, and relevant dates, including periods of recruitment, exposure, follow-up, and data collection	
Participants	6	(*a*) Give the eligibility criteria, and the sources and methods of selection of participants. Describe methods of follow-up	
		(*b*) For matched studies, give matching criteria and number of exposed and unexposed	
Variables	7	Clearly define all outcomes, exposures, predictors, potential confounders, and effect modifiers. Give diagnostic criteria, if applicable	
Data sources/ measurement	8*	For each variable of interest, give sources of data and details of methods of assessment (measurement). Describe comparability of assessment methods if there is more than one group	
Bias	9	Describe any efforts to address potential sources of bias	
Study size	10	Explain how the study size was arrived at	
Quantitative variables	11	Explain how quantitative variables were handled in the analyses. If applicable, describe which groupings were chosen and why	
Statistical methods	12	(*a*) Describe all statistical methods, including those used to control for confounding	
		(*b*) Describe any methods used to examine subgroups and interactions	
		(*c*) Explain how missing data were addressed	
		(*d*) If applicable, explain how loss to follow-up was addressed	
		(*e*) Describe any sensitivity analyses	
**Results**			

For peer review only

Participants	13*	(a) Report numbers of individuals at each stage of study—eg numbers potentially eligible, examined for eligibility, confirmed eligible, included in the study, completing follow-up, and analysed	
		(b) Give reasons for non-participation at each stage	
		(c) Consider use of a flow diagram	
Descriptive data	14*	(a) Give characteristics of study participants (eg demographic, clinical, social) and information on exposures and potential confounders	
		(b) Indicate number of participants with missing data for each variable of interest	
		(c) Summarise follow-up time (eg, average and total amount)	
Outcome data	15*	Report numbers of outcome events or summary measures over time	
Main results	16	(*a*) Give unadjusted estimates and, if applicable, confounder-adjusted estimates and their precision (eg, 95% confidence interval). Make clear which confounders were adjusted for and why they were included	
		(*b*) Report category boundaries when continuous variables were categorized	
		(*c*) If relevant, consider translating estimates of relative risk into absolute risk for a meaningful time period	
Other analyses	17	Report other analyses done—eg analyses of subgroups and interactions, and sensitivity analyses	
**Discussion**			
Key results	18	Summarise key results with reference to study objectives	
**Limitations**			
Interpretation	20	Give a cautious overall interpretation of results considering objectives, limitations, multiplicity of analyses, results from similar studies, and other relevant evidence	
Generalisability	21	Discuss the generalisability (external validity) of the study results	
**Other information**			
Funding	22	Give the source of funding and the role of the funders for the present study and, if applicable, for the original study on which the present article is based	

*Give information separately for cases and controls in case-control studies and, if applicable, for exposed and unexposed groups in cohort and cross-sectional studies.

Note: An Explanation and Elaboration article discusses each checklist item and gives methodological background and published examples of transparent reporting. The STROBE checklist is best used in conjunction with this article (freely available on the Web sites of PLoS Medicine at http://www.plosmedicine.org/, Annals of Internal Medicine at http://www.annals.org/, and Epidemiology at http://www.epidem.com/). Information on the STROBE Initiative is available at www.strobe-statement.org.

For peer review only

BMJ Open

Tobacco use among designated air pollution victims and its association with lung function and respiratory symptoms: a retrospective cross-sectional study

Journal:	*BMJ Open*
Manuscript ID:	bmjopen-2014-005393.R3
Article Type:	Research
Date Submitted by the Author:	02-Jul-2014
Complete List of Authors:	kotaki, kenji; Nagasaki University Graduate School of Biomedical Sciences, Course of Cardiopulmonary Rehabilitation Science<br>Senjyu, Hideaki; Nagasaki University Graduate School of Biomedical Science, Course of Cardiopulmonary Rehabilitation Science<br>Tanaka, Takako; Nagasaki University Graduate School of Biomedical Science, Course of Cardiopulmonary Rehabilitation Science<br>Yano, Yudai; Nagasaki University Graduate School of Biomedical Science, Course of Cardiopulmonary Rehabilitation Science<br>Miyamoto, Naomi; Kio University Faculty of Health Science, Department of Physical Therapy<br>Nishinakagawa, Tsuyoshi; Nagasaki University Graduate School of Biomedical Science, Course of Cardiopulmonary Rehabilitation Science<br>Yanagita, Yorihide; Seirei Mikatahara General Hospital, Department of Rehabilitation Medicine<br>Asai, Masaharu; Nagasaki University Graduate School of Biomedical Science, Course of Cardiopulmonary Rehabilitation Science<br>Kozu, Ryo; Nagasaki University Hospital, Department of Rehabilitation Medicine<br>Tabusadani, Mitsuru; Nagasaki University, Center for Industry,University and Government Cooperation<br>Sawai, Terumitsu; Nagasaki University Graduate School of Biomedical Science, Course of Cardiopulmonary Rehabilitation Science<br>Honda, Sumihisa; Nagasaki University Graduate School of Biomedical Science, Course of Cardiopulmonary Rehabilitation Science
**Primary Subject Heading**:	Smoking and tobacco
Secondary Subject Heading:	Epidemiology, Respiratory medicine
Keywords:	EPIDEMIOLOGY, SOCIAL MEDICINE, Health & safety < HEALTH SERVICES ADMINISTRATION & MANAGEMENT, Public health < INFECTIOUS DISEASES, Chronic airways disease < THORACIC MEDICINE, PUBLIC HEALTH

SCHOLARONE™
Manuscripts

For peer review only

Title

Tobacco use among designated air pollution victims and its association with lung function and respiratory symptoms: a retrospective cross-sectional study

Authors

Kenji Kotaki, Hideaki Senjyu*, Takako Tanaka, Yudai Yano, Naomi Miyamoto, Tsuyoshi Nishinakagawa, Yorihide Yanagita, Masaharu Asai, Ryo Kozu, Mitsuru Tabusadani, Terumitsu Sawai, Sumihisa Honda

Author details

Department of Cardiopulmonary Rehabilitation Science, Nagasaki University Graduate School of Biomedical Sciences, Nagasaki, Japan

***Corresponding author**

Department of Cardiopulmonary Rehabilitation Science

Nagasaki University Graduate School of Biomedical Sciences

1-7-1 Sakamoto, Nagasaki 852-8520, Japan

Email: senjyu@nagasaki-u.ac.jp

Tel: +81-95-819-7963; Fax: +81-95-819-7963

Keywords: Air pollution, Tobacco, Respiratory function, Respiratory symptoms, Pulmonary function

Word count: 3747 words

For peer review only

ABSTRACT

Objectives: We sought to elucidate the long-term association of tobacco use and respiratory health in designated pollution victims with and without obstructive pulmonary defects.

Design: A retrospective cross-sectional study.

Setting: The register of pollution victims in Kurashiki, Japan.

Participants: 730 individuals over 65 years of age previously diagnosed with pollution-related respiratory disease. Patients were classified into four groups according to their smoking status and whether or not they had obstructive pulmonary disease. We then compared the prevalence of respiratory symptoms and lung function over time between groups.

Primary outcome measures: Spirometry was performed and a respiratory health questionnaire completed in the same season each year for up to 30 years.

Results: Rates of smoking and respiratory disease were high in our sample. While respiratory function in non-smoking patients did not completely recover, the annual rate of change in lung function was within the normal range ($p < 0.01$). However, smokers had worse lung function and were more likely to report more severe pulmonary symptoms ($p < 0.01$).

Conclusions: Patients' respiratory function did not fully recover despite improved air quality. Our results suggest that, in the context of exposure to air pollution, tobacco use causes additional loss of lung function and exacerbates respiratory symptoms.

Article summary

Strengths and limitations

- This first study to have analysed changes in respiratory function and respiratory symptoms in a group of designated pollution victims in Japan over up to 30 years.
- Tobacco use caused additional loss of lung function and exacerbated respiratory symptoms in those who have been exposed to air pollution.
- We did not have data on the quantity of cigarettes smoked, the duration of cigarette smoking, or individual-level exposure to environmental pollutants.

For peer review only - http://bmjopen.bmj.com/site/about/guidelines.xhtml

- We were unable to account for possible confounding factors such as socio-economic class and other lifestyle factors.

For peer review only

For peer review only - http://bmjopen.bmj.com/site/about/guidelines.xhtml

INTRODUCTION

In many Asian nations, particularly China, air pollution is increasingly being recognised as an important emerging environmental and public health issue. Previous work has shown that levels of ambient air pollution are associated with an elevated incidence of respiratory diseases and reported prevalence of respiratory symptoms,[1] particularly asthma, pulmonary emphysema and chronic bronchitis.[2] Atmospheric pollution caused by particulate matter less than 2.5 μm in diameter (PM2.5) is reported to be particularly acute in China and in surrounding countries as a result of wind dispersal. This is a growing public health issue in the region, especially given that PM 2.5 is capable of penetrating deep into lung tissue and precipitating a number of respiratory diseases.[3]

Japan was also severely affected during its period of accelerated economic growth in the wake of post-war reconstruction in the 1960s, with both air pollution and the prevalence of respiratory diseases rising markedly. In Kurashiki, a city located in Okayama Prefecture, reports of pulmonary toxicity owing to oxidant air pollutants rose by a factor of 1.73 among the city's inhabitants in the period leading up to 1970 as yearly average SO3 levels increased. The majority of excess pollution-related deaths in this period were caused by acute exacerbation of asthma.

Although patients do not commonly recover fully from pollution-related respiratory diseases, FEV1/ FVC ratios (calculated by dividing Forced Expiratory Volume exhaled at the end of the first second of forced expiration by Forced Vital Capacity) may return to within the normal range once air quality is improved.[4] As a result, Japan passed the Pollution-Related Health Damage Compensation Law in 1973 with the aim of identifying designated air pollution victims within affected areas for compensation. Patients with a diagnosis of bronchial asthma, chronic bronchitis or pulmonary emphysema who had been resident in any designated air pollution zone for a specified period of time were legally recognised as pollution victims. These designated victims were fully reimbursed the costs of all related medical treatment by the state. However, patients' lung function, as measured

For peer review only - http://bmjopen.bmj.com/site/about/guidelines.xhtml

using FEV1/FVC ratios, was not among the diagnostic criteria used to determine eligibility for compensation, as no agreed clinical definition for Chronic Obstructive Pulmonary Disease (COPD) existed at that time.

Tobacco smoke has been shown to contain high levels of PM 2.5, and is likely to be associated with many health issues in common with air pollution. Tobacco use has been shown to reduce lung function, as measured using FEV1/ FVC ratios, and has been identified as a cause of COPD and other tobacco-related diseases. The World Health Organization (WHO) Framework Convention on Tobacco Control was therefore adopted in 2003.[5] However, tobacco use remains a significant public health issue. According to a WHO report, more than 1.1 billion people globally are regular smokers.[6] Furthermore, Asia accounts for a third of total world cigarette consumption, and there has been a local rise in air pollution. [7]

Both air pollution and tobacco smoke have been shown to reduce patients' FEV1/FVC ratios, to increase the risk of obstructive ventilatory defects, and to exacerbate existing respiratory symptoms. Tanaka et al. have reported that when air quality is improved, the annual change in patients' FEV1 can return to within the normal range.[4] However, the association of ongoing tobacco use with respiratory function and pulmonary symptoms in patients with already reduced FEV1/FVC ratios resulting from bronchial hypersensitivity due to air pollution has not yet been elucidated.

The purpose of the present study, carried out among a population of confirmed pollution victims living in a designated pollution-affected area, was to determine the associations between tobacco use and measures of respiratory function and prevalence of pulmonary symptoms up to 30 years after an improvement in air quality. In particular, we sought to compare these associations in patients with and without diagnosed obstructive ventilatory defects resulting from exposure to air pollution.

For peer review only - http://bmjopen.bmj.com/site/about/guidelines.xhtml

METHODS

Study design and setting

The present study was embedded in a retrospective cross-sectional analysis of lung function and respiratory symptoms in designated victims of pollution-related illness in Kurashiki between their initial evaluation (from 1976 to 1988) and follow-up in 2009. Study subjects were drawn from the register of pollution victims in Kurashiki. All study participants met the following conditions, as determined by the Public Relief System of Kurashiki City, in accordance with the Pollution-Related Health Damage Special Measures Law (1969) and the Pollution-Related Health Damage Compensation Law (1973): (1) they had resided or were employed within a designated air pollution zone, and (2) they were diagnosed with chronic bronchitis, asthma, or emphysema by a medical practitioner.

In accordance with the Public Nuisance Countermeasures Law (1967), registered victims were entitled to various forms of compensation including a monthly consultation with a doctor, yearly assessments of respiratory symptoms consisting of a detailed questionnaire and spirometry tests. These patients received treatment with inhaled corticosteroids and long-acting β2 agonists and expectorants, which may have caused improvements in lung function.[8] However, this study did not collect detailed data regarding treatment regimen, and we were therefore unable to evaluate the association between tobacco use and treatment.

Of 419,203 total residents (204,958 males, 214,245 females), 3,838 (0.9%) were officially classified as designated pollution victims in the period up to 1988. The records of these 3,838 residents were reviewed in 2009 with the permission of the Kurashiki City Public Office (Figure 1). Of the 1,392 remaining ‘officially designated victims of pollution-related illness’, we screened 774 individuals aged ≥ 65 years for inclusion in the study, to focus the study on elderly people. The elderly people

For peer review only - http://bmjopen.bmj.com/site/about/guidelines.xhtml

have a higher risk factor for the onset of COPD and show remarkably more respiratory symptoms of COPD, as we restricted to the patients over 65 years in this study. After excluding 44 records that did not have complete spirometry data, the resulting sample size was 730.

We classified patients into two groups according to whether they had an obstructive impairment using the FEV1/FVC ratios recorded at their initial evaluation. Participants in each group were then further sub-divided according to whether they had ever smoked or not(current smoker or ex-smoker or ever smoked) – determined using self-reporting at follow-up, but we could not identify smoking period. This resulted in four comparison groups, labelled as Group A (n=48, current smokers and ex-smokers diagnosed with an obstructive impairment reporting), Group B (n=169, non-smokers diagnosed with an obstructive impairment), Group C (n=119, smokers and ex-smokers without a diagnosed obstructive impairment) and Group D (n=394, non-smokers without an obstructive impairment). The majority of these patients were diagnosed with chronic bronchitis (n = 528, 68.2%), asthma (n = 242, 31.3%), or emphysema (n = 4, 0.5%), as described in case reports provided by authorized medical practitioners.

The study protocol was approved by the Ethical Committee of the Nagasaki University Graduate School of Biomedical Sciences.

Clinical measurements

Patients' gender, height, weight, Body Mass Index (BMI), FVC, Vital Capacity (%VC), FEV1, FEV1/ FVC ratios, diagnosed respiratory conditions and respiratory symptoms were all recorded at baseline and at follow-up.

Spirometry measurements

Spirometry was performed by trained staff at the Mizushima-Kyodo Hospital using an electronic

For peer review only - http://bmjopen.bmj.com/site/about/guidelines.xhtml

spirometry device (FUDAC 70, Fukuda Sangyo Inc., Chiba, Japan). Tests were performed on patients in the sitting position, and were repeated until at least three reproducible forced expiratory curves had been obtained. Both FEV1 and FVC were measured and used to calculate FEV1/FVC ratios in addition to %VC using the method proposed by Baldwin et al. [9]

Annual change of spirometric data

The mean annual change in spirometry data was calculated by subtracting the follow-up results from the initial evaluation results and dividing by the number of years between initial evaluation and follow-up.

Respiratory symptoms

Respiratory symptoms (including dyspnoea, asthma attacks, cough, and sputum production) were assessed by physicians in the same season each year using a respiratory health questionnaire designed by the government of Japan. Each symptom was graded using a standardised five-point scale.

Dyspnoea

1. Too breathless to leave the house, or breathless when dressing or undressing.
2. Breathless after walking about 50 m or after a few minutes on level ground.
3. Breathless when walking on level ground and keeping up with people of the same age, but not breathless when walking at own pace.
4. Breathless when walking up a slight hill or the stairs.
5. Breathless only during strenuous exercise.

Wheeze

1. Severe episode ≥ 10 days each month during the last year.

For peer review only - http://bmjopen.bmj.com/site/about/guidelines.xhtml

2. Severe episode ≥ 5 days each month during the last year, or mild episode ≥ 10 days each month during the last year.

3. Severe episode ≥ 1 day each month during the last year, or mild episode ≥ 5 days each month during the last year.

4. Mild episode ≥ 1 day each month during the last year.

5. No episodes of wheezing.

Cough and sputum

1. Daily cough and sputum, with a large amount of sputum or difficulty clearing sputum.

2. Daily cough and sputum, with a moderate amount of sputum or difficulty clearing sputum.

3. Daily cough and sputum, but not troublesome during daily life.

4. Daily cough and sputum for ≤ 3 months each year.

5. No cough or sputum.

Air pollution monitoring

Data on mean daily concentrations of air pollutants were obtained at 21 points in Kurashiki. Monitoring of SO2 concentrations started in 1965, while monitoring of nitrogen dioxide (NO2) concentrations commenced in 1971.

Statistical analysis

Data from patients in each of the four comparison groups were compared using the Kruskal-Wallis test and the Mann-Whitney U test as a post-hoc test. Differences in gender distributions and the prevalence of respiratory diagnoses in each group were analysed using the Cochran-Armitage test, using the chi-square test to test for statistical significance. Changes in lung function between initial evaluation and follow-up were analysed using the Wilcoxon signed-rank test and the Mann-Whitney U test as a post-hoc test. A normality test was performed on all continuous variables using the

For peer review only

For peer review only - http://bmjopen.bmj.com/site/about/guidelines.xhtml

Kolmogorov-Smirnov test before analysis. All analyses were performed using PASW Statistics, version 18. Finally, p-values of <0.05 were considered statistically significant.

RESULTS

Air pollutants

Figure 2 shows the annual mean daily levels of SO_2 and NO_2 recorded from 1965 to 2009 in Kurashiki. The Air Pollution Control Law was enacted in 1968. SO_2 levels were above the acceptable level every year from 1968 to 1974 and then decreased to below 40 parts per billion (ppb), which is the acceptable level defined by the Air Pollution Control Law. In 1973, the acceptable NO_2 level was changed by the Air Pollution Control Law from 20 ppb to 40 ppb. The NO_2 level exceeded the revised acceptable level only in 1973.

Sample characteristics

Table 1 shows the characteristics of our patient sample at initial evaluation. We found significant differences in the age distributions between groups ($p<0.001$). Males outnumbered females in the Group A and Group C ($p<0.001$). Patients in the Group B and Group D had significantly lower heights and weights ($p<0.001$).

We found differences in FVC values between groups, with patients in the Group B and Group D presenting significantly lower values than those in the Group A and Group C ($p<0.001$). FEV_1 values, which also differed significantly between comparison groups ($p<0.001$), were lowest in the Group B, followed by in the Group A. %VC and FEV_1/FVC ratios were lowest the Group A and Group B. Finally, the prevalence of bronchial asthma at initial evaluation was much higher in the Group B compared with all other groups ($p<0.001$).

For peer review only

For peer review only - http://bmjopen.bmj.com/site/about/guidelines.xhtml

Change in respiratory function and symptoms between initial evaluation and follow-up

Table 2 and 3 show the results of our evaluation of the change in severity of respiratory symptoms and respiratory function between initial evaluation and follow-up. Respiratory function had deteriorated in almost all subjects by follow-up. In particular, FEV/FVC ratios in Group C, which averaged 79.1±5.6% at baseline, had fallen to 67.9±10.2% at follow-up because of the development of obstructive ventilatory defects in a number of subjects. Conversely, average FEV/FVC ratios increased from 59.8±10.3% to 62.1±13.4% in Group B ($p<0.001$). The median level of respiratory symptoms worsened from level II to level III in all groups. However, Group D saw a decrease in the proportion of patients reporting asthma attacks ($p=0.78$), cough, and sputum production ($p=0.24$) over the period studied.

Mean annual change in respiratory function

Table 4 shows the mean annual change in respiratory function in each comparison group.

Mean FEV1 decreased to a similar extent in both Group A and Group C. While there was a slight fall in FEV1 in Group D, there was a marginal improvement in FEV1 in Group B ($p<0.001$). At the same time, while FEV1/FVC ratios fell ($p<0.001$) in both Group A and Group C, an improvement was seen in Group B. Finally, %VC fell in all groups ($p<0.001$), although the decline was greater in Group A and Group B than in Group C and Group D, as shown in Table 4.

Comparison of respiratory symptoms at final evaluation

Table 5 shows the severity of respiratory symptoms in our four comparison groups at follow-up.

For peer review only - http://bmjopen.bmj.com/site/about/guidelines.xhtml

In Group A and Group B, whose subjects were diagnosed with obstructive ventilatory defects at baseline, the great majority reported severe dyspnoea, asthma, cough and sputum production. Severe dyspnoea was also highly prevalent in Group A and Group B. While severity of asthma was high in both Group B and Group D at initial evaluation, the proportion of patients reporting severe asthma had only increased significantly in Group A and Group C at follow-up ($p<0.001$). Severity of asthma was similar in Group C and Group D at follow-up. Prevalence of severe cough and sputum production increased significantly in Group A and Group C ($p<0.001$), with 80% of subjects in Group A reporting scores that changed from I to III due to a worsening of symptoms.

DISCUSSION

This retrospective cross-sectional study analysed changes in respiratory function and respiratory symptoms between two points in time (initial evaluation and follow-up) among a group of designated pollution victims in Japan. Our results show for the first time that improvements in respiratory function and reductions in respiratory symptoms are prevented by continuing tobacco use.

Among those patients diagnosed with an obstructive impairment due to air pollution exposure (Groups A and B), we found that FEV1/FVC ratios had deteriorated by follow-up with no reduction in morbidity. However, our results showed an improvement in FEV1/FVC ratios in Group B, which could be attributed to the high proportion of subjects in this group diagnosed with bronchial asthma at initial evaluation when compared with the other groups.

We observed that respiratory function had deteriorated among patients in Group A despite the improvement in local air quality, resulting in depressed FEV1/FVC ratios. Furthermore, while FEV1/FVC ratios in Group C were relatively low at the time of initial diagnosis, continuing tobacco use had resulted in some cases in obstructive impairment, causing FEV1/FVC ratios at follow-up to

For peer review only - http://bmjopen.bmj.com/site/about/guidelines.xhtml

decrease to 67.9±10.2%.

While air pollution can cause airway obstruction resulting in asthma, reactive airways dysfunction syndrome and reduced lung function, tobacco use, which can result in airway hyper-responsiveness, has been shown to exert a stronger influence on respiratory health in a number of studies.[10 11] These associations were evident in Group A and Group C, which showed similar annual rates of change in respiratory function over time. Conversely, Downs et al. have shown that, in non-smokers, annual rates of change in FEV1 can return to within the normal range in response to an improvement in air quality.[12] These findings are similar to the results of the present study. Previous studies have reported a mean annual rate of change in FEV1 of between -30 and -22 ml/year in healthy, non-smoking males and females aged > 65 years.[13] Patients with COPD had a mean annual change in FEV1 of 30–80 ml/year.[14] However, exposure to air pollution and tobacco smoke, both of which can result in tracheobronchial injury, can worsen annual rates of change in lung function markedly.

A high proportion of our subjects, particularly those with obstructive impairment, reported respiratory symptoms at baseline. These symptoms were more pronounced among the smokers in our sample. In Group D, however, the proportion of patients reporting asthma attacks, sputum production and cough had not increased, suggesting that these symptoms may be prevented by smoking cessation. The proportion of subjects in Group B reporting asthma attacks had increased by follow-up. Furthermore, the proportion of subjects reporting asthma attacks in Group C had increased to the same levels as that found in Group D at initial evaluation. Repeated bronchial inflammation caused by smoking and pollutant exposure can result in thickening of the bronchial wall and airway remodelling that cannot be reversed even by steroid treatment following the onset of hypersensitivity.[15] Furthermore, Siroux et al. have shown that continued tobacco use can significantly increase the incidence of asthma attacks.[16] Because of this association, smoking is listed as a potential trigger for asthma and COPD in asthma management guidelines.[17] Tobacco

For peer review only

use is also likely to have been the cause of the higher prevalence of severe cough and sputum production found in in Group A and Group C.

A number of studies have shown that smoking can cause respiratory tract inflammation and exacerbate symptoms such as cough and sputum production.[18]This was reflected in our finding that the severity of dyspnoea among subjects in Group A and Group B had increased markedly at follow-up. While a number of causes of dyspnoea have been identified, including age, poor physical fitness, muscle atrophy and social and environmental factors, we could not identify the primary determinants of dyspnoea in the present study.

[19] Finally, progression of respiratory disease and a decline in lung function can further aggravate these symptoms.[20]

In Asia, increased mortality and morbidity resulting from lung cancer, COPD, asthma and heart disorders caused by rising atmospheric PM 2.5 concentrations is a growing public health concern. According to the Organization for Economic Cooperation and Development, air pollution is expected to become the leading cause of death worldwide by 2050, and is likely to be responsible for between three and six million deaths annually.[21] Furthermore, Pope et al. have reported that worldwide pollution-related deaths could rise by 6% for each additional 10 µg/m3 increase in atmospheric PM 2.5 concentrations.[22]

The present study emphasizes the need to prevent or reduce air pollution and to improve public education of the negative health effects of smoking, to reduce pulmonary morbidity on the population level. This is especially the case among designated victims, for whom additional education programs, behavioural interventions and support for smoking cessation would be particularly beneficial. One such behavioural intervention that has been proven to be cost-effective in reducing smoking prevalence is the five-step Global Initiative for Obstructive Lung Disease (GOLD) framework (ask, advise, assess, assist, arrange).[23] This strategy, which aims at delaying

For peer review only - http://bmjopen.bmj.com/site/about/guidelines.xhtml

For peer review only

the progress of COPD through continuing support for smoking cessation, may be applicable for use in similarly affected communities.[24]

This study has several limitations. First, information on number of cigarettes smoked and individual-level exposure to environmental pollutants was not available. Second, it was not within the scope of the present study to explore the role of gender-specific differences in exposure to tobacco smoke and pollutants – much of which may have been occupational. Third, we were unable to clearly distinguish between the associations of mainstream and side-stream cigarette smoke when considering the associations of air pollution and health conditions. Finally, we were unable to account for possible confounding factors such as socio-economic class and lifestyle factors, which may have resulted in individual-level differences in exposure to environmental pollutants.

Further work is needed to inform future prevention and treatment programs to improve the quality-of-life of the remaining designated pollution victims in Japan. The results of the present study are of particular importance in the context of high rates of smoking and worsening air quality in a number of Asian countries as they undergo rapid economic change.

Acknowledgements

We thank the study participants, technical staff, administrative support team, and our co-workers for their help. We thank the Environmental Restoration and Conservation Agency and Mizushima-Kyodo Hospital for their support. In addition, English language in this manuscript has been edited by the Edanz Group Ltd (Fukuoka, Japan).

Funding

This work was supported by the Environmental Restoration and Conservation Agency and Mizushima-Kyodo Hospital.

For peer review only - http://bmjopen.bmj.com/site/about/guidelines.xhtml

Competing interests

The authors declare that they have no competing interests.

Contributions

HS was the principal investigator and contributed to the design of the study, administered the funding, supervised the team's work and made critical revisions to the manuscript for intellectual content. KK designed the study, collected data, analysed and interpreted data, and prepared the manuscript. SH took part in analysing and interpreting the data, and made critical revisions to the manuscript for intellectual content. All other authors took part in data collection and interpretation, and have read and approved the final manuscript.

Data sharing statement:

Additional unpublished data from the study are available from TT (tanakataka@nagasaki-u.ac.jp) and YY (dependonhero@yahoo.co.jp). They can be reached by email.

For peer review only

For peer review only - http://bmjopen.bmj.com/site/about/guidelines.xhtml

Table 1. Demographics and respiratory characteristics of comparison groups at initial evaluation for designation of air pollution victim status

		Group A (n=48)	Group B (n=169)	Group C (n=119)	Group D (n=394)	p	post-hoc test
Age		54.3 ± 8.3	49.6 ± 8.2	49.8 ± 7.8	51.3 ± 8.9	<.001	b.c.f
Gender (male/female)		35/13	66/103	81/38	90/304	<.001	a.b.c.d.e.f
Height	(cm)	161.7 ± 6.3	155.2 ± 7.6	161.4 ± 6.1	152.8 ± 7.5	<.001	a.b.d.e.f
Weight	(kg)	56.9 ± 6.4	55.4 ± 9.3	59.1 ± 8.9	55.2 ± 9.7	<.001	b.f
BMI		21.8 ± 1.9	22.8 ± 3.6	22.6 ± 2.8	23.4 ± 3.8	<.001	a.b.d.e.
FVC	(l)	3.37 ± 0.79	3.04 ± 0.79	3.49 ± 0.76	2.94 ± 0.68	<.001	a.b.e.f
%VC	(%)	105.5 ± 18.7	103.5 ± 18.5	108.3 ± 15.5	107.6 ± 17.3	<.05	d.f
FEV1	(l)	2.09 ± 0.64	1.72 ± 0.58	2.67 ± 0.58	2.24 ± 0.58	<.001	a.b.c.d.f
FEV1/FVC	(%)	64.9 ± 6.5	59.8 ± 10.3	79.1 ± 5.6	79.6 ± 7.1	<.001	a.c.d.e.f
Data of duration after certification	(yr)	25.9±4.1	26.9±4.4	24.8±4.2	25.5±4.6	<.001	d.f
Diagnosed pulmonary disease CB,BA,PE CB+BA CB+PE, BA+PE	no	32/10/1/5/ 0/ 0	52/98/3/11/3/2	98/14/0/7/ 0/ 0	249/99/0/46/0/0	<.001	a.c.d.f
	Unit Score						
Dyspnea	1	0 (0.0%)	1(0.6%)	0 (0.0%)	0 (0.0%)		
	2	0 (0.0%)	4 (2.4%)	0 (0.0%)	4 (1.1%)		
	3	9 (18.8%)	31 (18.3%)	7 (5.9%)	55 (15.8%)	<.001	b.c.d.f
	4	30 (62.5%)	111 (65.7%)	75 (63.0%)	246 (70.5%)		
	5	9 (18.8%)	22 (13.0%)	37 (31.1%)	89 (25.5%)		
Asthma attack	1	0 (0.0%)	1 (0.6%)	0 (0.0%)	1 (0.3%)		
	2	0 (0.0%)	8 (4.7%)	1 (0.8%)	13 (3.3%)		
	3	7 (14.6%)	51 (30.2%)	11 (9.2%)	66 (16.8%)	<.001	a.b.d.f
	4	14 (29.2%)	76 (45.0%)	30 (25.2%)	139 (35.3%)		
	5	27 (56.3%)	33 (19.5%)	77 (64.7%)	175 (44.4%)		
Cough and phlegm	1	0 (0.0%)	2 (1.2%)	0 (0.0%)	0 (0.0%)		
	2	0 (0.0%)	5 (3.0%)	1 (0.8%)	9 (2.3%)		
	3	24 (50.0%)	50 (29.6%)	58 (48.7%)	143 (36.3%)	<.05	a.b.d.f
	4	23 (47.9%)	90 (53.3%)	58 (48.7%)	225 (57.1%)		
	5	1 (2.1%)	22 (13.0%)	1 (0.8%)	15 (3.8%)		

For peer review only

For peer review only - http://bmjopen.bmj.com/site/about/guidelines.xhtml

Figures are presented as mean ±SD

FEV1: Forced Expiratory Volume in 1 second, FVC: Forced Vital Capacity, VC%: % Vital Capacity

FEV1/FVC (%) Forced Expiratory Volume in 1 second.

CB: Chronic Bronchitis BA: Bronchial Asthma PE: Pulmonary Emphysema

CB+BA CB+PE BA+PE

Differences in sample characteristics analysed using the Kruskal-Wallis test with Cochran-Armitage tests for significance

post-hoc tests: Mann-Whitney U test, chi-square test for significance

a: Group A vs Group B

b:Group C vs Group D

c: Group A vs Group C

d: Group B vs Group D

e: Group A vs Group D

f:Group B vs Group C

a, b: Current smokers / ex-smokers (A and C Groups) vs never smokers (B and D Groups)

c, d: Diagnosis of obstructive ventilatory defect (A and B Groups) vs no obstructive ventilatory defect (C and D Groups)

For peer review only

Table 2. Change in demographic characteristics and respiratory function of designated air pollution victims by comparison group between initial evaluation and follow-up

		Initial evaluation	Follow-up	p	Initial evaluation	Follow-up	p	Initial evaluation	Follow-up	p	Initial evaluation	Follow-up	p
		**Group** A			**Group B**			**Group** C			**Group D**		
Age		54.3 ± 8.3	78.6 ± 6.5	<.001	49.6 ± 8.2	77.3 ± 7.5	<.001	49.8 ± 7.8	74.2 ± 7.1	<.001	51.3 ± 8.9	76.5 ± 7.8	<.001
Height	(cm)	161.7 ± 6.3	159.3 ± 6.9	<.001	155.2 ± 7.6	150.8 ± 8.8	<.001	161.4 ± 6.1	159.2 ± 6.2	<.001	152.8 ± 7.5	148.9 ± 8.8	<.001
Weight	(kg)	56.9 ± 6.4	55.4 ± 9.2	<.001	55.4 ± 9.3	52.1 ± 10.9	<.001	59.1 ± 8.9	58.9 ± 9.6	<.001	55.2 ± 9.7	53.2 ± 11.3	<.001
BMI		21.8 ± 1.9	21.7 ± 2.9	<.001	22.8 ± 3.6	22.7 ± 3.9	<.001	22.6 ± 2.8	23.2 ± 3.1	<.05	23.4 ± 3.8	23.9 ± 4.2	0.13
PFT													
FVC	(l)	3.37 ± 0.79	2.54 ± 0.85	<.001	3.04 ± 0.79	2.21 ± 0.73	<.001	3.49 ± 0.76	2.78 ± 0.81	<.001	2.94 ± 0.68	2.28 ± 0.69	<.001
%VC	(%)	105.5 ± 18.7	93.1 ± 21.7	<.001	103.5 ± 18.5	90.3 ± 22.3	<.001	108.3 ± 15.5	100.2 ± 21.9	<.001	107 ± 17.3	100.4 ± 23.4	<.001
FEV1	(l)	2.09 ± 0.64	1.28 ± 0.53	<.001	1.72 ± 0.58	1.29 ± 0.51	<.001	2.67 ± 0.58	1.8 ± 0.68	<.001	2.24 ± 0.58	1.61 ± 0.52	<.001
FEV1 /FVC	(%)	64.9 ± 6.5	54.5 ± 2.3	<.001	59.8 ± 10.3	62.1 ± 13.4	<.001	79.1 ± 5.6	67.9 ± 10.2	<.001	79.6 ± 7.1	73.8 ± 13.8	<.001

PFT: Pulmonary Function Testing

Figures are presented as mean ±SD

Initial evaluation versus follow-up: Mann-Whitney U test

For peer review only

Table 3. Change in respiratory symptoms of designated air pollution victims by comparison group between initial evaluation and follow-up

	Unit Score	Initial evaluation		Follow-up		p	Initial evaluation		Follow-up		p	Initial evaluation		Follow-up		p	Initial evaluation		Follow-up		p
Dyspnoea		Group A					Group B					Group C					Group D				
	1	0	0.0%	1	2.1%		1	0.6%	2	1.2%		0	0.0%	1	0.8%		0	0.0%	0	0.0%	
	2	0	0.0%	4	8.3%		4	2.4%	15	8.9%		0	0.0%	2	1.7%		4	1.1%	13	3.7%	
	3	9	18.8%	24	50.0%	<.001	31	18.3%	74	43.8%	<.001	7	5.9%	20	16.8%	<.001	55	15.8%	67	19.2%	<.001
	4	30	62.5%	15	31.3%		111	65.7%	75	44.4%		75	63.0%	77	64.7%		246	70.5%	279	79.9%	
	5	9	18.8%	4	8.3%		22	13.0%	3	1.8%		37	31.1%	19	16.0%		89	25.5%	35	10.0%	
Asthma attack																					
	1	0	0.0%	0	0.0%		1	0.6%	0	0.0%		0	0.0%	0	0.0%		1	0.3%	0	0.0%	
	2	0	0.0%	3	6.3%		8	4.7%	19	11.2%		1	0.8%	4	3.4%		13	3.3%	10	2.5%	
	3	7	14.6%	16	33.3%	<.001	51	30.2%	72	42.6%	<.001	11	9.2%	17	14.3%	<.001	66	16.8%	71	18.0%	0.78
	4	14	29.2%	15	31.3%		76	45.0%	51	30.2%		30	25.2%	35	29.4%		139	35.3%	139	35.3%	
	5	27	56.3%	14	29.2%		33	19.5%	27	16.0%		77	64.7%	63	52.9%		175	44.4%	174	44.2%	
Cough and phlegm																					
	1	0	0.0%	1	2.1%		2	1.2%	1	0.6%		0	0.0%	0	0.0%		0	0.0%	0	0.0%	
	2	0	0.0%	3	6.3%		5	3.0%	8	4.7%		1	0.8%	2	1.7%		9	2.3%	9	2.3%	
	3	24	50.0%	34	70.8%	<.001	50	29.6%	99	58.6%	<.001	58	48.7%	66	55.5%	<.001	143	36.3%	155	39.3%	0.24
	4	23	47.9%	9	18.8%		90	53.3%	55	32.5%		58	48.7%	49	41.2%		227	57.1%	221	56.1%	
	5	1	2.1%	1	2.1%		22	13.0%	6	3.6%		2	0.8%	2	1.7%		15	3.8%	9	2.3%	

Respiratory symptoms

Numerical values are shown (%).

Initial evaluation versus follow-up: Wilcoxon signed-rank test

For peer review only

For peer review only - http://bmjopen.bmj.com/site/about/guidelines.xhtml

Table 4. Mean annual change in yearly spirometry results from initial evaluation to follow-up by comparison group

		Group A (n=48)	Group B (n=169)	Group C (n=119)	Group D (n=394)	p	post-hoc test
FEV1 decline/year	(ml)	-36.7±19.1	-26.5±18.5	-37.2±16.4	-25.5±17.2	<.001	a.b.d.e.f
FEV1/FVC decline/year	(%)	-0.04±0.05	0.09±0.06	-0.04±0.03	-0.02±0.04	<.001	a.b.d.e.f
FVC decline/year	(ml)	-31.7±24.5	-30.1±20.7	-29.9±22.8	-25.3±17.1	<.05	d
%VC decline/year	(%)	-0.05±0.08	-0.05±0.08	-0.03±0.06	-0.03±0.07	<.001	c.d.e.f

Figures are presented as mean ±SD

p-values were calculated using the Kruskal-Wallis test post-hoc test: Mann-Whitney U test

a: Group A vs Group B

b:Group C vs Group D

c: Group A vs Group C

d: Group B vs Group D

e: Group A vs Group D

f:Group B vs Group C

a, b: Current smokers / ex-smokers (A and C Groups) vs never smokers (B and D Groups)

c, d: Diagnosis of obstructive ventilatory defect (A and B Groups) vs no obstructive ventilatory defect (C and D Groups)

For peer review only

Table 5. Change in severity of smoking-related respiratory symptoms by comparison group at follow-up

Score	Group A (n=48)		Group B (n=169)		Group C (n=119)		Group D (n=394)		p	post-hoc test
Dyspnea										
1	1	2.1%	2	1.2%	1	0.8%	0	0.0%		
2	4	8.3%	15	8.9%	2	1.7%	13	3.7%		
3	24	50.0%	74	43.8%	20	16.8%	67	19.2%	<.001	c.d.e.f
4	15	31.3%	75	44.4%	77	64.7%	279	79.9%		
5	4	8.3%	3	1.8%	19	16.0%	35	10.0%		
Asthma attack										
1	0	0.0%	0	0.0%	0	0.0%	0	0.0%		
2	3	6.3%	19	11.2%	4	3.4%	10	2.5%		
3	16	33.3%	72	42.6%	17	14.3%	71	18.0%	<.001	a.c.d.e.f
4	15	31.3%	51	30.2%	35	29.4%	139	35.3%		
5	14	29.2%	27	16.0%	63	52.9%	174	44.2%		
Cough and phlegm										
1	1	2.1%	1	0.6%	0	0.0%	0	0.0%		
2	3	6.3%	8	4.7%	2	1.7%	9	2.3%		
3	34	70.8%	99	58.6%	66	55.5%	155	39.3%	<.001	a.b.c.d.e.
4	9	18.8%	55	32.5%	49	41.2%	221	56.1%		
5	1	2.1%	6	3.6%	2	1.7%	9	2.3%		

Numerical values are shown (%)

p-values were calculated using the Kruskal-Wallis test post-hoc test: Mann–Whitney U test

a: Group A vs Group B

b:Group C vs Group D

c: Group A vs Group C

d: Group B vs Group D

e: Group A vs Group D

f:Group B vs Group C

a, b: Current smokers / ex-smokers (A and C Groups) vs never smokers (B and D Groups)

c, d: Diagnosis of obstructive ventilatory defect (A and B Groups) vs no obstructive ventilatory defect (C and D Groups)

For peer review only - http://bmjopen.bmj.com/site/about/guidelines.xhtml

Figure legends

Figure 1 Flowchart showing the selection procedure for study subjects.

The study included officially designated victims of pollution-related illness in Kurashiki aged 65 years or older in 2009, and for whom full data were available for statistical analysis.

Ceased to experience symptoms : Officially designated victims who did renew the designation because of disappearance of clinical symptoms.

Figure 2 Sulfur dioxide and nitrogen dioxide concentrations from 1965 to 2009 relative to standard values.

REFERENCES

1. Yang G, Wang Y, Zeng Y, et al. Rapid health transition in China, 1990-2010: findings from the Global Burden of Disease Study 2010. Lancet 2013;**381**(9882):1987-2015 doi: 10.1016/S0140-6736(13)61097-1
S0140-6736(13)61097-1 [pii][published Online First: Epub Date]|.
2. Liu Y, Lee K, Perez-Padilla R, et al. Outdoor and indoor air pollution and COPD-related diseases in high- and low-income countries. Int J Tuberc Lung Dis 2008;**12**(2):115-27
3. Yang JY, Xin JY, Ji DS, et al. [Variation analysis of background atmospheric pollutants in North China during the summer of 2008 to 2011]. Huan Jing Ke Xue 2012;**33**(11):3693-704
4. Tanaka T, Asai M, Yanagita Y, et al. Longitudinal study of respiratory function and symptoms in a non-smoking group of long-term officially-acknowledged victims of pollution-related illness. BMC Public Health 2013;**13**:766 doi: 10.1186/1471-2458-13-766
1471-2458-13-766 [pii][published Online First: Epub Date]|.
5. Usmanova G, Mokdad AH. Results of the Global Youth Tobacco Survey and implementation of the WHO Framework Convention on Tobacco Control in the WHO Eastern Mediterranean Region (EMR) countries. J Epidemiol Glob Health 2013;**3**(4):217-34 doi: 10.1016/j.jegh.2013.07.001
S2210-6006(13)00084-1 [pii][published Online First: Epub Date]|.
6. Yang G, Fan L, Tan J, et al. Smoking in China: findings of the 1996 National Prevalence Survey. JAMA 1999;**282**(13):1247-53 doi: joc80784 [pii][published Online First: Epub Date]|.
7. M C. WHO report on the global tobacco epidemic 2008. Geneva:World Health Organization 2008
8. Yamagata T, Hirano T, Sugiura H, et al. Comparison of bronchodilatory properties of transdermal and inhaled long-acting beta 2-agonists. Pulm Pharmacol Ther 2008;**21**(1):160-5 doi: S1094-5539(07)00047-8 [pii]
10.1016/j.pupt.2007.05.004[published Online First: Epub Date]|.

For peer review only - http://bmjopen.bmj.com/site/about/guidelines.xhtml

9. Baldwin ED CA, Richards DW Jr. Pulmonary insufficiency; physiological classification, clinical methods of analysis, standard values in normal subjects. Medicine (Baltimore) 1948; **27(3)**:243-78
10. Curjuric I, Imboden M, Nadif R, et al. Different genes interact with particulate matter and tobacco smoke exposure in affecting lung function decline in the general population. PLoS One 2012;**7**(7):e40175 doi: 10.1371/journal.pone.0040175
PONE-D-11-21280 [pii][published Online First: Epub Date]|.
11. Ceylan E, Kocyigit A, Gencer M, et al. Jul 2006 patent 0954-6111 (Print)
0954-6111 (Linking).
12. Downs SH, Schindler C, Liu LJ, et al. Reduced exposure to PM10 and attenuated age-related decline in lung function. N Engl J Med 2007;**357**(23):2338-47 doi: 357/23/2338 [pii]
10.1056/NEJMoa073625[published Online First: Epub Date]|.
13. Lee PN, Fry JS. Systematic review of the evidence relating FEV1 decline to giving up smoking. BMC Med 2010;**8**:84 doi: 10.1186/1741-7015-8-84
1741-7015-8-84 [pii][published Online First: Epub Date]|.
14. Fletcher C, Peto R. The natural history of chronic airflow obstruction. British medical journal 1977;**1**(6077):1645-8
15. Downs SH, Schindler C, Liu LJ, et al. Reduced exposure to PM10 and attenuated age-related decline in lung function. N Engl J Med 2007;**357**(23):2338-47 doi: 10.1056/NEJMoa073625[published Online First: Epub Date]|.
16. Siroux V, Pin I, Oryszczyn MP, et al. Relationships of active smoking to asthma and asthma severity in the EGEA study. Epidemiological study on the Genetics and Environment of Asthma. Eur Respir J 2000;**15**(3):470-7
17. Gruffydd-Jones K. GOLD guidelines 2011: what are the implications for primary care? Primary care respiratory journal : journal of the General Practice Airways Group 2012;**21**(4):437-41 doi: 10.4104/pcrj.2012.00058[published Online First: Epub Date]|.
18. Pelkonen M, Notkola IL, Nissinen A, et al. Thirty-year cumulative incidence of chronic bronchitis and COPD in relation to 30-year pulmonary function and 40-year mortality: a follow-up in middle-aged rural men. Chest 2006;**130**(4):1129-37 doi: 130/4/1129 [pii]
10.1378/chest.130.4.1129[published Online First: Epub Date]|.
19. Berglund DJ, Abbey DE, Lebowitz MD, et al. Respiratory symptoms and pulmonary function in an elderly nonsmoking population. Chest 1999;**115**(1):49-59
20. Oga T TM, Hajiro T, Ikeda A, Nishimura K. Analysis of longitudinal changes in dyspnea of patients with chronic obstructive pulmonary disease: an observational study. Respir Res 2012;**13**:85
21. Dong GH, Zhang P, Sun B, et al. Long-term exposure to ambient air pollution and respiratory disease mortality in Shenyang, China: a 12-year population-based retrospective cohort study. Respiration; international review of thoracic diseases 2012;**84**(5):360-8 doi: 10.1159/000332930[published Online First: Epub Date]|.
22. C. Arden Pope I caRTB, Michelle C. Turner, Aaron Cohen, Daniel Krewski, Michael Jerrett, Susan M. Gapstur, and Michael J. Thun. Lung Cancer and Cardiovascular Disease Mortality Associated with Ambient Air Pollution and Cigarette Smoke: Shape of the Exposure–Response Relationships. Environ Health Perspect 2011
119(11): 1616–21
23. Kaufman G. Chronic obstructive pulmonary disease: diagnosis and management. Nurs Stand 2013;**27**(21):53-7, 60-2
24. website.http GIfCOLDG, //www.goldcopd.org/. Global strategy for the diagnosisGlobal strategy for the diagnosis. management and prevention of COPD, 2011.

For peer review only - http://bmjopen.bmj.com/site/about/guidelines.xhtml

Title

Tobacco use among designated air pollution victims and its association with lung function and respiratory symptoms: a retrospective cross-sectional study

Authors

Kenji Kotaki, Hideaki Senjyu*, Takako Tanaka, Yudai Yano, Naomi Miyamoto, Tsuyoshi Nishinakagawa, Yorihide Yanagita, Masaharu Asai, Ryo Kozu, Mitsuru Tabusadani, Terumitsu Sawai, Sumihisa Honda

Author details

Department of Cardiopulmonary Rehabilitation Science, Nagasaki University Graduate School of Biomedical Sciences, Nagasaki, Japan

***Corresponding author**

Department of Cardiopulmonary Rehabilitation Science

Nagasaki University Graduate School of Biomedical Sciences

1-7-1 Sakamoto, Nagasaki 852-8520, Japan

Email: senjyu@nagasaki-u.ac.jp

Tel: +81-95-819-7963; Fax: +81-95-819-7963

Keywords: Air pollution, Tobacco, Respiratory function, Respiratory symptoms, Pulmonary function

Word count: 3730 words

For peer review only - http://bmjopen.bmj.com/site/about/guidelines.xhtml

ABSTRACT

Objectives: We sought to elucidate the long-term association of tobacco use and respiratory health in designated pollution victims with and without obstructive pulmonary defects.

Design: A retrospective cross-sectional study.

Setting: The register of pollution victims in Kurashiki, Japan.

Participants: 730 individuals over 65 years of age previously diagnosed with pollution-related respiratory disease. Patients were classified into four groups according to their smoking status and whether or not they had obstructive pulmonary disease. We then compared the prevalence of respiratory symptoms and lung function over time between groups.

Primary outcome measures: Spirometry was performed and a respiratory health questionnaire completed in the same season each year for up to 30 years.

Results: Rates of smoking and respiratory disease were high in our sample. While respiratory function in non-smoking patients did not completely recover, the annual rate of change in lung function was within the normal range ($p < 0.01$). However, smokers had worse lung function and were more likely to report more severe pulmonary symptoms ($p < 0.01$).

Conclusions: Patients' respiratory function did not fully recover despite improved air quality. Our results suggest that, in the context of exposure to air pollution, tobacco use causes additional loss of lung function and exacerbates respiratory symptoms.

Article summary

Strengths and limitations

- This first study to have analysed changes in respiratory function and respiratory symptoms in a group of designated pollution victims in Japan over up to 30 years.
- Tobacco use caused additional loss of lung function and exacerbated respiratory symptoms in those who have been exposed to air pollution.
- We did not have data on the quantity of cigarettes smoked, the duration of cigarette smoking, or individual-level exposure to environmental pollutants.

For peer review only - http://bmjopen.bmj.com/site/about/guidelines.xhtml

- We were unable to account for possible confounding factors such as socio-economic class and other lifestyle factors.

For peer review only

For peer review only - http://bmjopen.bmj.com/site/about/guidelines.xhtml

INTRODUCTION

In many Asian nations, particularly China, air pollution is increasingly being recognised as an important emerging environmental and public health issue. Previous work has shown that levels of ambient air pollution are associated with an elevated incidence of respiratory diseases and reported prevalence of respiratory symptoms,[1] particularly asthma, pulmonary emphysema and chronic bronchitis.[2] Atmospheric pollution caused by particulate matter less than 2.5 μm in diameter (PM2.5) is reported to be particularly acute in China and in surrounding countries as a result of wind dispersal. This is a growing public health issue in the region, especially given that PM 2.5 is capable of penetrating deep into lung tissue and precipitating a number of respiratory diseases.[3]

Japan was also severely affected during its period of accelerated economic growth in the wake of post-war reconstruction in the 1960s, with both air pollution and the prevalence of respiratory diseases rising markedly. In Kurashiki, a city located in Okayama Prefecture, reports of pulmonary toxicity owing to oxidant air pollutants rose by a factor of 1.73 among the city's inhabitants in the period leading up to 1970 as yearly average SO3 levels increased. The majority of excess pollution-related deaths in this period were caused by acute exacerbation of asthma.

Although patients do not commonly recover fully from pollution-related respiratory diseases, FEV1/ FVC ratios (calculated by dividing Forced Expiratory Volume exhaled at the end of the first second of forced expiration by Forced Vital Capacity) may return to within the normal range once air quality is improved.[4] As a result, Japan passed the Pollution-Related Health Damage Compensation Law in 1973 with the aim of identifying designated air pollution victims within affected areas for compensation. Patients with a diagnosis of bronchial asthma, chronic bronchitis or pulmonary emphysema who had been resident in any designated air pollution zone for a specified period of time were legally recognised as pollution victims. These designated victims were fully reimbursed the costs of all related medical treatment by the state. However, patients' lung function, as measured

For peer review only - http://bmjopen.bmj.com/site/about/guidelines.xhtml

using FEV1/FVC ratios, was not among the diagnostic criteria used to determine eligibility for compensation, as no agreed clinical definition for Chronic Obstructive Pulmonary Disease (COPD) existed at that time.

Tobacco smoke has been shown to contain high levels of PM 2.5, and is likely to be associated with many health issues in common with air pollution. Tobacco use has been shown to reduce lung function, as measured using FEV1/ FVC ratios, and has been identified as a cause of COPD and other tobacco-related diseases. The World Health Organization (WHO) Framework Convention on Tobacco Control was therefore adopted in 2003.[5] However, tobacco use remains a significant public health issue. According to a WHO report, more than 1.1 billion people globally are regular smokers.[6] Furthermore, Asia accounts for a third of total world cigarette consumption, and there has been a local rise in air pollution. [7]

Both air pollution and tobacco smoke have been shown to reduce patients' FEV1/FVC ratios, to increase the risk of obstructive ventilatory defects, and to exacerbate existing respiratory symptoms. Tanaka et al. have reported that when air quality is improved, the annual change in patients' FEV1 can return to within the normal range.[4] However, the association of ongoing tobacco use with respiratory function and pulmonary symptoms in patients with already reduced FEV1/FVC ratios resulting from bronchial hypersensitivity due to air pollution has not yet been elucidated.

The purpose of the present study, carried out among a population of confirmed pollution victims living in a designated pollution-affected area, was to determine the associations between tobacco use and measures of respiratory function and prevalence of pulmonary symptoms up to 30 years after an improvement in air quality. In particular, we sought to compare these associations in patients with and without diagnosed obstructive ventilatory defects resulting from exposure to air pollution.

For peer review only - http://bmjopen.bmj.com/site/about/guidelines.xhtml

For peer review only

METHODS

Study design and setting

The present study was embedded in a retrospective cross-sectional analysis of lung function and respiratory symptoms in designated victims of pollution-related illness in Kurashiki between their initial evaluation (from 1976 to 1988) and follow-up in 2009. Study subjects were drawn from the register of pollution victims in Kurashiki. All study participants met the following conditions, as determined by the Public Relief System of Kurashiki City, in accordance with the Pollution-Related Health Damage Special Measures Law (1969) and the Pollution-Related Health Damage Compensation Law (1973): (1) they had resided or were employed within a designated air pollution zone, and (2) they were diagnosed with chronic bronchitis, asthma, or emphysema by a medical practitioner.

In accordance with the Public Nuisance Countermeasures Law (1967), registered victims were entitled to various forms of compensation including a monthly consultation with a doctor, yearly assessments of respiratory symptoms consisting of a detailed questionnaire and spirometry tests. These patients received treatment with inhaled corticosteroids and long-acting β2 agonists and expectorants, which may have caused improvements in lung function.[8] However, this study did not collect detailed data regarding treatment regimen, and we were therefore unable to evaluate the association between tobacco use and treatment.

Of 419,203 total residents (204,958 males, 214,245 females), 3,838 (0.9%) were officially classified as designated pollution victims in the period up to 1988. The records of these 3,838 residents were reviewed in 2009 with the permission of the Kurashiki City Public Office (Figure 1). Of the 1,392 remaining ‘officially designated victims of pollution-related illness’, we screened 774 individuals aged ≥ 65 years for inclusion in the study, to focus the study on elderly people. The elderly people

For peer review only - http://bmjopen.bmj.com/site/about/guidelines.xhtml

have a higher risk factor for the onset of COPD and show remarkably more respiratory symptoms of COPD, as we restricted to the patients over 65 years in this study. After excluding 44 records that did not have complete spirometry data, the resulting sample size was 730.

We classified patients into two groups according to whether they had an obstructive impairment using the FEV1/FVC ratios recorded at their initial evaluation. Participants in each group were then further sub-divided according to whether they had ever smoked or not(current smoker or ex-smoker or ever smoked) – determined using self-reporting at follow-up, but we could not identify smoking period. This resulted in four comparison groups, labelled as Group A (n=48, current smokers and ex-smokers diagnosed with an obstructive impairment reporting), Group B (n=169, non-smokers diagnosed with an obstructive impairment), Group C (n=119, smokers and ex-smokers without a diagnosed obstructive impairment) and Group D (n=394, non-smokers without an obstructive impairment). The majority of these patients were diagnosed with chronic bronchitis (n = 528, 68.2%), asthma (n = 242, 31.3%), or emphysema (n = 4, 0.5%), as described in case reports provided by authorized medical practitioners.

The study protocol was approved by the Ethical Committee of the Nagasaki University Graduate School of Biomedical Sciences.

Clinical measurements

Patients' gender, height, weight, Body Mass Index (BMI), FVC, Vital Capacity (%VC), FEV1, FEV1/ FVC ratios, diagnosed respiratory conditions and respiratory symptoms were all recorded at baseline and at follow-up.

Spirometry measurements

Spirometry was performed by trained staff at the Mizushima-Kyodo Hospital using an electronic

For peer review only - http://bmjopen.bmj.com/site/about/guidelines.xhtml

spirometry device (FUDAC 70, Fukuda Sangyo Inc., Chiba, Japan). Tests were performed on patients in the sitting position, and were repeated until at least three reproducible forced expiratory curves had been obtained. Both FEV1 and FVC were measured and used to calculate FEV1/FVC ratios in addition to %VC using the method proposed by Baldwin et al. [9]

Annual change of spirometric data

The mean annual change in spirometry data was calculated by subtracting the follow-up results from the initial evaluation results and dividing by the number of years between initial evaluation and follow-up.

Respiratory symptoms

Respiratory symptoms (including dyspnoea, asthma attacks, cough, and sputum production) were assessed by physicians in the same season each year using a respiratory health questionnaire designed by the government of Japan. Each symptom was graded using a standardised five-point scale.

Dyspnoea

1. Too breathless to leave the house, or breathless when dressing or undressing.
2. Breathless after walking about 50 m or after a few minutes on level ground.
3. Breathless when walking on level ground and keeping up with people of the same age, but not breathless when walking at own pace.
4. Breathless when walking up a slight hill or the stairs.
5. Breathless only during strenuous exercise.

Wheeze

1. Severe episode ≥ 10 days each month during the last year.

For peer review only - http://bmjopen.bmj.com/site/about/guidelines.xhtml

2. Severe episode ≥ 5 days each month during the last year, or mild episode ≥ 10 days each month during the last year.

3. Severe episode ≥ 1 day each month during the last year, or mild episode ≥ 5 days each month during the last year.

4. Mild episode ≥ 1 day each month during the last year.

5. No episodes of wheezing.

Cough and sputum

1. Daily cough and sputum, with a large amount of sputum or difficulty clearing sputum.

2. Daily cough and sputum, with a moderate amount of sputum or difficulty clearing sputum.

3. Daily cough and sputum, but not troublesome during daily life.

4. Daily cough and sputum for ≤ 3 months each year.

5. No cough or sputum.

Air pollution monitoring

Data on mean daily concentrations of air pollutants were obtained at 21 points in Kurashiki. Monitoring of SO2 concentrations started in 1965, while monitoring of nitrogen dioxide (NO2) concentrations commenced in 1971.

Statistical analysis

Data from patients in each of the four comparison groups were compared using the Kruskal-Wallis test and the Mann-Whitney U test as a post-hoc test. Differences in gender distributions and the prevalence of respiratory diagnoses in each group were analysed using the Cochran-Armitage test, using the chi-square test to test for statistical significance. Changes in lung function between initial evaluation and follow-up were analysed using the Wilcoxon signed-rank test and the Mann-Whitney U test as a post-hoc test. A normality test was performed on all continuous variables using the

For peer review only - http://bmjopen.bmj.com/site/about/guidelines.xhtml

Kolmogorov-Smirnov test before analysis. All analyses were performed using PASW Statistics, version 18. Finally, p-values of <0.05 were considered statistically significant.

RESULTS

Air pollutants

Figure 2 shows the annual mean daily levels of SO_2 and NO_2 recorded from 1965 to 2009 in Kurashiki. The Air Pollution Control Law was enacted in 1968. SO_2 levels were above the acceptable level every year from 1968 to 1974 and then decreased to below 40 parts per billion (ppb), which is the acceptable level defined by the Air Pollution Control Law. In 1973, the acceptable NO_2 level was changed by the Air Pollution Control Law from 20 ppb to 40 ppb. The NO_2 level exceeded the revised acceptable level only in 1973.

Sample characteristics

Table 1 shows the characteristics of our patient sample at initial evaluation. We found significant differences in the age distributions between groups ($p<0.001$). Males outnumbered females in the Group A and Group C ($p<0.001$). Patients in the Group B and Group D had significantly lower heights and weights ($p<0.001$).

We found differences in FVC values between groups, with patients in the Group B and Group D presenting significantly lower values than those in the Group A and Group C ($p<0.001$). FEV1 values, which also differed significantly between comparison groups ($p<0.001$), were lowest in the Group B, followed by in the Group A. %VC and FEV1/FVC ratios were lowest the Group A and Group B. Finally, the prevalence of bronchial asthma at initial evaluation was much higher in the Group B compared with all other groups ($p<0.001$).

For peer review only - http://bmjopen.bmj.com/site/about/guidelines.xhtml

For peer review only

Change in respiratory function and symptoms between initial evaluation and follow-up

Table 2 and 3 show the results of our evaluation of the change in severity of respiratory symptoms and respiratory function between initial evaluation and follow-up. Respiratory function had deteriorated in almost all subjects by follow-up. In particular, FEV/FVC ratios in Group C, which averaged 79.1±5.6% at baseline, had fallen to 67.9±10.2% at follow-up because of the development of obstructive ventilatory defects in a number of subjects. Conversely, average FEV/FVC ratios increased from 59.8±10.3% to 62.1±13.4% in Group B ($p<0.001$). The median level of respiratory symptoms worsened from level II to level III in all groups. However, Group D saw a decrease in the proportion of patients reporting asthma attacks ($p=0.78$), cough, and sputum production ($p=0.24$) over the period studied.

Mean annual change in respiratory function

Table 4 shows the mean annual change in respiratory function in each comparison group.

Mean FEV1 decreased to a similar extent in both Group A and Group C. While there was a slight fall in FEV1 in Group D, there was a marginal improvement in FEV1 in Group B ($p<0.001$). At the same time, while FEV1/FVC ratios fell ($p<0.001$) in both Group A and Group C, an improvement was seen in Group B. Finally, %VC fell in all groups ($p<0.001$), although the decline was greater in Group A and Group B than in Group C and Group D, as shown in Table 4.

Comparison of respiratory symptoms at final evaluation

Table 5 shows the severity of respiratory symptoms in our four comparison groups at follow-up.

For peer review only - http://bmjopen.bmj.com/site/about/guidelines.xhtml

For peer review only

In Group A and Group B, whose subjects were diagnosed with obstructive ventilatory defects at baseline, the great majority reported severe dyspnoea, asthma, cough and sputum production. Severe dyspnoea was also highly prevalent in Group A and Group B. While severity of asthma was high in both Group B and Group D at initial evaluation, the proportion of patients reporting severe asthma had only increased significantly in Group A and Group C at follow-up ($p<0.001$). Severity of asthma was similar in Group C and Group D at follow-up. Prevalence of severe cough and sputum production increased significantly in Group A and Group C ($p<0.001$), with 80% of subjects in Group A reporting scores that changed from I to III due to a worsening of symptoms.

DISCUSSION

This retrospective cross-sectional study analysed changes in respiratory function and respiratory symptoms between two points in time (initial evaluation and follow-up) among a group of designated pollution victims in Japan. Our results show for the first time that improvements in respiratory function and reductions in respiratory symptoms are prevented by continuing tobacco use.

Among those patients diagnosed with an obstructive impairment due to air pollution exposure (Groups A and B), we found that FEV1/FVC ratios had deteriorated by follow-up with no reduction in morbidity. However, our results showed an improvement in FEV1/FVC ratios in Group B, which could be attributed to the high proportion of subjects in this group diagnosed with bronchial asthma at initial evaluation when compared with the other groups.

We observed that respiratory function had deteriorated among patients in Group A despite the improvement in local air quality, resulting in depressed FEV1/FVC ratios. Furthermore, while FEV1/FVC ratios in Group C were relatively low at the time of initial diagnosis, continuing tobacco use had resulted in some cases in obstructive impairment, causing FEV1/FVC ratios at follow-up to

For peer review only - http://bmjopen.bmj.com/site/about/guidelines.xhtml

decrease to 67.9±10.2%.

While air pollution can cause airway obstruction resulting in asthma, reactive airways dysfunction syndrome and reduced lung function, tobacco use, which can result in airway hyper-responsiveness, has been shown to exert a stronger influence on respiratory health in a number of studies.[10 11] These associations were evident in Group A and Group C, which showed similar annual rates of change in respiratory function over time. Conversely, Downs et al. have shown that, in non-smokers, annual rates of change in FEV1 can return to within the normal range in response to an improvement in air quality.[12] These findings are similar to the results of the present study. Previous studies have reported a mean annual rate of change in FEV1 of between -30 and -22 ml/year in healthy, non-smoking males and females aged > 65 years.[13] Patients with COPD had a mean annual change in FEV1 of 30–80 ml/year.[14] However, exposure to air pollution and tobacco smoke, both of which can result in tracheobronchial injury, can worsen annual rates of change in lung function markedly.

A high proportion of our subjects, particularly those with obstructive impairment, reported respiratory symptoms at baseline. These symptoms were more pronounced among the smokers in our sample. In Group D, however, the proportion of patients reporting asthma attacks, sputum production and cough had not increased, suggesting that these symptoms may be prevented by smoking cessation. The proportion of subjects in Group B reporting asthma attacks had increased by follow-up. Furthermore, the proportion of subjects reporting asthma attacks in Group C had increased to the same levels as that found in Group D at initial evaluation. Repeated bronchial inflammation caused by smoking and pollutant exposure can result in thickening of the bronchial wall and airway remodelling that cannot be reversed even by steroid treatment following the onset of hypersensitivity.[15] Furthermore, Siroux et al. have shown that continued tobacco use can significantly increase the incidence of asthma attacks.[16] Because of this association, smoking is listed as a potential trigger for asthma and COPD in asthma management guidelines.[17] Tobacco

For peer review only

use is also likely to have been the cause of the higher prevalence of severe cough and sputum production found in in Group A and Group C.

A number of studies have shown that smoking can cause respiratory tract inflammation and exacerbate symptoms such as cough and sputum production.[18]This was reflected in our finding that the severity of dyspnoea among subjects in Group A and Group B had increased markedly at follow-up. While a number of causes of dyspnoea have been identified, including age, poor physical fitness, muscle atrophy and social and environmental factors, we could not identify the primary determinants of dyspnoea in the present study.

[19] Finally, progression of respiratory disease and a decline in lung function can further aggravate these symptoms.[20]

In Asia, increased mortality and morbidity resulting from lung cancer, COPD, asthma and heart disorders caused by rising atmospheric PM 2.5 concentrations is a growing public health concern. According to the Organization for Economic Cooperation and Development, air pollution is expected to become the leading cause of death worldwide by 2050, and is likely to be responsible for between three and six million deaths annually.[21] Furthermore, Pope et al. have reported that worldwide pollution-related deaths could rise by 6% for each additional 10 μg/m3 increase in atmospheric PM 2.5 concentrations.[22]

The present study emphasizes the need to prevent or reduce air pollution and to improve public education of the negative health effects of smoking, to reduce pulmonary morbidity on the population level. This is especially the case among designated victims, for whom additional education programs, behavioural interventions and support for smoking cessation would be particularly beneficial. One such behavioural intervention that has been proven to be cost-effective in reducing smoking prevalence is the five-step Global Initiative for Obstructive Lung Disease (GOLD) framework (ask, advise, assess, assist, arrange).[23] This strategy, which aims at delaying

For peer review only

For peer review only - http://bmjopen.bmj.com/site/about/guidelines.xhtml

the progress of COPD through continuing support for smoking cessation, may be applicable for use in similarly affected communities.[24]

This study has several limitations. First, information on number of cigarettes smoked and individual-level exposure to environmental pollutants was not available. Second, it was not within the scope of the present study to explore the role of gender-specific differences in exposure to tobacco smoke and pollutants – much of which may have been occupational. Third, we were unable to clearly distinguish between the associations of mainstream and side-stream cigarette smoke when considering the associations of air pollution and health conditions. Finally, we were unable to account for possible confounding factors such as socio-economic class and lifestyle factors, which may have resulted in individual-level differences in exposure to environmental pollutants.

Further work is needed to inform future prevention and treatment programs to improve the quality-of-life of the remaining designated pollution victims in Japan. The results of the present study are of particular importance in the context of high rates of smoking and worsening air quality in a number of Asian countries as they undergo rapid economic change.

Acknowledgements

We thank the study participants, technical staff, administrative support team, and our co-workers for their help. We thank the Environmental Restoration and Conservation Agency and Mizushima-Kyodo Hospital for their support.

Funding

This work was supported by the Environmental Restoration and Conservation Agency and Mizushima-Kyodo Hospital.

Competing interests

For peer review only - http://bmjopen.bmj.com/site/about/guidelines.xhtml

The authors declare that they have no competing interests.

Contributions

HS was the principal investigator and contributed to the design of the study, administered the funding, supervised the team's work and made critical revisions to the manuscript for intellectual content. KK designed the study, collected data, analysed and interpreted data, and prepared the manuscript. SH took part in analysing and interpreting the data, and made critical revisions to the manuscript for intellectual content. All other authors took part in data collection and interpretation, and have read and approved the final manuscript.

Data sharing statement:

Additional unpublished data from the study are available from TT (tanakataka@nagasaki-u.ac.jp) and YY (dependonhero@yahoo.co.jp). They can be reached by email.

For peer review only - http://bmjopen.bmj.com/site/about/guidelines.xhtml

Table 1. Demographics and respiratory characteristics of comparison groups at initial evaluation for designation of air pollution victim status

		Group A (n=48)	Group B (n=169)	Group C (n=119)	Group D (n=394)	p	post-hoc test
Age		54.3 ± 8.3	49.6 ± 8.2	49.8 ± 7.8	51.3 ± 8.9	<.001	b.c.f
Gender (male/female)		35/13	66/103	81/38	90/304	<.001	a.b.c.d.e.f
Height	(cm)	161.7 ± 6.3	155.2 ± 7.6	161.4 ± 6.1	152.8 ± 7.5	<.001	a.b.d.e.f
Weight	(kg)	56.9 ± 6.4	55.4 ± 9.3	59.1 ± 8.9	55.2 ± 9.7	<.001	b.f
BMI		21.8 ± 1.9	22.8 ± 3.6	22.6 ± 2.8	23.4 ± 3.8	<.001	a.b.d.e.
FVC	(l)	3.37 ± 0.79	3.04 ± 0.79	3.49 ± 0.76	2.94 ± 0.68	<.001	a.b.e.f
%VC	(%)	105.5 ± 18.7	103.5 ± 18.5	108.3 ± 15.5	107.6 ± 17.3	<.05	d.f
FEV1	(l)	2.09 ± 0.64	1.72 ± 0.58	2.67 ± 0.58	2.24 ± 0.58	<.001	a.b.c.d.f
FEV1/FVC	(%)	64.9 ± 6.5	59.8 ± 10.3	79.1 ± 5.6	79.6 ± 7.1	<.001	a.c.d.e.f
Data of duration after certification	(yr)	25.9±4.1	26.9±4.4	24.8±4.2	25.5±4.6	<.001	d.f
Diagnosed pulmonary disease CB,BA,PE CB+BA CB+PE, BA+PE	no	32/10/1/5/ 0/ 0	52/98/3/11/3/2	98/14/0/7/ 0/ 0	249/99/0/46/0/0	<.001	a.c.d.f
	Unit Score						
Dyspnea	1	0 (0.0%)	1(0.6%)	0 (0.0%)	0 (0.0%)	<.001	b.c.d.f
	2	0 (0.0%)	4 (2.4%)	0 (0.0%)	4 (1.1%)		
	3	9 (18.8%)	31 (18.3%)	7 (5.9%)	55 (15.8%)		
	4	30 (62.5%)	111 (65.7%)	75 (63.0%)	246 (70.5%)		
	5	9 (18.8%)	22 (13.0%)	37 (31.1%)	89 (25.5%)		
Asthma attack	1	0 (0.0%)	1 (0.6%)	0 (0.0%)	1 (0.3%)	<.001	a.b.d.f
	2	0 (0.0%)	8 (4.7%)	1 (0.8%)	13 (3.3%)		
	3	7 (14.6%)	51 (30.2%)	11 (9.2%)	66 (16.8%)		
	4	14 (29.2%)	76 (45.0%)	30 (25.2%)	139 (35.3%)		
	5	27 (56.3%)	33 (19.5%)	77 (64.7%)	175 (44.4%)		
Cough and phlegm	1	0 (0.0%)	2 (1.2%)	0 (0.0%)	0 (0.0%)	<.05	a.b.d.f
	2	0 (0.0%)	5 (3.0%)	1 (0.8%)	9 (2.3%)		
	3	24 (50.0%)	50 (29.6%)	58 (48.7%)	143 (36.3%)		
	4	23 (47.9%)	90 (53.3%)	58 (48.7%)	225 (57.1%)		
	5	1 (2.1%)	22 (13.0%)	1 (0.8%)	15 (3.8%)		

For peer review only

Figures are presented as mean ±SD

FEV1: Forced Expiratory Volume in 1 second, FVC: Forced Vital Capacity, VC%: % Vital Capacity

FEV1/FVC (%) Forced Expiratory Volume in 1 second.

CB: Chronic Bronchitis BA: Bronchial Asthma PE: Pulmonary Emphysema

CB+BA CB+PE BA+PE

Differences in sample characteristics analysed using the Kruskal-Wallis test with Cochran-Armitage tests for significance

post-hoc tests: Mann-Whitney U test, chi-square test for significance

a: Group A vs Group B

b:Group C vs Group D

c: Group A vs Group C

d: Group B vs Group D

e: Group A vs Group D

f:Group B vs Group C

a, b: Current smokers / ex-smokers (A and C Groups) vs never smokers (B and D Groups)

c, d: Diagnosis of obstructive ventilatory defect (A and B Groups) vs no obstructive ventilatory defect (C and D Groups)

For peer review only

Table 2. Change in demographic characteristics and respiratory function of designated air pollution victims by comparison group between initial evaluation and follow-up

		Initial evaluation	Follow-up	p	Initial evaluation	Follow-up	p	Initial evaluation	Follow-up	p	Initial evaluation	Follow-up	p
		Group A			Group B			Group C			Group D		
Age		54.3 ± 8.3	78.6 ± 6.5	<.001	49.6 ± 8.2	77.3 ± 7.5	<.001	49.8 ± 7.8	74.2 ± 7.1	<.001	51.3 ± 8.9	76.5 ± 7.8	<.001
Height	(cm)	161.7 ± 6.3	159.3 ± 6.9	<.001	155.2 ± 7.6	150.8 ± 8.8	<.001	161.4 ± 6.1	159.2 ± 6.2	<.001	152.8 ± 7.5	148.9 ± 8.8	<.001
Weight	(kg)	56.9 ± 6.4	55.4 ± 9.2	<.001	55.4 ± 9.3	52.1 ± 10.9	<.001	59.1 ± 8.9	58.9 ± 9.6	<.001	55.2 ± 9.7	53.2 ± 11.3	<.001
BMI		21.8 ± 1.9	21.7 ± 2.9	<.001	22.8 ± 3.6	22.7 ± 3.9	<.001	22.6 ± 2.8	23.2 ± 3.1	<.05	23.4 ± 3.8	23.9 ± 4.2	0.13
PFT													
FVC	(l)	3.37 ± 0.79	2.54 ± 0.85	<.001	3.04 ± 0.79	2.21 ± 0.73	<.001	3.49 ± 0.76	2.78 ± 0.81	<.001	2.94 ± 0.68	2.28 ± 0.69	<.001
%VC	(%)	105.5 ± 18.7	93.1 ± 21.7	<.001	103.5 ± 18.5	90.3 ± 22.3	<.001	108.3 ± 15.5	100.2 ± 21.9	<.001	107 ± 17.3	100.4 ± 23.4	<.001
FEV1	(l)	2.09 ± 0.64	1.28 ± 0.53	<.001	1.72 ± 0.58	1.29 ± 0.51	<.001	2.67 ± 0.58	1.8 ± 0.68	<.001	2.24 ± 0.58	1.61 ± 0.52	<.001
FEV1 /FVC	(%)	64.9 ± 6.5	54.5 ± 2.3	<.001	59.8 ± 10.3	62.1 ± 13.4	<.001	79.1 ± 5.6	67.9 ± 10.2	<.001	79.6 ± 7.1	73.8 ± 13.8	<.001

PFT: Pulmonary Function Testing

Figures are presented as mean ±SD

Initial evaluation versus follow-up: Mann-Whitney U test

For peer review only

Table 3. Change in respiratory symptoms of designated air pollution victims by comparison group between initial evaluation and follow-up

	Unit Score	Initial evaluation		Follow-up		p	Initial evaluation		Follow-up		p	Initial evaluation		Follow-up		p	Initial evaluation		Follow-up		p
Dyspnoea		Group A					Group B					Group C					Group D				
	1	0	0.0%	1	2.1%		1	0.6%	2	1.2%		0	0.0%	1	0.8%		0	0.0%	0	0.0%	
	2	0	0.0%	4	8.3%		4	2.4%	15	8.9%		0	0.0%	2	1.7%		4	1.1%	13	3.7%	
	3	9	18.8%	24	50.0%	<.001	31	18.3%	74	43.8%	<.001	7	5.9%	20	16.8%	<.001	55	15.8%	67	19.2%	<.001
	4	30	62.5%	15	31.3%		111	65.7%	75	44.4%		75	63.0%	77	64.7%		246	70.5%	279	79.9%	
	5	9	18.8%	4	8.3%		22	13.0%	3	1.8%		37	31.1%	19	16.0%		89	25.5%	35	10.0%	
Asthma attack																					
	1	0	0.0%	0	0.0%		1	0.6%	0	0.0%		0	0.0%	0	0.0%		1	0.3%	0	0.0%	
	2	0	0.0%	3	6.3%		8	4.7%	19	11.2%		1	0.8%	4	3.4%		13	3.3%	10	2.5%	
	3	7	14.6%	16	33.3%	<.001	51	30.2%	72	42.6%	<.001	11	9.2%	17	14.3%	<.001	66	16.8%	71	18.0%	0.78
	4	14	29.2%	15	31.3%		76	45.0%	51	30.2%		30	25.2%	35	29.4%		139	35.3%	139	35.3%	
	5	27	56.3%	14	29.2%		33	19.5%	27	16.0%		77	64.7%	63	52.9%		175	44.4%	174	44.2%	
Cough and phlegm																					
	1	0	0.0%	1	2.1%		2	1.2%	1	0.6%		0	0.0%	0	0.0%		0	0.0%	0	0.0%	
	2	0	0.0%	3	6.3%		5	3.0%	8	4.7%		1	0.8%	2	1.7%		9	2.3%	9	2.3%	
	3	24	50.0%	34	70.8%	<.001	50	29.6%	99	58.6%	<.001	58	48.7%	66	55.5%	<.001	143	36.3%	155	39.3%	0.24
	4	23	47.9%	9	18.8%		90	53.3%	55	32.5%		58	48.7%	49	41.2%		227	57.1%	221	56.1%	
	5	1	2.1%	1	2.1%		22	13.0%	6	3.6%		2	0.8%	2	1.7%		15	3.8%	9	2.3%	

Respiratory symptoms

Numerical values are shown (%).

Initial evaluation versus follow-up: Wilcoxon signed-rank test

For peer review only - http://bmjopen.bmj.com/site/about/guidelines.xhtml

Table 4. Mean annual change in yearly spirometry results from initial evaluation to follow-up by comparison group

		Group A (n=48)	Group B (n=169)	Group C (n=119)	Group D (n=394)	p	post-hoc test
FEV1 decline/year	(ml)	-36.7±19.1	-26.5±18.5	-37.2±16.4	-25.5±17.2	<.001	a.b.d.e.f
FEV1/FVC decline/year	(%)	-0.04±0.05	0.09±0.06	-0.04±0.03	-0.02±0.04	<.001	a.b.d.e.f
FVC decline/year	(ml)	-31.7±24.5	-30.1±20.7	-29.9±22.8	-25.3±17.1	<.05	d
%VC decline/year	(%)	-0.05±0.08	-0.05±0.08	-0.03±0.06	-0.03±0.07	<.001	c.d.e.f

Figures are presented as mean ±SD

p-values were calculated using the Kruskal-Wallis test post-hoc test: Mann-Whitney U test

a: Group A vs Group B

b:Group C vs Group D

c: Group A vs Group C

d: Group B vs Group D

e: Group A vs Group D

f:Group B vs Group C

a, b: Current smokers / ex-smokers (A and C Groups) vs never smokers (B and D Groups)

c, d: Diagnosis of obstructive ventilatory defect (A and B Groups) vs no obstructive ventilatory defect (C and D Groups)

For peer review only

Table 5. Change in severity of smoking-related respiratory symptoms by comparison group at follow-up

Score	Group A (n=48)		Group B (n=169)		Group C (n=119)		Group D (n=394)		p	post-hoc test
Dyspnea										
1	1	2.1%	2	1.2%	1	0.8%	0	0.0%		
2	4	8.3%	15	8.9%	2	1.7%	13	3.7%		
3	24	50.0%	74	43.8%	20	16.8%	67	19.2%	<.001	c.d.e.f
4	15	31.3%	75	44.4%	77	64.7%	279	79.9%		
5	4	8.3%	3	1.8%	19	16.0%	35	10.0%		
Asthma attack										
1	0	0.0%	0	0.0%	0	0.0%	0	0.0%		
2	3	6.3%	19	11.2%	4	3.4%	10	2.5%		
3	16	33.3%	72	42.6%	17	14.3%	71	18.0%	<.001	a.c.d.e.f
4	15	31.3%	51	30.2%	35	29.4%	139	35.3%		
5	14	29.2%	27	16.0%	63	52.9%	174	44.2%		
Cough and phlegm										
1	1	2.1%	1	0.6%	0	0.0%	0	0.0%		
2	3	6.3%	8	4.7%	2	1.7%	9	2.3%		
3	34	70.8%	99	58.6%	66	55.5%	155	39.3%	<.001	a.b.c.d.e.
4	9	18.8%	55	32.5%	49	41.2%	221	56.1%		
5	1	2.1%	6	3.6%	2	1.7%	9	2.3%		

Numerical values are shown (%)

p-values were calculated using the Kruskal-Wallis test   post-hoc test: Mann–Whitney U test

a: Group A vs Group B

b:Group C vs Group D

c: Group A vs Group C

d: Group B vs Group D

e: Group A vs Group D

f:Group B vs Group C

a, b: Current smokers / ex-smokers (A and C Groups) vs never smokers (B and D Groups)

c, d: Diagnosis of obstructive ventilatory defect (A and B Groups) vs no obstructive ventilatory defect (C and D Groups)

For peer review only - http://bmjopen.bmj.com/site/about/guidelines.xhtml

Figure legends

Figure 1 Flowchart showing the selection procedure for study subjects.

The study included officially designated victims of pollution-related illness in Kurashiki aged 65 years or older in 2009, and for whom full data were available for statistical analysis.

Ceased to experience symptoms : Officially designated victims who did renew the designation because of disappearance of clinical symptoms.

Figure 2 Sulfur dioxide and nitrogen dioxide concentrations from 1965 to 2009 relative to standard values.

REFERENCES

1. Yang G, Wang Y, Zeng Y, et al. Rapid health transition in China, 1990-2010: findings from the Global Burden of Disease Study 2010. Lancet 2013;**381**(9882):1987-2015 doi: 10.1016/S0140-6736(13)61097-1
S0140-6736(13)61097-1 [pii][published Online First: Epub Date]|.
2. Liu Y, Lee K, Perez-Padilla R, et al. Outdoor and indoor air pollution and COPD-related diseases in high- and low-income countries. Int J Tuberc Lung Dis 2008;**12**(2):115-27
3. Yang JY, Xin JY, Ji DS, et al. [Variation analysis of background atmospheric pollutants in North China during the summer of 2008 to 2011]. Huan Jing Ke Xue 2012;**33**(11):3693-704
4. Tanaka T, Asai M, Yanagita Y, et al. Longitudinal study of respiratory function and symptoms in a non-smoking group of long-term officially-acknowledged victims of pollution-related illness. BMC Public Health 2013;**13**:766 doi: 10.1186/1471-2458-13-766
1471-2458-13-766 [pii][published Online First: Epub Date]|.
5. Usmanova G, Mokdad AH. Results of the Global Youth Tobacco Survey and implementation of the WHO Framework Convention on Tobacco Control in the WHO Eastern Mediterranean Region (EMR) countries. J Epidemiol Glob Health 2013;**3**(4):217-34 doi: 10.1016/j.jegh.2013.07.001
S2210-6006(13)00084-1 [pii][published Online First: Epub Date]|.
6. Yang G, Fan L, Tan J, et al. Smoking in China: findings of the 1996 National Prevalence Survey. JAMA 1999;**282**(13):1247-53 doi: joc80784 [pii][published Online First: Epub Date]|.
7. M C. WHO report on the global tobacco epidemic 2008. Geneva:World Health Organization 2008
8. Yamagata T, Hirano T, Sugiura H, et al. Comparison of bronchodilatory properties of transdermal and inhaled long-acting beta 2-agonists. Pulm Pharmacol Ther 2008;**21**(1):160-5 doi: S1094-5539(07)00047-8 [pii]
10.1016/j.pupt.2007.05.004[published Online First: Epub Date]|.

For peer review only - http://bmjopen.bmj.com/site/about/guidelines.xhtml

9. Baldwin ED CA, Richards DW Jr. Pulmonary insufficiency; physiological classification, clinical methods of analysis, standard values in normal subjects. Medicine (Baltimore) 1948; **27(3)**:243-78
10. Curjuric I, Imboden M, Nadif R, et al. Different genes interact with particulate matter and tobacco smoke exposure in affecting lung function decline in the general population. PLoS One 2012;**7**(7):e40175 doi: 10.1371/journal.pone.0040175 PONE-D-11-21280 [pii][published Online First: Epub Date]|.
11. Ceylan E, Kocyigit A, Gencer M, et al. Jul 2006 patent 0954-6111 (Print) 0954-6111 (Linking).
12. Downs SH, Schindler C, Liu LJ, et al. Reduced exposure to PM10 and attenuated age-related decline in lung function. N Engl J Med 2007;**357**(23):2338-47 doi: 357/23/2338 [pii] 10.1056/NEJMoa073625[published Online First: Epub Date]|.
13. Lee PN, Fry JS. Systematic review of the evidence relating FEV1 decline to giving up smoking. BMC Med 2010;**8**:84 doi: 10.1186/1741-7015-8-84 1741-7015-8-84 [pii][published Online First: Epub Date]|.
14. Fletcher C, Peto R. The natural history of chronic airflow obstruction. British medical journal 1977;**1**(6077):1645-8
15. Downs SH, Schindler C, Liu LJ, et al. Reduced exposure to PM10 and attenuated age-related decline in lung function. N Engl J Med 2007;**357**(23):2338-47 doi: 10.1056/NEJMoa073625[published Online First: Epub Date]|.
16. Siroux V, Pin I, Oryszczyn MP, et al. Relationships of active smoking to asthma and asthma severity in the EGEA study. Epidemiological study on the Genetics and Environment of Asthma. Eur Respir J 2000;**15**(3):470-7
17. Gruffydd-Jones K. GOLD guidelines 2011: what are the implications for primary care? Primary care respiratory journal : journal of the General Practice Airways Group 2012;**21**(4):437-41 doi: 10.4104/pcrj.2012.00058[published Online First: Epub Date]|.
18. Pelkonen M, Notkola IL, Nissinen A, et al. Thirty-year cumulative incidence of chronic bronchitis and COPD in relation to 30-year pulmonary function and 40-year mortality: a follow-up in middle-aged rural men. Chest 2006;**130**(4):1129-37 doi: 130/4/1129 [pii] 10.1378/chest.130.4.1129[published Online First: Epub Date]|.
19. Berglund DJ, Abbey DE, Lebowitz MD, et al. Respiratory symptoms and pulmonary function in an elderly nonsmoking population. Chest 1999;**115**(1):49-59
20. Oga T TM, Hajiro T, Ikeda A, Nishimura K. Analysis of longitudinal changes in dyspnea of patients with chronic obstructive pulmonary disease: an observational study. Respir Res 2012;**13**:85
21. Dong GH, Zhang P, Sun B, et al. Long-term exposure to ambient air pollution and respiratory disease mortality in Shenyang, China: a 12-year population-based retrospective cohort study. Respiration; international review of thoracic diseases 2012;**84**(5):360-8 doi: 10.1159/000332930[published Online First: Epub Date]|.
22. C. Arden Pope I caRTB, Michelle C. Turner, Aaron Cohen, Daniel Krewski, Michael Jerrett, Susan M. Gapstur, and Michael J. Thun. Lung Cancer and Cardiovascular Disease Mortality Associated with Ambient Air Pollution and Cigarette Smoke: Shape of the Exposure–Response Relationships. Environ Health Perspect 2011 **119(11)**: 1616–21
23. Kaufman G. Chronic obstructive pulmonary disease: diagnosis and management. Nurs Stand 2013;**27**(21):53-7, 60-2
24. website.http GIfCOLDG, //www.goldcopd.org/. Global strategy for the diagnosisGlobal strategy for the diagnosis. management and prevention of COPD, 2011.

For peer review only - http://bmjopen.bmj.com/site/about/guidelines.xhtml

For peer review only

For peer review only - http://bmjopen.bmj.com/site/about/guidelines.xhtml

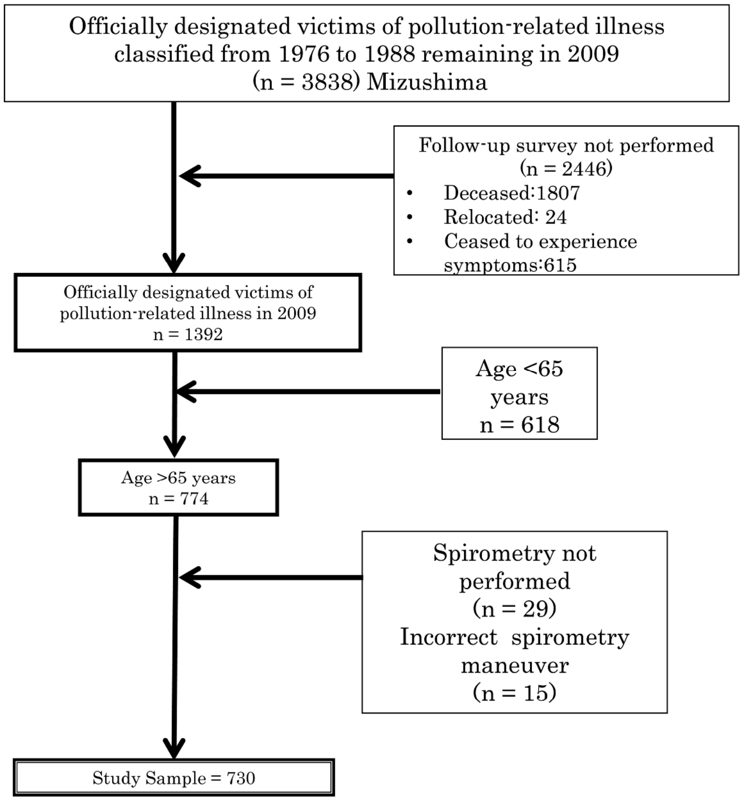

Figure 1: Flowchart showing the selection procedure for study subjects. The study included officially designated victims of pollution-related illness in Kurashiki aged 65 years or older in 2009, and for whom full data were available for statistical analysis.
Ceased to experience symptoms: Officially designated victims who did renew the designation because of disappearance of clinical symptoms.

254x338mm (300 x 300 DPI)

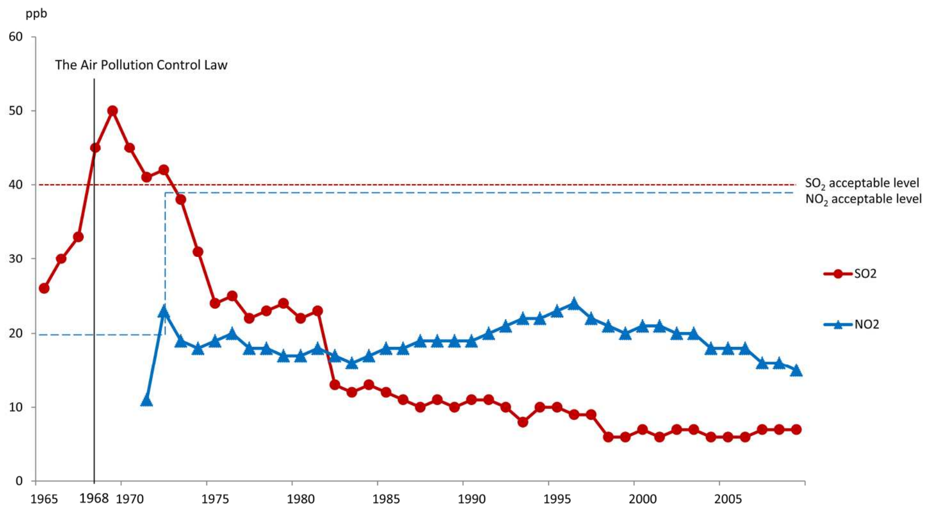

Figure 2 levels of air pollution

190x142mm (300 x 300 DPI)

iew only

STROBE 2007 (v4) Statement—Checklist of items that should be included in reports of *cohort studies*

Section/Topic	Item #	Recommendation	Reported on page #
**Title and abstract**	1	(*a*) Indicate the study's design with a commonly used term in the title or the abstract	
		(*b*) Provide in the abstract an informative and balanced summary of what was done and what was found	
**Introduction**			
Background/rationale	2	Explain the scientific background and rationale for the investigation being reported	
Objectives	3	State specific objectives, including any prespecified hypotheses	
**Methods**			
Study design	4	Present key elements of study design early in the paper	
Setting	5	Describe the setting, locations, and relevant dates, including periods of recruitment, exposure, follow-up, and data collection	
Participants	6	(*a*) Give the eligibility criteria, and the sources and methods of selection of participants. Describe methods of follow-up	
		(*b*) For matched studies, give matching criteria and number of exposed and unexposed	
Variables	7	Clearly define all outcomes, exposures, predictors, potential confounders, and effect modifiers. Give diagnostic criteria, if applicable	
Data sources/ measurement	8*	For each variable of interest, give sources of data and details of methods of assessment (measurement). Describe comparability of assessment methods if there is more than one group	
Bias	9	Describe any efforts to address potential sources of bias	
Study size	10	Explain how the study size was arrived at	
Quantitative variables	11	Explain how quantitative variables were handled in the analyses. If applicable, describe which groupings were chosen and why	
Statistical methods	12	(*a*) Describe all statistical methods, including those used to control for confounding	
		(*b*) Describe any methods used to examine subgroups and interactions	
		(*c*) Explain how missing data were addressed	
		(*d*) If applicable, explain how loss to follow-up was addressed	
		(*e*) Describe any sensitivity analyses	
**Results**			

For peer review only

Participants	13*	(a) Report numbers of individuals at each stage of study—eg numbers potentially eligible, examined for eligibility, confirmed eligible, included in the study, completing follow-up, and analysed	
		(b) Give reasons for non-participation at each stage	
		(c) Consider use of a flow diagram	
Descriptive data	14*	(a) Give characteristics of study participants (eg demographic, clinical, social) and information on exposures and potential confounders	
		(b) Indicate number of participants with missing data for each variable of interest	
		(c) Summarise follow-up time (eg, average and total amount)	
Outcome data	15*	Report numbers of outcome events or summary measures over time	
Main results	16	(*a*) Give unadjusted estimates and, if applicable, confounder-adjusted estimates and their precision (eg, 95% confidence interval). Make clear which confounders were adjusted for and why they were included	
		(*b*) Report category boundaries when continuous variables were categorized	
		(*c*) If relevant, consider translating estimates of relative risk into absolute risk for a meaningful time period	
Other analyses	17	Report other analyses done—eg analyses of subgroups and interactions, and sensitivity analyses	
**Discussion**			
Key results	18	Summarise key results with reference to study objectives	
**Limitations**			
Interpretation	20	Give a cautious overall interpretation of results considering objectives, limitations, multiplicity of analyses, results from similar studies, and other relevant evidence	
Generalisability	21	Discuss the generalisability (external validity) of the study results	
**Other information**			
Funding	22	Give the source of funding and the role of the funders for the present study and, if applicable, for the original study on which the present article is based	

*Give information separately for cases and controls in case-control studies and, if applicable, for exposed and unexposed groups in cohort and cross-sectional studies.

Note: An Explanation and Elaboration article discusses each checklist item and gives methodological background and published examples of transparent reporting. The STROBE checklist is best used in conjunction with this article (freely available on the Web sites of PLoS Medicine at http://www.plosmedicine.org/, Annals of Internal Medicine at http://www.annals.org/, and Epidemiology at http://www.epidem.com/). Information on the STROBE Initiative is available at www.strobe-statement.org.

For peer review only
